# *S*-(−)-Oleocanthal Ex Vivo Modulatory Effects on Gut Microbiota

**DOI:** 10.3390/nu15030618

**Published:** 2023-01-25

**Authors:** Mohammed H. Qusa, Khaldoun S. Abdelwahed, Ronald A. Hill, Khalid A. El Sayed

**Affiliations:** School of Basic Pharmaceutical and Toxicological Sciences, College of Pharmacy, University of Louisiana at Monroe, 1800 Bienville Drive, Monroe, LA 71201, USA

**Keywords:** extra-virgin olive oil, fecal fermentation, microbiota, *S*-(−)-oleocanthal, shotgun analysis

## Abstract

Compelling evidence points to the critical role of bioactive extra-virgin olive oil (EVOO) phenolics and gut microbiota (GM) interplay, but reliable models for studying the consequences thereof remain to be developed. Herein, we report an optimized ex vivo fecal anaerobic fermentation model to study the modulation of GM by the most bioactive EVOO phenolic *S*-(−)-oleocanthal (OC), and impacts therefrom, focusing on OC biotransformation in the gut. This model will also be applicable for characterization of GM interactions with other EVOO phenolics, and moreover, for a broadly diverse range of bioactive natural products. The fecal fermentation media and time, and mouse type and gender, were the major factors varied and optimized to provide better understanding of GM-OC interplay. A novel resin entrapment technique (solid-phase extraction) served to selectively entrap OC metabolites, degradation products, and any remaining fraction of OC while excluding interfering complex fecal medium constituents. The effects of OC on GM compositions were investigated via shallow shotgun DNA sequencing. Robust metabolome analyses identified GM bacterial species selectively altered (population numbers/fraction) by OC. Finally, the topmost OC-affected gut bacterial species of the studied mice were compared with those known to be extant in humans and distributions of these bacteria at different human body sites. OC intake caused significant quantitative and qualitative changes to mice GM, which was also comparable with human GM. Results clearly highlight the potential positive health outcomes of OC as a prospective nutraceutical.

## 1. Introduction

Interest in extra-virgin olive oil (EVOO) phenolics research has sharply gained in prominence in recent years, with compelling evidence suggesting that its consumption is singly or synergistically responsible for multiple observed positive health benefits [1]. The EVOO phenolics are among the most thoroughly studied bioactive dietary ingredients over the past decade [1]. The monophenolic *S*-(−)-oleocanthal (OC), the dialdehydic form of (−)-deacetoxy-ligstroside aglycone, has been nominated as the topmost bioactive phenolic ingredient of EVOO [2,3]. Biological effects of OC against inflammation, oxidative stress, infections, Alzheimer’s disease, and cancers have been amply documented [2,3]. Importantly, the bioavailability and major intracellular molecular mechanisms of OC have been extensively studied in an array of in vitro and in vivo models, wherein OC has been seen to generate distinct and somewhat diverse or disparate pharmacodynamics profiles [3,4,5]. Despite the chemical and metabolic peculiarity of OC attributing to its two vicinal and highly reactive aldehyde groups, notable progress has been achieved in overcoming the significant challenges faced in devising and developing reliable models to study OC absorption into the human bloodstream following extravascular administration (p.o. or i.p.) and its subsequent fate [6,7,8]. However, even for intravascular administration, the metabolic fate of OC remains unclear, posing a major obstacle to developing OC as a prospective FDA-approved drug or nutraceutical.

Protected by the monounsaturated fatty acid triglycerides matrix of EVOO, simple EVOO tyrosol and hydroxytyrosol-based phenolics can be rapidly absorbed in the small intestine, unlike the more complex phenolics, which can reach, at least in-part, the large intestine, where they can interact with host gut microbiota (GM) [9]. However, relatively modest scientific efforts have been devoted to investigating OC modulatory effects on GM [9,10]. Thus, we considered devising and validating reliable models that could aid in understanding OC interplay with GM to be vital in addressing key unknowns associated with various unexplained in vivo findings, and towards optimizing OC formulation and beneficial uses in human health. A validated cause-and-effect pattern has been observed connecting the consumption of EVOO phenolics to positive health impacts, including improved endothelial function and prevention of deleterious low-density lipoprotein (LDL) oxidation [11]. Reasonably, the decisive drivers of these beneficial effects can be attributed in whole or in-part to either GM production of specific metabolite(s), and/or to relevant GM-associated metabotypes [11]. Consumption of a recommended dose of olive and other oils produced health benefits unlike higher concentrations; in fact, an intake dose three-fold above that regarded to be in a more normal range suppressed the microbial alpha-diversity profoundly and to a likely deleterious degree [12]. The major olive leaf phenolic oleuropein was recently reported to alter levels of GM of the Coriobacteriaceae and Lactobacillaceae families and the genus *Collinsella*, attributable to the fermentation of olive leaf extract by feces [13]. This fermentation resulted in a significant decrease in oleuropein and an increase in hydroxytyrosol levels in fermented olive leaf extract [13]. Olive phenolics are reported to modulate GM short-chain fatty-acids, which beneficially impacts cholesterol trafficking and metabolism, immune system function, and weight gain via promoting satiety [9]. On the other hand, GM diversity significantly increased in the feces of rats fed a high phenolic EVOO diet [9]. EVOO-induced GM diversity enhancement was linked to beneficial reduction of systolic blood pressure in spontaneously hypertensive rats [9]. Olive oil consumption in suitable regimens can alter colonic GM constitution and activity, leading to prospective colon cancer prevention [14]. Studying OC modulatory effects on GM stands to substantially contribute to increased understanding of the considerable array of biological effects seen for EVOO that are of compelling prospective benefit to human health, provided such knowledge is sufficient and sound enough to inform and guide applications.

Despite the high potencies observed for various effects and actions brought about by OC exposures/administration, the identities of possible pharmacophores to which these may be attributed remain to be established, a goal confounded especially by the above-noted chemical and biochemical lability of OC, as well as by the immediate hydration of its *C*-1 and *C*-3 aldehydes to a *gem*-diol form in water, and rapid acetalization in the presence of alcohols [8]. OC also spontaneously reacts with glycine, and with the ε-amino moiety of lysines in proteins in animal and human body organs and in circulating blood, making its detection in blood nearly impossible even at high doses [8]. Moreover, and as with virtually all aldehydes, reaction with glutathione and with free thiols of proteins including hemoglobin is also expected to be pervasive and extensive, albeit reversible, and aldehyde oxidases and various reductases are also prevalent in blood and tissues. Consequently, it is important to understand if OC would modulate GM taxonomical distribution and metabotypes independent of systemic OC metabolic fate following absorption. Here, we can also note that absorption of lipid-entrained OC via the chylomicron route is of likely importance, opening the possibility for enterohepatic cycling, which to our knowledge remains to be addressed. In the context of the above, we note that in a single-dose safety study of OC, Swiss albino mice tolerated oral doses of up to 500 mg/kg, whereas the intraperitoneal LD_50_ of OC was estimated to be in the range of 164–524 mg/kg [15]. This disparity clearly shows the great prospective importance of roles played by GM in the safety of OC and EVOO phenolics with oral administration/consumption.

Approaches employing GM, a heterogeneous assemblage of vitally useful, beneficial, and sometimes harmful microorganisms, have been developed and brought to bear extensively for validated biotransformation studies of numerous xenobiotics including bioactive food ingredients [16,17,18,19,20]. Most of the studies on xenobiotic-GM interactions have focused on methods of modulating the microbial community and, for drugs, enhancing efficacy or increasing safety [21]. However, the complex relationship between GM and the metabolic fate of xenobiotics including drugs and dietary bioactive compounds has recently been attracting increasing attention, with rapid gains in recognition and appreciation of its importance and impacts [22]. GM can influence metabolic fate through capability to either detoxify or to activate xenobiotics [23]. Diversity and richness of GM populations have also increasingly been established to be valid and surprisingly informative indicators of healthy body systems [16,17]. Roles of GM cross-play with several dietary polyphenolics, and physiological and microbiological impacts thereof, have been identified and characterized [17]. Hence, we considered ex vivo fermentation to be a viable approach [18] upon which reliable models might be based for investigating GM interactions with xenobiotics more generally, and for this work, EVOO constituents more specifically, focusing here on OC. Various polyphenols are documented substrates for biotransformation by, or act as biochemical modulators of, GM [19,20]. Fresh animal fecal suspensions or fecal enzymes under exceptionally controlled fermentation conditions, which mimic anaerobic intestinal environment, have been used to study such interplay between GM and compounds of interest, including various bioactive polyphenols [19,20]. Thus, we deemed that models involving animal feces would prospectively be acceptable for studying the alteration of the intestinal microflora composition by the bioactive polyphenols in EVOO. Moreover, detailed characterization of the microbiota using the aggregate metagenome assembly as proxy, via shotgun metagenomics sequencing, could serve to identify and highlight GM community changes, and associated functional correlates with xenobiotics digestion [16]. Shotgun analysis is one of the next-generation sequencing (NGS) techniques, offering the ability to sequence the whole microbiome DNA to obtain an aggregate genomic profile, based upon which the constitutional makeup of the microbiome can be characterized, as well as changes thereof in response to situational variables [19,20].

The current study aimed to ascertain and characterize the abilities of OC to modulate GM diversity, and in turn, the impacts on its own (OC) fate. We developed a fecal fermentation ex vivo model and optimized all pertinent variables, to enable and facilitate the study of OC-GM interactions. To achieve this aim, we completed the following:

(1) We designed and validated a detailed standard operating protocol, thereby achieving a rapid-analysis workflow for studying OC effects on GM via an ex vivo mouse fecal fermentation model, optimizing all associated factors to maximally reflect and closely mimic the living gut system.
(2) We developed protocols for the tailored use of shallow whole metagenome sequencing of optimized ex vivo fecal samples to characterize the GM constitution upon addition of OC, which was seen to comprehensively cause qualitative and quantitative GM changes. Further analysis documented OC effects on various GM species. With information linking these species to corresponding distribution sites (known or predicted) within the human microbiome, it becomes possible to envision prospective human health impacts of such changes.

Examples of current in vitro and ex vivo models that can simulate the in vivo behavior of the GM ecosystem are batch fermentation models, dynamic fermentation models, the proprietary TNO in vitro model of the colon, and the simulator of the human intestinal microbial ecosystem [24]. The basic rationale for these models is that they provide validated platforms for studying xenobiotic biotransformation by maintaining freshly sampled fecal GM under controlled environmental conditions, enabling study of the formation of biotransformation products (“metabolites”) over time, and enabling the identification of GM-produced metabolites of new or unstudied xenobiotics [24]. However, no current models have emerged involving the clear and incisive identification of microbiota taxonomical changes as might be induced by exposure to the xenobiotic, which may in turn translate to consequential pharmacodynamic or pharmacokinetics-pharmacodynamics impacts. 

**Figure 1:**
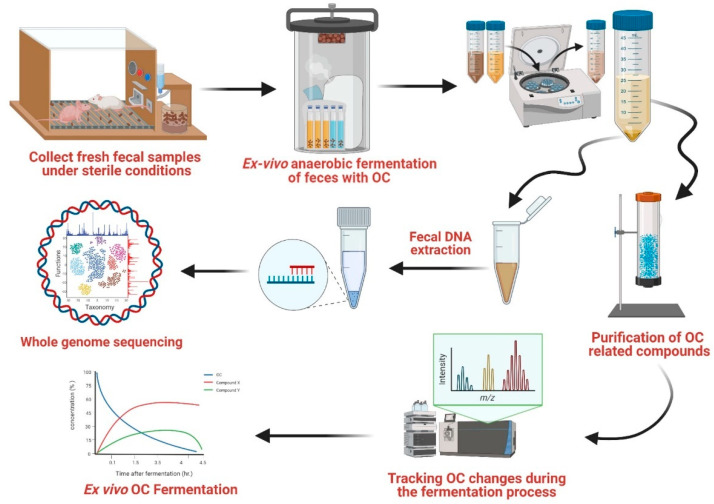
OC-GM interplay study design.

Herein, we present results generated with an elaborated and expanded approach to conventional biological functional modules, along with an inventory of all identified key impacting factors, for studying the mutual interplay of the EVOO bioactive phenolic OC with GM. To wit, we have brought to bear our novel ex vivo fermentation culture in conjunction with the use of metagenomics towards gaining better understanding of the modulatory impact of OC on GM taxonomy (Figure 1), and in turn, the likely impacts of these changes on the fate of OC pursuant to its oral administration.

## 2. Materials and Methods

### 2.1. Oleocanthal Extraction and Purification Process

*S*-(−)-Oleocanthal (OC) was extracted from EVOO (Governor, Corfu, Greece) following the methodology described earlier [25]. Briefly, EVOO was vigorously shaken with twice its volume of water in a separatory funnel to achieve adequate liquid-liquid extraction. The aqueous layer was collected and passed through Sorbtech resin (Sorbent Technology Sepabeads Resin Styrenic Adsorbent Sp-70-01, No. 46410-A1, Sorbent Technologies, East Norcross, GA, USA) to retain OC and other phenolics, which were subsequently eluted by 100% acetone. The acetone layer was evaporated under vacuum and the semisolid residue was vigorously shaken with dichloromethane and dehydrated over anhydrous Na_2_SO_4_ to remove any residual water. The crude extract was then packed into a Sephadex LH-20 column (1:100 ratio) with a bead size of 25–100 μm (No. LH20100, Sigma Aldrich, St. Louis, MO, USA) to purify OC by isocratic elution with CH_2_Cl_2_. A purity of >99% was established for OC based on q^1^H NMR and HPLC analyses [25]. All solvents used in extraction were ACS grade purchased from VWR International (Suwanee, GA, USA).

### 2.2. Housing, Diets, and Animal Model’s Experimental Setup

The reported studies were carried out with 4–5-week-old male and female ND4 Swiss albino (SA) outbred mice (*n* = 10 male, 9 female) and Foxn1^nu^/Foxn^1+^ athymic nude mice (*n* = 10 male, 10 female) purchased from Envigo (Indianapolis, IN, USA). All animals were acclimated to the vivarium and maintained under cleanroom conditions in sterile filter-top cages with Alpha-Dri bedding housed on high-efficiency particulate-filtered air-ventilated racks at 25 °C, 55–65% relative humidity, and 12 h light/dark cycle for a week before experiments. The mice had free access to drinking water and pelleted rodent chow (No. 7012, Harlan/Teklad, Madison, WI, USA). 

### 2.3. Ethics Statement

All animal experiments were approved by the Institutional Animal Care and Use Committee, University of Louisiana at Monroe, protocol number 17OCT-KES-01, and were handled in strict accordance with good animal practice as defined by the NIH guidelines.

### 2.4. Ex Vivo Fecal Fermentation 

To simulate the gut environment, all in vitro/ex vivo experimental procedures were carried out under controlled anaerobic fermentation conditions, including optimal growth media, air-gas levels, pH, and temperature, in a sterilized culture model [26,27]. Fresh feces samples collected within 30–60 min of defecation of 9–10 mice/group were mixed and pooled in one sample for each group. Groups were representative of the following: male nude mice, female nude mice, male SA mice, female SA mice. Feces samples were collected under aseptic conditions (inside sterile mouse cages and a laminar flow hood). Each group’s pooled fecal sample was suspended directly in an appropriate sterile growth medium, Brain Heart Infusion Broth (BHI, B9500, Teknova, Hollister, CA, USA), prepared according to the manufacturer’s instructions, or in a maintenance medium, normal saline (0.9% sodium chloride), to preserve the viability of microorganisms. Fecal suspension (2.5% *w*/*v* per tube) was vortexed for 5 min at room temperature to create fecal slurries, breaking all individual fecal pellets’ shells. Fecal slurry stocks were then divided into fermentation tubes. Each 1 mL of fecal slurry (containing 250 mg of feces) was constituted to a volume of 10 mL with the chosen medium, to which 10 mg of OC was added. Sterile deionized water alone was added to the vehicle control sample. To attain anaerobic conditions, all fermented samples were cultured in tightly sealed GENbag anaerobic bags (No. 45534, BioMerieux, Durham, NC, USA, which reached less than 0.1% oxygen level in 1.5 h per manufacturer specifications), in which two CO_2_ generators were added. The bags were then incubated at 37.7 °C for different durations: 0.1, 1.5, 3.5, and 4.5 h.

### 2.5. Fermentation Samples Preparation 

Once OC fermentation was continued to each specified time-point, samples were centrifuged, decanted, and the aqueous layer containing the OC outputs subjected to solid-phase extraction by passing the decanted solution over a syringe column water-packed with 5 g Sorbtech Sp70 entrapment resin, which was shown to have robust entrapment affinity for EVOO phenolic outputs [25,28], enabling the elimination of the main non-phenolic fecal constituents. The Sp70 resin column was washed with 5 mL of deionized water, and the residuals were then eluted with 15 mL of acetone. The acetone eluate rich in OC-phenolic conversion products was collected, freeze-dried, and stored under N_2_ gas at −80 °C until analysis. Each residual fecal sediment pellet was then washed with normal saline (NS) and stored frozen at −80 °C for subsequent bacterial DNA extraction.

### 2.6. DNA Extraction of Fermented Fecal Samples 

DNA extraction from treated and untreated fecal samples was carried out manually using the Ultrapure Microbial DNA Isolation Kit (12224-250, MO BIO, Jackson, MS, USA), following the manufacturer’s protocol but with minor modifications [29]. Briefly, 300 mg of each fecal sample was resuspended in its respective culture media and subjected to centrifugation at 15,000 RPM for 2 min. The supernatant was then decanted. The pellets were parsed into two portions, and each was resuspended in 300 μL of Micro Bead Solution with the addition of 50 μL MD1 solution. To increase the extraction yield and decrease DNA shearing, the Micro Bead vials were tightly secured and heated at 70 °C for 10 min with vortexing for a few seconds every 2 min. The suspension was then centrifuged, and approximately 600 μL of the supernatant was transferred to a collection tube. Purification of DNA from the fecal samples by non-DNA precipitation was conducted by adding 250 μL of MD2 solution to the main tube, which was then vortexed and incubated at 4 °C for 5 min. The solution was centrifuged, and the supernatant was mixed with a highly concentrated salt solution (MD3 solution). DNA was selectively trapped in a spin filter (silica membrane) by centrifugation at 15,000 RPM for 1 min, with 3X washing by EtOH for further DNA purification. Finally, 150 μL of elution buffer (Tris buffer; pH = 8) was added to release the DNA from the membrane, and the resulting eluates were stored at −80 °C until further analysis. A NanoDrop™ Microvolume UV-Vis Spectrophotometer (Thermo Scientific, Memphis, TN, USA) was used to quantify the extracted DNA samples for quality assurance before submission to the shallow shotgun analysis. In addition, DNA was qualitatively evaluated via gel electrophoresis, and quantified with the use of a Qubit 3.0 fluorometer (Thermo-Fischer, Waltham, MA, USA). Libraries were prepared using an Illumina Nextera library preparation kit using standard protocol (Illumina, San Diego, CA, USA).

### 2.7. Taxonomy Profiling by Shotgun-Based Next-Generation Sequencing

A NextSeq 500 Sequencing System was utilized to sequence both ends of a fragment and to create high-quality paired-end sequencing (150 bp × 2) in medium output mode. Shotgun metagenomic sequence reads were processed by the extensible Sunbeam pipeline. The quality of the raw sequence data was assessed by FastQC version 0.11.5 software [30]. The procedure was carried out in four steps: adapter removal, read trimming, low-complexity-reads removal, and host-sequence removals. Because only the genes of the microbial community were targeted, high-quality reads were mapped to two NCBI taxonomic annotation databases in accord with the fecal sourcing: the human genome (Genome Reference Consortium Human Build 37 (GRCh37) and the mouse genome (Genome Reference Consortium Mouse Build 38 patch release 6 (GRCm38.p6). Reads that mapped to these were removed from the analysis. After these quality control steps, the median number of quality-filtered reads per sample was 3,040,870. The reads were taxonomically aligned using the Kraken2 algorithm with the MiniKraken v1 database.

For shotgun functional profiling, high-quality reads were aligned after filtration against the SEED database (hierarchical functional tree) via translated homology search, and annotated to SEED subsystems classification (functional levels) 1 and 3 using Super-Focus 5 as the fastest agile functional analysis tool [31].

### 2.8. Diversity and Differential Abundance Analysis

Focusing on bacterial species-level analysis, alpha diversity was calculated based on taxonomic profiles using Shannon’s diversity index. Samples were distributed by H-statistic of the Kruskal–Wallis test (Chi-squared). Statistical analysis of beta diversity was performed by using Bray–Curtis dissimilarities, and samples were visualized using nonmetric multidimensional scaling (NMDS).

Negative binomial models (DESEq2 R package) were used for differential abundance testing of taxonomic and subsystem level 3 features. We looked for changes caused by OC treatments versus vehicle control after controlling for all sample variables, including mouse species and sex. *p*-values were calculated, with standard tests for comparison of control groups and likelihood-ratio test (LRT) for treatment evaluation. We then identified for further scrutiny the ten topmost significant taxa (based on the *p*-values = 0.1) that increased and decreased with OC treatment.

### 2.9. Correlation of the OC-Affected GM with the Human Microbiota

The effects of the OC addition on the distribution of microorganisms following incubation to various time points (as listed above) were analyzed for a subset of organisms identified in the human microbiome project (https://www.hmpdacc.org/hmp/HMASM/, accessed on 15 May 2021). The 690 samples that passed quality control were compared against the same classification database (MiniKraken, https://ccb.jhu.edu/software/kraken/, accessed on 15 May 2021). We compared the identities of the OC-affected microorganisms to their proposed distributions in the human body (organs/target human body sites), including gingival plaque, buccal mucosa, saliva, tongue dorsum, palatine tonsils, throat, and stool. Thus, prevalence (presence/absence) as well as mean abundances were calculated for the taxa with stratification by body location. We calculated the Spearman correlation for the 20 selected taxa in the HMP database using *z*-score normalized abundances (https://hmpdacc.org/, accessed on 15 May 2021). 

## 3. Results

### 3.1. Ex Vivo Fecal Fermentation Model for Identification of GM Metabolites 

**Figure 2:**
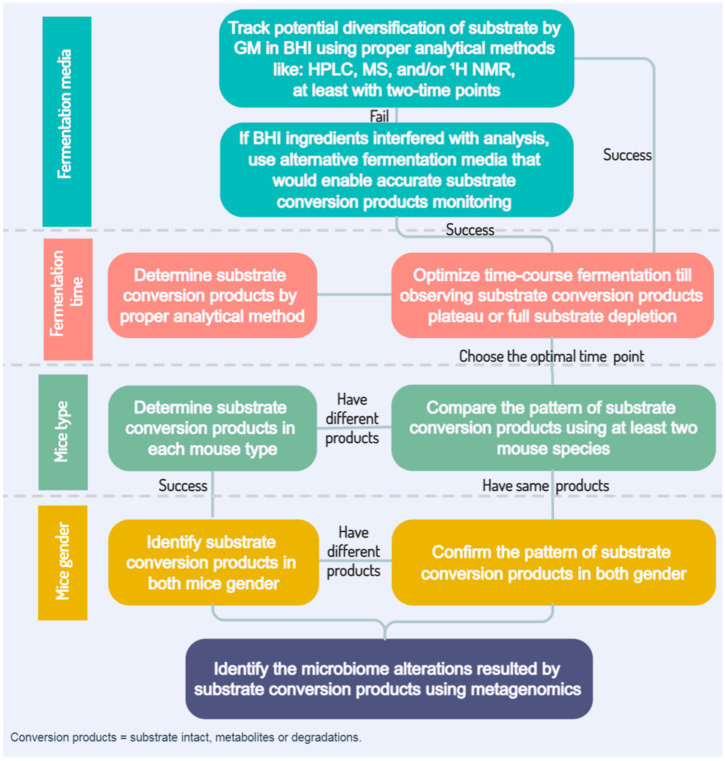
Overview flowchart of ex vivo GM-xenobiotic cross-interaction model.

Various potential confounding factors can be discerned with respect to ideally studying the transactional interplay of xenobiotics with GM [24]. However, the studies we report herein demonstrated that fecal fermentation medium, time, and mouse type (species, subspecies, strain, colony) and gender are the most critical factors to be optimized (cf. Figure 2). BHI medium was adopted with the aims of maintaining GM integrity and viability, and proved to be a suitable and possibly ideal fermentation growth medium under anaerobic conditions (but vide supra). 

### 3.2. Optimization of OC-GM Interactive Model

To devise and develop a model aimed at assuring maximal OC-GM interplay, pooled fresh mouse GM fecal samples were incubated with various concentrations of OC under optimized conditions. Fresh fecal samples were collected separately from mice of each gender of athymic nude and Swiss albino (SA) mice, with varied microbiome backgrounds (based on post hoc analysis), to optimize all factors that could contribute to OC-GM interaction. As noted in the Methods section above, fecal samples were used to generate an ex vivo fermentation model in BHI broth inside the GENbag, mimicking the anaerobic intestinal fermentation conditions to maintain the GM integrity and viability. Sterilized fecal samples, produced by autoclaving for 30 min at 121 °C and 15 psi, served as negative culture controls. OC was added to the fecal slurry inside a selected GENbag at either of two time points, 10 min before and 1.5 h after the oxygen level reached <0.1% per the manufacturer specifications. Media extract contained OC and/or its conversion products (from biotransformation or degradation, or a combination thereof), along with other fecal and fermentation media components. To clean the extract for analysis, wet-packed SP70 resin adsorbent beads were used to selectively entrap OC and conversion products. We tracked the depletion of OC by monitoring its characteristic aldehyde proton signals (H-1 and H-3) at δ 9.22 and 9.64, respectively, using ^1^H NMR analysis in CDCl_3_. This method indicated that 3.3 mM of OC could be completely depleted and/or metabolized by mouse fecal GM within 10 min to 3.5 h, depending on the fermentation media and mouse type.

**Figure 3:**
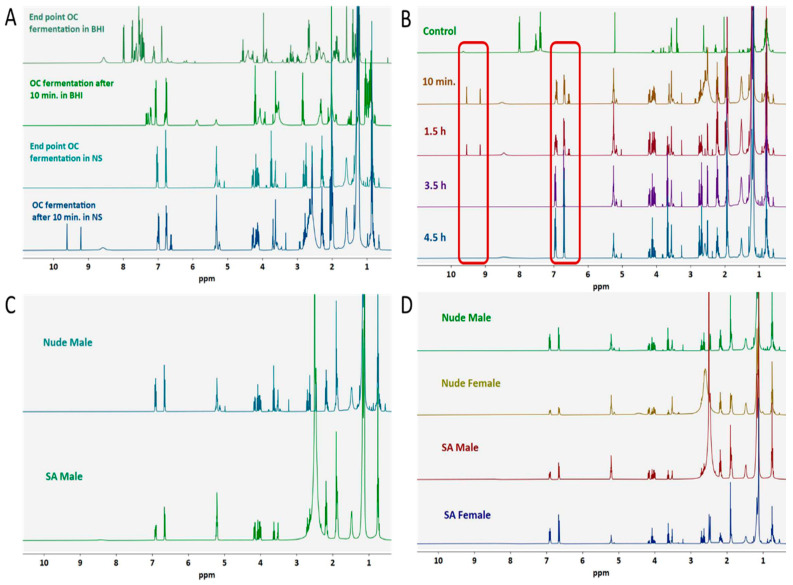
^1^H NMR (400 MHz, in CDCl_3_)-guided monitoring of OC depletion based on tracking of the OC *C*-1 aldehyde proton at δ 9.22 and *C*-3 aldehyde proton at δ 9.63. Optimization variables included anaerobic fermentation media and time, and mouse species and gender. (**A**) Fermentation media optimization. Fermentation of 10 mg OC per 250 mg fresh feces by using BHI broth versus NS to maintain the GM growth under anaerobic condition inside the GENbag with an OC addition at either of the two-time points, before and after the fermentation bag reached the optimal O_2_ level of <0.1% in 1.5 h, occurred. BHI accelerated OC-GM interaction in <10 min, resulting in a limited time frame for OC conversion products identification. (**B**) Fermentation time optimization for optimal OC conversion in NS. Ten milligrams of OC per 250 mg of fresh feces were incubated in NS under progressive anaerobic conditions at 0.17, 1.5, 3.5, 4.5 h. The end point for GM to completely deplete OC in NS was 3.5 h, the ideal time point for maximal OC conversion products. The top row control represents the fermentation mixture without OC; Red frame: ^1^H NMR signal changes for the characteristic OC functional groups over the fermentation experiments; (**C**) identification of OC main biotransformation changes in male Swiss albino and nude mice (varied species background). An ^1^H NMR spectrum of collected fermentation mixture of 10 mg of OC per 250 mg of fresh feces in normal saline under anaerobic conditions was performed for 3.5 h. Feces were collected from nude mice (immunosuppressed, *n* = 10) and Swiss albino (immunocompetent, *n* = 9). No significant differences were observed between these two samples. (**D**) Comparison of ^1^H NMR spectra of OC conversion experiments for both nude (*n* = 10) and Swiss albino (*n* = 9) mice genders. No significant qualitative variations were observed between mouse species and gender (10 mg of OC/250 mg of fresh feces in normal saline under anaerobic conditions for 3.5 h).

The ex vivo model successfully resulted in complete OC depletion after 10 min in BHI, yet some limitations became apparent that mandated modifications. In the BHI medium, OC depletion accelerated over the fermentation time-course due to proliferation of the fecal microbiota. Thus, isotonic saline (pH 5.5–6.5, sterile prior to addition to fecal samples) was used instead of BHI to maintain the appropriate viability environment yet control the undesired rapid GM growth. Thus, NS served as a viable alternative for BHI media. Unlike in BHI broth, fermentation from 10 min (zero point) to 3 h was not sufficient for GM to completely deplete OC (Figure 3A). However, by 3.5 h after reconstitution in NS, the H-1 and H-3 aldehyde signals of OC were both completely absent in the NMR readout, with a concomitant strong reduction in background signals from fecal debris. Moreover, ^1^H NMR-guided monitoring of the OC transformation pattern in NS was significantly less complicated than that observed in the BHI (Figure 3B). All groups of fecal samples from mice of both nude and SA species, and of both genders for each species, were able to completely deplete OC by the 3.5 h time-point, with similar major ^1^H NMR profile changes. These results suggested that in common GM speciation across all groups drove OC metabolic depletion (Figure 3C,D).

**Scheme 1:**
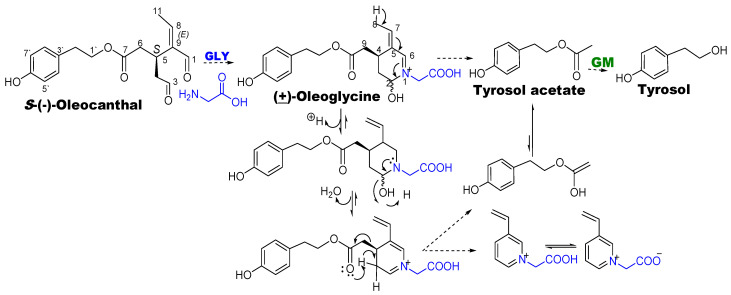
Overview of OC spontaneous interactions and metabolic transformation by GM.

In prior studies, it was seen that OC reacted spontaneously with amino acids, notably glycine, resulting in a cyclic Schiff’s base tetrahydropyridinium adduct (dubbed (±)-oleoglycine), which subsequently transformed to tyrosol acetate (Scheme 1) [8]. In our studies, OC incubation with fecal matter also resulted in the formation of (±)-oleoglycine and tyrosol acetate, the latter of which was then hydrolytically biotransformed by GM to tyrosol (Scheme 1). A plausible mechanism for spontaneous transformation of (±)-oleoglycine to tyrosol acetate, involving C−C bond cleavage, is through initial loss of a proton from the (±)-oleoglycine *C*-8 methyl, followed by subsequent carbinolamine-to-imine dehydration (loss of *C*-2 secondary hydroxyl); neighboring-group-assisted proton abstraction from *C*-3 to achieve energetically favorable full aromatization of the dihydropyridine ring could then occur with concomitant *C*-4-*C*-9 carbon-carbon cleavage to produce tyrosol acetate (Scheme 1). 

### 3.3. Microbial Taxonomy Profiling Using Shallow Shotgun Metagenomic Sequencing

**Figure 4:**
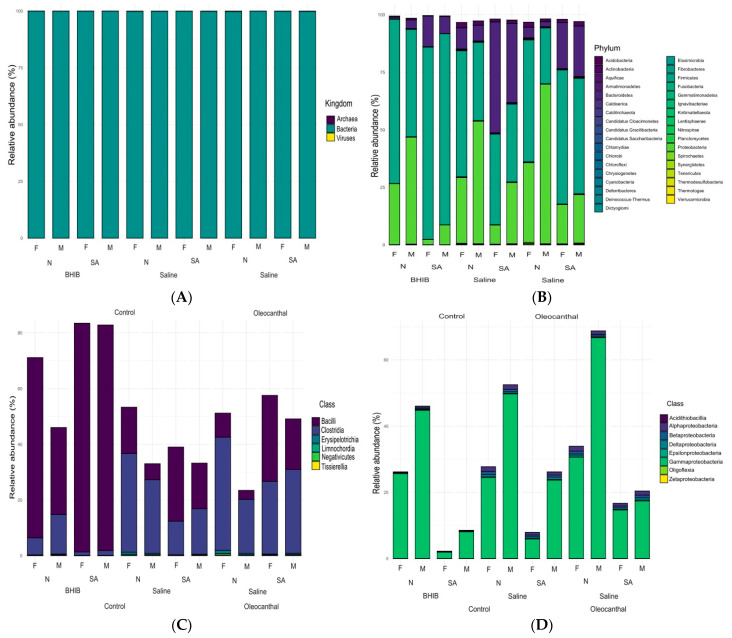
Comparison of the metagenomic profiles of taxonomic composition using shotgun sequencing of the GM. Individual vertical bars represent different samples of OC versus control in BHI and NS medium. (**A**) Composition at microbiota kingdom level; (**B**) composition at the bacterial phyla level; (**C**) bacterial classes profile for the highly prominent Firmicutes phylum; (**D**) bacterial classes profile for the highly prominent Proteobacteria phylum. N: nude mice; SA: Swiss albino mice.

To profile the fecal GM taxonomy, we used NS as a maintenance medium after controlling for mouse species and sex variants. Shotgun sequences were taxonomically classified using the Kraken2 algorithm with a pre-built MiniKraken v1 database focused on the fecal microbiome. Among the bacterial kingdom, Bacteroidetes, Firmicutes, and Proteobacteria dominated more than 99% of the phyla distribution in all samples (Figure 4A,B). Although no alteration occurred in microbiome diversity, a slight change in the phylum richness resulted in the reduction of Firmicutes abundancy, which was likely attributed to enrichment of the Bacteroidetes and Proteobacteria within the OC-treated groups compared to the control (Figure 4B). Further, changes were also seen in the classes represented within each phylum (Figure 4C,D); for example, in the Firmicutes phyla, Bacilli and Clostridia classes traded abundancies within OC-treated samples (Figure 4C), the former increasing and the latter decreasing.

### 3.4. Absolute Microbial Abundances by qPCR

**Figure 5:**
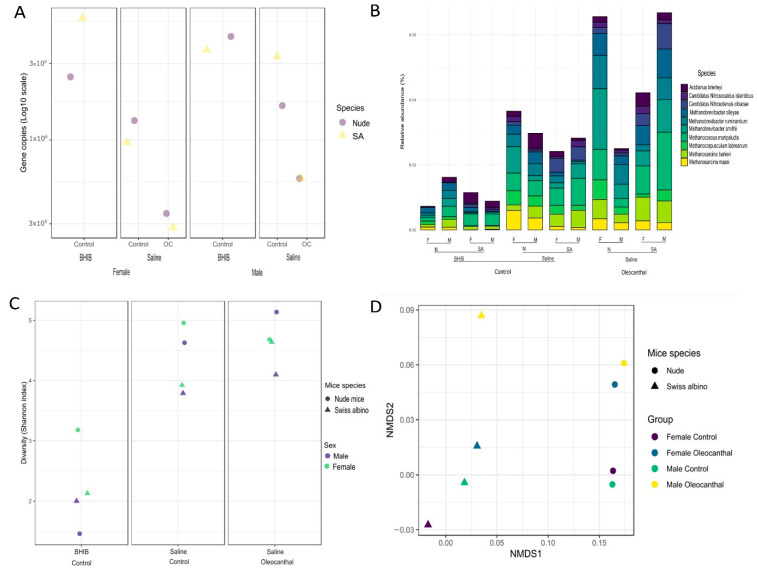
Comparison of metagenomic GM distribution data for OC versus both BHI and NS fermentation media controls in male (M) and female (F) nude (N) and Swiss albino (SA) mice. (**A**) Quantification of the changes in metagenomic read counts in assessing absolute abundance by qPCR determinations of the number of genome DNA equivalents per sample. (**B**) Gut bacteria global composition changes in the ten topmost abundant bacterial species. (**C**) Comparison of the phylogenetic (alpha)-diversity variations of OC-treated versus vehicle control-treated fecal samples based on taxonomic profiles using Shannon’s diversity index with Kruskal–Wallis chi-squared test. (**D**) Comparison of the GM beta-diversity from taxonomic profiles at the species-level using Bray–Curtis dissimilarities and samples visualized using non-metric multidimensional scaling (NMDS).

Study of the microbiome community modulation by OC between mouse type and gender under the same fermentation variables can offer better understanding of the biological health outcomes of the OC-microbiota interplay. In this study, mouse types with different microbiome backgrounds (nude and SA), genders (male and female), and growth media (BHI broth and NS) were the main factors investigated of ex vivo fecal samples under OC treatment. A molecular-level detailing and quantification method (qPCR) has been used to characterize total microbial abundancies in evaluating the use of different growth media in association with OC treatment (Figure 5A).

Perhaps most strikingly at the bacterial species-level, differences in taxonomical richness became more evident and pronounced in OC-treated versus control-treated samples among different mouse types. The five topmost-abundant species included *Muribaculum intestinale*, *Lactobacillus reuteri*, *Lactobacillus murinus*, *Lactobacillus johnsonii*, and *Bacteroides caecimuris* (Figure 5B). 

As expected, BHI broth provided better conditions for faster microbiome growth compared to NS. Meanwhile, OC-treated samples maintained in NS had dramatically reduced absolute microbiota abundance in both mouse types and genders (Figure 5). 

### 3.5. Microbial Diversities and Variation Analysis

The effect of OC on GM taxonomy at the species level was characterized by using alpha and beta diversities for binning all of the genome data obtained from the shotgun metagenomics protocol (Figure 5). The Shannon Diversity Index was used to assess both observed richness and dominance or evenness of certain bacterial species abundances. Indicators of alpha diversity per the Shannon Index of the GM in NS were compared to those in BHI both for mouse species and gender. No significant differences among these groups were observed upon OC treatment within the same variables, based on Kruskal–Wallis chi-squared = 14.963, df = 1, *p* = 0.0001 (Figure 5C). To measure the similarity/variation of microbial communities across different groups, beta diversity was compared using Bray–Curtis dissimilarities, visualized with a non-metric multidimensional scaling (NMDS) schema (Figure 5D). Based on an NMDS1 viewpoint, fecal samples obtained from nude mice were significantly separated from SA without any discernible effect of gender difference. Meanwhile, Bray–-Curtis clustering of the fecal samples at the NMDS2 level showed slight clusters separation between OC treatment and vehicle control, when controlled for the other pertinent factors. Further, findings were corroborated with Permutational Multivariate Analysis Variation (PERMANOVA) to estimate the effect of ex vivo fermentation factors on the GM taxonomic profile. Although no significant effects of OC exposure on the taxonomic profile at the bacterial phyla and class levels were identified after controlling for mouse species and sex differences, a narrow range of differences could be discerned at the bacterial species level that were clearly dominated by diversities, per the PERMANOVA analysis. We deemed these variations as prospectively key to meaningfully categorizing the bacterial species affected and unaffected by OC treatment.

### 3.6. Analysis of Differential Abundance and Functions of OC-Affected GM

**Figure 6:**
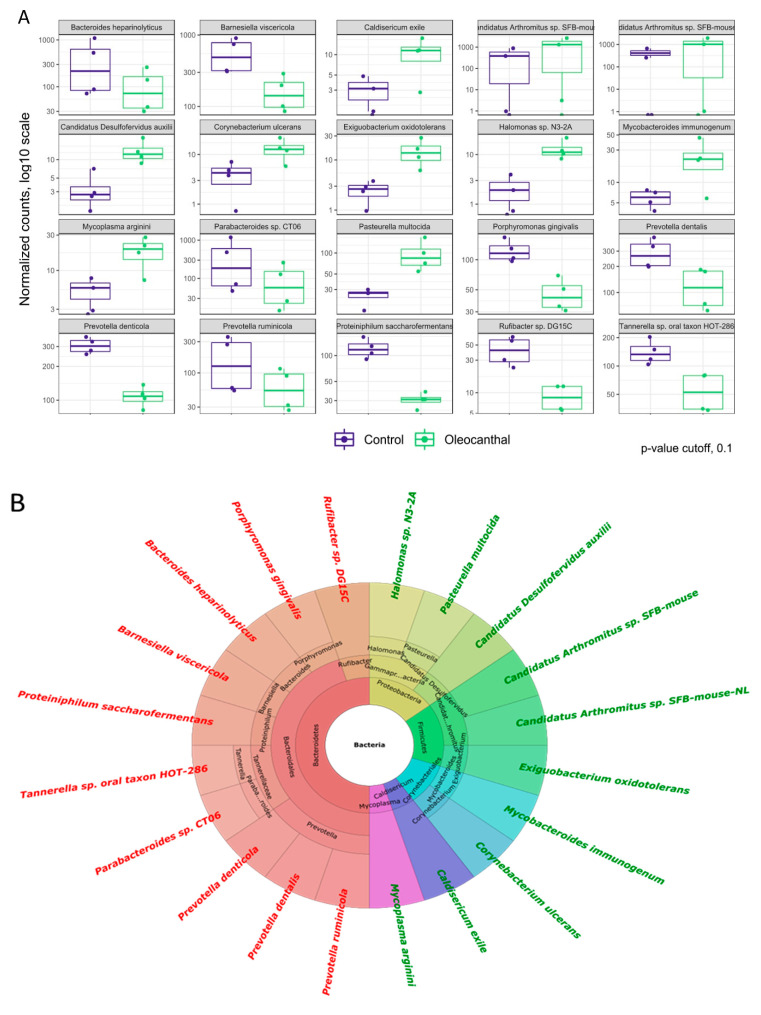

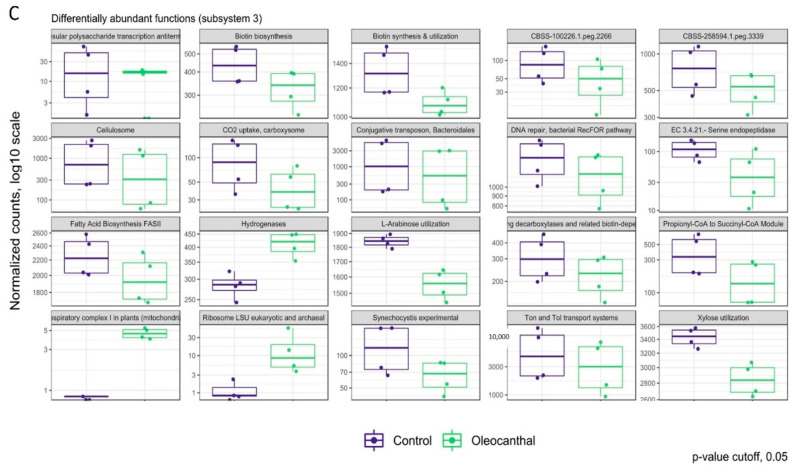
Differential abundance testing of bacterial taxonomy. (**A**) The ten topmost upregulated and downregulated taxa by OC treatments identified by differential abundance analysis using negative binomial models (DESEq2 R package) within the shotgun data after controlling for mouse species and sex differences. The analysis was obtained for NS media samples only; *p*-value cutoff = 0.1. (**B**) The linear models of operational taxonomic units, obtained using the DESEq2 R package, profiling the topmost OC-affected taxa at the species level as compared to the pertinent control in NS medium. Bacteria downregulated by OC treatments are displayed in red, while upregulated bacteria are displayed in green. (**C**) Differential abundance testing of subsystem level 3 functions. The most-regulated plausible physiological functions for GM species affected by OC were identified by differential abundance analysis using negative binomial models (DESEq2 R package) within the shotgun data, after controlling for mouse species and sex differences. The analysis was obtained for NS media only. *p*-Value cutoff = 0.05.

We hypothesized that the overlap of the minor variations among all samples within the established ex vivo fermentation model would aid in the preliminary knowledge and understanding of OC-affected GM changes in humans. To profile the topmost bacteria affected by OC within different mouse type and treatment groups, negative binomial models (DESEq2 R package) generalized from linear models were constructed to provide a picture of differential abundance by taxonomic profile (Figure 6A). Fifty-six bacterial species were seen to be suppressed, for 50 of which taxa the calculated *p*-value ≤ 0.1. OC increased the abundance of 28 bacterial species, 18 of which taxa had *p*-values ≤ 0.1 upon controlling for mouse species and gender within the NS media samples. The *p*-values were calculated with Wald tests for control groups, and with LRT tests for treatment groups, based on which the following species identified as the most abundant bacteria in the fecal samples markedly altered by OC treatment (Figure 6B): *p*-value ≤ 0.1, *Muribaculum intestinale*: base mean = 8607; log two-fold change = −1.83 (downregulated; *p*-value = 4.98 × 10^−7^; *Bacteroides caecimuris*: base mean = 6880; log two-fold change = −1.58 (downregulated); *p*-value = 0.000165; *Candidatus Arthromitus sp. SFB-mouse-NL*: base mean = 482; log two-fold change = +1.78 (upregulated); *p*-value = 1.01 × 10^−5^; *Monoglobus pectinilyticus*: base mean = 71; log two-fold change = +0.64 (upregulated); *p*-value = 0.085. The most-affected GM species by OC treatment were: *Barnesiella viscericola*: base mean = 368; log two-fold change = −1.75 (downregulated); *p*-value = 4.07 × 10^−13^; *Proteiniphilum saccharofermentans*: base mean = 76; log two-fold change = −1.94 (downregulated); *p*-value = 1.03 × 10^−12^; *Pasteurella multocida*: base mean = 61; log two-fold change = +1.94 (upregulated); *p*-value = 1.75 × 10^−11^. 

To identify specific biological functions that are characteristically affected by OC treatment, we applied negative binomial models (DESEq2 R package) to calculate the Differential Abundance Analysis distribution of genomic functions at the SEED subsystem level 3 (Figure 6C).

We observed the downregulation of more than 23 general functions and the upregulation of four general functions in response to OC treatments after controlling for mouse species and gender (NS samples only). The biological functions seen to be most affected by OC treatments were: conjugative transposon, *Bacteroidales* (base mean = 2204; downregulated; *p*-value = 4.88 × 10^−13^), propionyl-CoA to succinyl-CoA module (base mean = 291; downregulated; *p*-value = 4.09 × 10^−18^), hydrogenases (base mean = 347; upregulated; *p*-value = 0.011), and large ribosomal subunit (LSU) eukaryotic and archaeal functions (base mean = 10; upregulated; *p*-value = 0.003) (Figure 6C). These *p*-values were calculated with Wald tests for control groups and LRT testing for treatments comparisons. 

### 3.7. Association of the OC-Affected Bacteria with the Human Body

**Figure 7:**
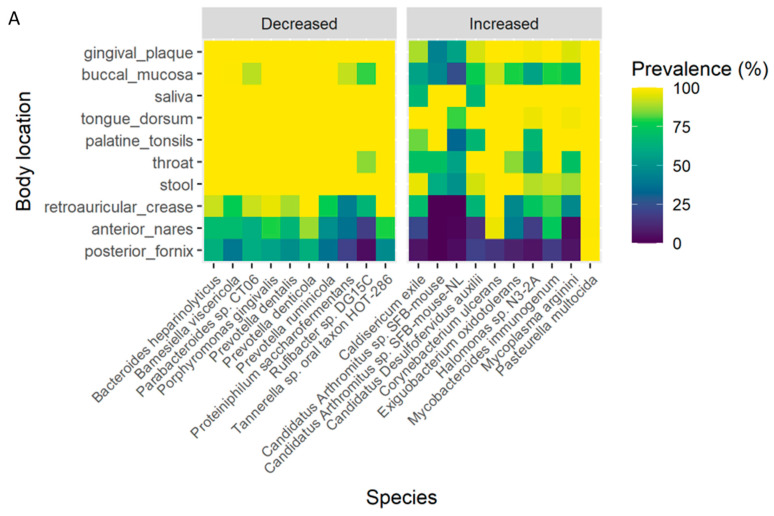

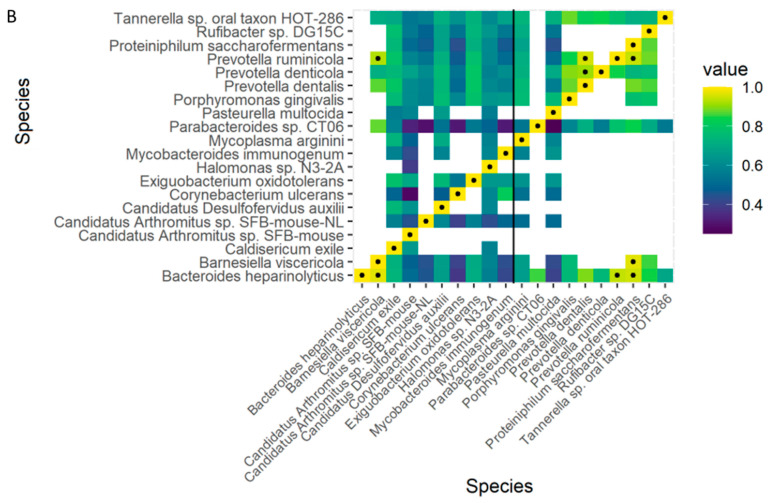
Correlation of the distribution of OC-affected mouse GM species and known human levels at various anatomical locations, as per the Human Microbiome Project (HMP) database. (**A**) Distribution of GM bacteria species most-affected by OC treatment (our experiments) in different human body locations, using a tool for comparison to HMP data. Prevalence (presence/absence) as well as mean abundances were calculated for the taxa with stratification by body location. Our data were compared against data from the HMP, which contains 690 samples collected from different human body sites and that passed quality control, each sample consisting of many varieties of bacteria. The analysis was obtained for the bacterial species most significantly impacted by OC in our experiments (*p*-value ≤ 0.1, vide supra). (**B**) Comparison by Spearman’s correlation for the OC-affected bacteria to the five taxa in the HMP database selected using *z*-score normalized abundances to validate similarities. Correlation coefficients with an absolute value > 0.9 are labeled with a black dot.

As already noted, gained knowledge and better understanding of the modulatory effects of OC on GM can prospectively help to inform of the positive health effects of OC in humans in ways that might guide its optimal nutraceutical use. However, some microbial species or cocktails existing in different animal species do not present in humans. Thus, metagenomic data comparisons may serve as a valid tool towards translating animal data to human applications. A subset of the topmost organisms seen to be altered by OC in our experiments were thus compared with the human microbiome project knowledge base, drawing on 690 human samples collected from different human body sites to see if we can find same bacteria in humans or not (https://www.hmpdacc.org/hmp/HMASM/#data, accessed on 15 May 2021). To wit, we analyzed the prevalence (presence/absence) as well as mean abundances of the top ten taxa classifications, identified based on significant *p*-values ≤ 0.1 (vide supra), seen to be upregulated or downregulated by OC treatment (Figure 7A and Tables S1 and S2). Bacterial species were stratified according to known or predicted locational prevalence within the human body, including: gingival plaque, buccal mucosa, saliva, tongue dorsum, palatine tonsils, throat, and stool. Samples with a low replication count in the used pool were discarded. Based on the heatmap ordination, all the downregulated detected species (reduced in their growth) are known to be distributed broadly in the targeted healthy human sites, with more than 96.1–99% prevalence, and 69.9–99.2% average abundance, in contrast to the constrained set of upregulated species, with 68.5–97.4% prevalence but only 0.2–30.1% average abundance. Each human physiological site is reported (per the database) to be predominantly occupied by a single species, or a particular cocktail of bacterial taxa, setting it apart from other sites. For example, *Tannerella sp. HOT-286* was predominant in the gingival plaque, buccal mucosa, and saliva samples (average abundance 48.5, 29.2, and 19.6%, respectively); *Prevotella denticola* was abundant in tongue dorsum, palatine tonsils, and throat (average abundance 36.6, 45.3, and 45%, respectively); while *Parabacteroides* sp. *CT06* was more common in stool (57% average abundance). 

To understand the correlation network for the bacterial species most impacted by OC treatment, we used the Spearman’s rank correlation coefficient for the discrepant taxonomy, carried out with the R software suite (see Methods) using *z*-score normalized abundances from the HMP database (Figure 7B). Correlations with an absolute value greater than 0.9 are labeled with black dots (Figure 7B). The correlation matrix clearly revealed variations in the abundance of OC-impacted bacteria by comparison to the natural abundance within five normal taxa in the HMP dataset (Table S2). 

## 4. Discussion

The studies we report herein explored the modulatory interactions of OC on and with GM of mice through use of an ex vivo fermentation model. OC is a unique bioactive monophenolic, dialdehydic secoiridoid occurring naturally in EVOO, with well-documented multiple beneficial activities, and as such, is a prospective high-value nutraceutical, but one warranting further understanding given the relatively narrow therapeutic index observed in animal studies, and presumably intended long-term use. The likely great significance of OC-GM interplay largely remains to be explored. Here, our ex vivo fermentation model, in conjunction with shallow shotgun metagenomic sequencing, characterized the most affected GM species in response to OC treatment as well as their distribution in the human body, with predictions of plausible impact on human biological functions. 

Treating the fecal slurry with a test xenobiotic such as OC required accurate analytical method monitoring of biotransformation changes over at least two time points past the initial time point. 

To achieve a successful and valid ex vivo fermentation study, once accurate identification of the xenobiotic metabolites with appropriate analytical method is established, time-course monitoring should record the metabolism plateau and/or complete depletion of the substrate xenobiotic. After identification of important metabolites or conversion products, there should be diversified fecal samples from different mice species with different GM communities tested, and optimal fermentation times set. Feces from each gender of mice from species-of-interest can then be compared for possible contribution of sex variation. Finally, genomic sequencing of GM species should enable identification of possible impacts of the xenobiotic under evaluation on GM makeup. Appreciation of the prospective significances of such changes have been rapidly increasing in recent years.

Fecal fermentation media and time were comprehensively optimized, and applied for studies of mouse type and gender, iteratively. Under anaerobic conditions, NS proved to be an optimal medium for conserving enough GM viability and variability to enable study of OC modulatory effects, whereas BHI medium caused uncontrolled and aggressive GM proliferation, imposing prohibitive and confounding complexities on the studies. A low-interference alternative to BHI as fermentation medium was clearly needed, given that we found detection of altered biotransformation patterns to be severely hindered by inseparable interferences from the complex array of BHI constituents. 

The OC aldehydic peaks at δ 9.22 (H-1) and δ 9.63 (H-3) served for tracking the complete depletion of OC in the NS medium over a time course between 0.17–4.5 h in nude and SA mice of both genders. Discernible initial pattern changes commenced after 10 min, while intact OC aldehydic peaked, despite declining by time, traced for over 1.5 h. As already noted, rapid OC depletion was expected due to the high reactivity intrinsic to the OC aldehydes. At 3.5 and 4.5 h of incubation, OC key aldehydic proton signals were absent, with a subsequent increase in the (±)-oleoglycine proton signals [8], followed by proton signals consistent with tyrosol acetate, which further hydrolyzed to tyrosol (Scheme 1). Although both species of mice examined here have different GM background, due in substantial measure to the immunodeficiency of the nude mice species/strain, we even so observed essentially the same pattern of OC modification in both mouse species and each of their genders. These results indicate that fecal solutions may be rich in glycine, which initiates the spontaneous conversion of OC to oleoglycine and subsequently to tyrosol acetate. It is likely that shared common GM bacterial species in all groups can comparably transform tyrosol acetate to tyrosol under these fermentation conditions. Accordingly, after 3.5 h of fermentation, OC was almost completely depleted, converted mainly by reaction with available glycine in the media to oleoglycine (cf. Scheme 1) [8]. However, this fate was not the sole one observed; several other conversion intermediates and products were concomitantly produced, varying somewhat by different fermentation batch. The lack of certain definitive confirmatory spectral and experimental evidence precluded the unambiguous structure elucidation of these products as of this time. Interestingly, though, incubation of OC in germ-free (sterilized) feces under the same fermentation conditions failed to demonstrate the same pattern of conversion products, and these results were a strong indicator of the prominent fecal ingredients-microbial contributions to the OC transformations in the ex vivo experiments, and by logical extension, presumably also in the animal and human gut.

Diet and its bioactive natural constituents play critical roles in maintaining and modulating GM homeostasis [32,33]. Our studies reported herein begin to shed some prospectively important light on the influence of OC consumption on the GM community, and likely, conversely (that is, based on these results, the GM makeup may play significant roles in the efficacy and safety of OC consumption regimes in a given individual). To better understand the effects of OC and this interplay, we used qPCR and shallow shotgun analysis to characterize and detail the aggregate microbial biomass and makeup (speciation). Our results confirm the validity of NS as a valid medium for maintaining viable core speciation of the GM community in the ex vivo fecal samples while averting the excessive growth seen with BHI, a test medium in prevalent use. Moreover, once validated, using the simpler NS as the incubation medium successfully led to the identification of key microorganisms altered by OC. Furthermore, we observed higher relative GM diversity in all OC-treated and non-treated groups in NS comparable to those observed for the BHI (the prevalent standard GM growth medium), providing powerful evidence for the ability of NS to maintain viable GM after challenging with OC treatments. These results are also promising because, as noted above, use of NS is also expected to considerably facilitate our further work on identifying OC gut transformation routes and products, and the physiological and health significances thereof.

In the OC treatment studies, although the amount of GM population decreased by 2× after several hours of treatment, no significant changes in the diversity of the major core bacterial phyla or species were seen. Additionally, only slight disparities in GM diversity were in seen for fecal samples collected from mice of each gender. The comparison of minor differences between various mouse species and genders by alpha and beta diversities and PERMNOVA analyses demonstrated the robustness of the ex vivo fermentation approach taken in this study to identify the gut bacteria most affected by OC treatment. The shallow shotgun data identified the OC-affected organisms by overlapping all tiny shifts at the bacterial species levels between the control and OC-treated samples in NS. 

As already noted, differential abundance analysis with a negative binomial distribution model revealed more than 50 bacterial species notably suppressed by OC in all samples, and 28 bacterial species markedly increased. The abundance of Bacteroidia, specially *Bacteroides caecimuris*, has reportedly been observed in the GM of severe type-2 diabetes patients, causing clear dysfunction in attributable metabolic pathways [34]. *B*. *caecimuris* has been identified as one of the major bacterial species notably abundant in the gut of non-alcoholic fatty liver disease (NAFLD) patients, in whom direct or indirect associations were also seen between gut levels of this bacterium and various inflammation and pro-inflammatory markers [35]. Intriguingly, this species was among the most-suppressed in the OC-treated groups of mice in our studies, suggesting that one primary benefit of OC consumption could be the restoration of a healthy GM profile.

Another beneficial gut bacterium with known associations to human health seen to be modulated by OC in our studies is *Monoglobus pectinilyticus*, a member of the Ruminococcaceae family [36]. This anaerobic bacterium is abundant in the human colon mucosa, and is known for its unique capability of pectin fermentation using its own pectate/pectin lyases and pectin esterases that cleave specific pectin glycosidic linkages. *M. pectinilyticus* offers a major polysaccharide source for humans, whereas pectins are otherwise indigestible by human enzymes [36,37]. 

On the other hand, and even though *Prevotella* species have been beneficially linked with the breakdown of the polysaccharides in healthy GM, some studies have correlated pronounced *Prevotella* species levels with diabetes, obesity and skin infections [38]. In our studies, three purportedly harmless strains of *Prevotella* (*denticola*, *ruminicola*, and *dentalis*) were seen to be suppressed by OC treatment. Notably and importantly, OC reduced the level of *Prevotella denticola*, which could be of prospective therapeutic value in cases of life-threatening human infections with *P. denticola* resistant to penicillins and metronidazole [39].

Collectively, the relative proportion of potentially beneficial bacterial species was increased, in ways predicted to induce remarkable changes in 27 biological functions (*p*-value ≤ 0.5). Bacterial fragment analyses identified *Bacteroidales* conjugative transposon as the topmost function anticipated to be suppressed by OC treatments. However, OC treatment enhanced the opportunistic phylum *Bacteroidetes*. *Bacteroidales* conjugative transposon is an integrative and conjugative (self-transmissible) element that encodes the gene products necessary for conjugative transfer of antibiotic resistance genes [40]. Evidently, the conjugative transposon plays a major role in spreading antibiotic resistance genes among pathogenic *Bacteroidetes* species through the silencing of T6SS loci and preventing dimerization in the host cells, wherein drug-resistant bacterial species conjugative transposons rely on [41,42]. Thus, suppression of the *Bacteroidales* conjugative transposon element by OC treatments may be fundamental in reducing antibiotic resistance gene transfer and prevalence among Gram-positive and Gram-negative anaerobes. Furthermore, OC presence in the incubated fecal samples dramatically increased species predicted to participate in hydrogen (H_2_) metabolism through upregulation of their innate hydrogenases. Hydrogenases are widely known metalloenzymes that in general catalyze the oxidation of molecular hydrogen to create protons [43]. In fact, hydrogenases catalyze the reversible oxidation of dissolved free diatomic hydrogen as energy source for bacterial proliferation [44,45,46]. 

We can readily anticipate that OC-induced changes in the taxonomy and functions of GM would affect the identities and patterns of OC conversion products (vide supra). We thus plan for future work focused on decisive and more-detailed characterization of OC gut biotransformation and conversion, and the impacts of GM changes thereupon. One of the major challenges facing the study of GM is variability, as well as disparities between the composition of human and animal GM that might also influence use of the results of OC animal studies, perhaps strongly, and especially when oral or intraperitoneal dosing is involved [47]. Hence, we carried out the metagenomics data generation and analyses, seeking to connect changes in the abundancies profile of the OC-affected bacteria in mouse fecal samples to those known to be prevalent in multiple sites of the gastrointestinal tract and elsewhere in the human body, and to predict (at this stage) possible OC-microbiota interplay therein, and prospective impacts thereof. Our comparisons for the topmost GM species upregulated and downregulated by OC with the shotgun microbiome profile of the human microbiome project (HMP) database under normal environmental conditions reveal that the majority of downregulated bacteria are widely distributed in customary human body sites, whereas the species upregulated by OC in our fecal fermentation studies exhibit a much more limited distribution across human body sites (Figure 7 and Tables S1 and S2). Results demonstrated that there are significant prospective impacts based on the observed correlation network between the GM species impacted by OC treatment and their natural abundance in the human body, especially correlations pertaining to bacteria known to be extant with relatively high abundance in humans [48,49,50]. 

One of the central major challenges for OC on the road for FDA approval as a drug entity is the need to define its fate following oral administration in humans, and, as it has become increasingly understood and appreciated, the interplay of OC with gut GM, given many of the known beneficial effects may accrue to direct or indirect effects of OC on the GM or the broader human microbiome (and in elevated doses or circumstances of aberrant GM physiological states, this may also be true with respect to possible safety issues). Thus, developing a novel ex vivo fecal fermentation model, notably the validation of an incubation medium of lesser complexity or confounding complications (NS) for studying the OC-GM, offers an exemplary approach that can potentially be extended to investigations of other bioactive natural products and xenobiotics.

## 5. Conclusions

Our studies provide for a novel and valid ex vivo GM fermentation model, in this case for studying OC, based on iterative optimization of fermentation medium and incubation time, studied in the context of mouse type and gender. Further, the generated results have provided detailed profiling of OC impacts on the GM, also paving the way for future needed and important studies. The shallow shotgun metagenomic sequences of mouse fecal samples, once subjected to further sophisticated analyses, could be used to predict the plausible distribution of affected bacteria within the normal (or pathologically disturbed) human body microbiome, also providing an initial assessment of limitations based on degree of similarity of the human microbiome to GM from two substantially disparate mouse species, with gender as a further consideration. OC modulated the growth of some GM species under anaerobic fermentation conditions that mimic the human gut environment. Furthermore, OC modulated the levels of numerous bacterial species known to critically affect various biological functions pertinent to human host health. GM modulation by OC promises beneficial impacts on human biosystems. Our studies also provide additional endorsement for OC as a potential health-promoting nutraceutical with great promise. However, further knowledge and understanding are needed to maximally capture these benefits, and to do so safely.

## Data Availability

All publicly used database listed in the article text and/or in references section.

## Supplementary Materials

The following supporting information can be downloaded at: https://www.mdpi.com/article/10.3390/nu15030618/s1, Table S1: List of 690 samples that passed quality control and were collected from 15–18 body sites from 300 healthy human subjects. Table S2: Dataset with the topmost OC-affected bacteria (increased and decreased) showing their distribution in human organs compared with the same classification database (MiniKraken) of 690 samples that passed quality control from the Human Microbiome Project.
