# Supplementary material for: S-(−)-Oleocanthal Ex Vivo Modulatory Effects on Gut Microbiota
Source: Nutrients. 2023 Jan 25;15(3):618. doi: 10.3390/nu15030618 (PMC9920009; doi:10.3390/nu15030618)
Supplement: Supplementary file 1 [file nutrients-15-00618-s001.zip › nutrients-2128393-supplementary.pdf]

Table S1. List of 690 samples that passed quality control and were collected from 15-18 body sites from 300 healthy human subjects. Data is freely accessed at the Human Microbiome Project (https://www.hmpdacc.org/hmp/HMASM/#data).

SRS ID	Body Site	Reads file location	Reads MD5	Reads File Size	Assembly file location	Assembly MD5	Assembly File Size
SRS011263	anterior_nares	/data/Illumina/anterior_	30784966e7148842	30630950	/data/HMASM/PGAs/anter	28f220153952e35ee3c547	16143
SRS046973	anterior_nares	/data/Illumina/anterior_	8e27282b91e9c81a	54134873	/data/HMASM/PGAs/anter	0d286e235bf5df37f786cd3	89164
SRS019016	right_retroauricular_crease	/data/Illumina/right_retr	17880e9921e458e5	785264307	/data/HMASM/PGAs/right_	a641d49a001438f75b7ea3	6929220
SRS013476	stool	/data/Illumina/stool/SRS	1e329e601122ddbe	7099578024	/data/HMASM/PGAs/stool	024b0aefe3825a350ea47b	50916895
SRS017307	stool	/data/Illumina/stool/SRS	96875f354287c2a3c	8772213654	/data/HMASM/PGAs/stool	abda0e92ba4ba7ae7bff92b	41582725
SRS022602	supragingival_plaque	/data/Illumina/supraging	c4e0167a8924688cc	4611691377	/data/HMASM/PGAs/supr	09b5de3ed3e1d52b21d58f	47510562
SRS024567	anterior_nares	/data/Illumina/anterior_	9eccc489c9f8f98170	25654232	/data/HMASM/PGAs/anter	3b3ddd1099df06395410a7	13607
SRS016297	buccal_mucosa	/data/Illumina/buccal_m	7abbffe4a136575da	204867797	/data/HMASM/PGAs/bucc	9c71663a0e3dd04cc46b26	2114177
SRS050007	buccal_mucosa	/data/Illumina/buccal_m	c6b5fbb727816e1d3	103287267	/data/HMASM/PGAs/bucc	c68fe24dc8a71fe132759a9	208887
SRS024310	posterior_fornix	/data/Illumina/posterior_	ecd22406fd8fe38b9	1202964236	/data/HMASM/PGAs/poste	d2ad646ae232168f9865b2	666910
SRS020233	stool	/data/Illumina/stool/SRS	8de02cfa26aafc461	9248915984	/data/HMASM/PGAs/stool	4c1049ff11f8c233c7401dd	65230043
SRS011343	supragingival_plaque	/data/Illumina/supraging	1c41d41e83358956	3058388804	/data/HMASM/PGAs/supr	93c46c45955cbba3a64308	27651764
SRS058808	supragingival_plaque	/data/Illumina/supraging	58416da1d0acf1ce0	3847756529	/data/HMASM/PGAs/supr	ad10fab7d88ab8c8255d5b	39650255
SRS063603	supragingival_plaque	/data/Illumina/supraging	50913049047b0cb7	3954360016	/data/HMASM/PGAs/supr	0ecbf5aef0599293038ebdd	48808198
SRS021960	supragingival_plaque	/data/Illumina/supraging	93b1c4f7390565147	1870703799	/data/HMASM/PGAs/supr	cc765254b6321aeb14f93a	17738246
SRS018975	supragingival_plaque	/data/Illumina/supraging	b817bb508760711c	3683092424	/data/HMASM/PGAs/supr	c315146734dc9831c5839d	26844034
SRS019968	stool	/data/Illumina/stool/SRS	36ed996c820593df0	7486543018	/data/HMASM/PGAs/stool	8f2f8ee774c627468d8dcbd	26817803
SRS045197	supragingival_plaque	/data/Illumina/supraging	3f2f1b31bfcf9c5671	5401221509	/data/HMASM/PGAs/supr	b7708a203a9d6eaa139dd8	40952283
SRS014689	throat	/data/Illumina/throat/SR	fd6b84c170ca20795	1470752520	/data/HMASM/PGAs/thro	7c01367cedd147e1a96efc4	17776402
SRS019591	supragingival_plaque	/data/Illumina/supraging	eb601f98d0fa7671a	4517740643	/data/HMASM/PGAs/supr	cc6c614e334ff4aae10f9b4f	20109886
SRS049389	tongue_dorsum	/data/Illumina/tongue_d	126fb0dc258de2c4a	7190856905	/data/HMASM/PGAs/tong	af5b0a2c27ff5d8e008734a	35911295
SRS043803	buccal_mucosa	/data/Illumina/buccal_m	597fc85cd445e4da4	371211739	/data/HMASM/PGAs/bucc	235561543874d6ab4c9917	1657831
SRS057205	tongue_dorsum	/data/Illumina/tongue_d	a15e5dfd98e98852d	5811917656	/data/HMASM/PGAs/tong	a1dae51d7b1a6f8d4b94a0	37718781
SRS014494	posterior_fornix	/data/Illumina/posterior_	93c9ca6a177a4875d	76611569	/data/HMASM/PGAs/poste	32fe0ecb1b4f0e40636b7dd	799910
SRS063417	posterior_fornix	/data/Illumina/posterior_	e2f835c31afbce877	16646879	/data/HMASM/PGAs/poste	500726828e26a074fc017a	457664
SRS017451	anterior_nares	/data/Illumina/anterior_	9fa59ed275cf19d36	78565926	/data/HMASM/PGAs/anter	0d9f410914a9c978400298	463653
SRS015640	anterior_nares	/data/Illumina/anterior_	595baf36d8b3dcdd2	18415433	/data/HMASM/PGAs/anter	6a352b0685ec954535dee0	53695
SRS057022	buccal_mucosa	/data/Illumina/buccal_m	f5191810aabdb854e	1060322977	/data/HMASM/PGAs/bucc	88d169e2a7984ffeacb4692	9389846
SRS014575	buccal_mucosa	/data/Illumina/buccal_m	174718398d6d5408	223042185	/data/HMASM/PGAs/bucc	73347383962c91d75c9d6d	1774572
SRS015895	buccal_mucosa	/data/Illumina/buccal_m	666488fdaf3681682	539236940	/data/HMASM/PGAs/bucc	a0e81ee7943830798ed60d	4884320
SRS015921	buccal_mucosa	/data/Illumina/buccal_m	6bbd3e78dac88e6b	506019241	/data/HMASM/PGAs/bucc	f4c52c4f9bb20087fb6fd58c	5646825
SRS049237	posterior_fornix	/data/Illumina/posterior_	ccc3c6e7f83956f052	73388503	/data/HMASM/PGAs/poste	d2f55542db94da3754ff574	1156068
SRS013876	anterior_nares	/data/Illumina/anterior_	153c8fe0950c40c08	177409068	/data/HMASM/PGAs/anter	9944ccebf6d1f2ba5f667ff1	2644606
SRS013723	supragingival_plaque	/data/Illumina/supraging	2334ccba328e3446c	6483909948	/data/HMASM/PGAs/supr	54021f80d9a1de5dcc3f760	43258761
SRS058186	buccal_mucosa	/data/Illumina/buccal_m	acab47d32953619df	223358291	/data/HMASM/PGAs/bucc	a3b906a2c0ba17d8649aee	3086273
SRS018394	supragingival_plaque	/data/Illumina/supraging	c5f6af4dfed91db5fc	5876822191	/data/HMASM/PGAs/supr	c71b5edb4821f1d9ef9cecd	33084574
SRS058770	stool	/data/Illumina/stool/SRS	e28502fa559e6be96	8950995228	/data/HMASM/PGAs/stool	81b7f741a32233bab19bb3	37379471
SRS019906	supragingival_plaque	/data/Illumina/supraging	8f9285704d8c74e7a	3158474539	/data/HMASM/PGAs/supr	5672e72cf2a12437206adc	21556885
SRS017080	buccal_mucosa	/data/Illumina/buccal_m	638ae91f6df1359f1	730883727	/data/HMASM/PGAs/bucc	1abd7e7f759e1e5755bcc4	6801380
SRS014343	posterior_fornix	/data/Illumina/posterior_	aee321dd03000bc9	708231930	/data/HMASM/PGAs/poste	379c2dd38d3b02344fc862	883683
SRS022725	supragingival_plaque	/data/Illumina/supraging	b079a84c2fa661f61	7414658019	/data/HMASM/PGAs/supr	754fe75f3b17c3598e8e266	49354474
SRS015215	supragingival_plaque	/data/Illumina/supraging	1e694c2740a5b771	3578258197	/data/HMASM/PGAs/supr	8218c6e2007b42e4524892	34220742
SRS022092	posterior_fornix	/data/Illumina/posterior_	a46f9db845d7440f6	56121339	/data/HMASM/PGAs/poste	b2506a780544e58f58f8511	503899
SRS051244	supragingival_plaque	/data/Illumina/supraging	b8fe494e70ec46a07	2959204322	/data/HMASM/PGAs/supr	07da04d4700d143571a846	28375013
SRS016501	tongue_dorsum	/data/Illumina/tongue_d	cdcd4e1f064093fcb	5142755184	/data/HMASM/PGAs/tong	542e9bcf8dc414735dd9eca	10945414
SRS052620	posterior_fornix	/data/Illumina/posterior_	eac01361bfe56fa31	65642454	/data/HMASM/PGAs/poste	d84b6d70b0966d74c18b0c	1213108
SRS014124	tongue_dorsum	/data/Illumina/tongue_d	c134e6f79e7f78c17	6751777967	/data/HMASM/PGAs/tong	11b28880a75f15b8c67f131	21735480
SRS014271	tongue_dorsum	/data/Illumina/tongue_d	2941691b455f4017	6005221232	/data/HMASM/PGAs/tong	d3fd7af7b14777613b8394	36905043
SRS013215	stool	/data/Illumina/stool/SRS	8a5f71b63232adf7c	7825547813	/data/HMASM/PGAs/stool	29b47f544663f98fc7cc992	9996505

SRS042910	tongue_dorsum	/data/Illumina/tongue_d	2b4499f48fcad8cad	7155326670	/data/HMASM/PGAs/tongu	a643623827e5191476a719	17681978
SRS013542	posterior_fornix	/data/Illumina/posterior_	9e78cc15b2540746f	210996818	/data/HMASM/PGAs/poste	f24d413991920338c31ce9	3231098
SRS052697	stool	/data/Illumina/stool/SRS	7eefa0912f517ef3b1	5892963376	/data/HMASM/PGAs/stool_	703097fa767ed5306aa198	59741294
SRS023583	stool	/data/Illumina/stool/SRS	50ee906bd3eb711c	6677970091	/data/HMASM/PGAs/stool_	9633d42e4c9199afc5f9c49	27613096
SRS062752	vaginal_introitus	/data/Illumina/vaginal_ir	126170cc9ada0f37b	279519831	/data/HMASM/PGAs/vagin	aaa534f4da82bd38bce60b	766490
SRS056259	stool	/data/Illumina/stool/SRS	1d1d33dc7b1aef950	8090302900	/data/HMASM/PGAs/stool_	e4bdac5fd0d7fe1fb973fff1	41765389
SRS024087	supragingival_plaque	/data/Illumina/supraging	5127d88ec3102359	4864157768	/data/HMASM/PGAs/supra	fd5669b41e4bde5c761a0f3	50566704
SRS042984	supragingival_plaque	/data/Illumina/supraging	bfc411d415a8bca16	6486020556	/data/HMASM/PGAs/supra	ec670aed38c807b8f68b1a6	38671004
SRS016037	tongue_dorsum	/data/Illumina/tongue_d	a6fa78a0d1cbb8c73	6871345745	/data/HMASM/PGAs/tongu	145e1036ce1eeb7b83c8a3	26197579
SRS024281	buccal_mucosa	/data/Illumina/buccal_m	21b53def156d3c989	2382737068	/data/HMASM/PGAs/bucca	8714a5222066cc61576c4e	4233815
SRS011086	tongue_dorsum	/data/Illumina/tongue_d	7977372b5507f2521	6074258190	/data/HMASM/PGAs/tongu	5da620fdee44b5410361e7	16755617
SRS022149	supragingival_plaque	/data/Illumina/supraging	f674f3a771fc93e9d6	1969288071	/data/HMASM/PGAs/supra	38e106ea85821097e99aad	24298877
SRS015057	tongue_dorsum	/data/Illumina/tongue_d	f23abd473ec173224	7101267405	/data/HMASM/PGAs/tongu	5b569e6d7b891026ac28e9	32026222
SRS015537	tongue_dorsum	/data/Illumina/tongue_d	9b4cb32ddc35babd	4732236726	/data/HMASM/PGAs/tongu	2b5cbf090581c25e75a4f2b	11720309
SRS011269	posterior_fornix	/data/Illumina/posterior_	e7d80f24568cb6961	387030460	/data/HMASM/PGAs/poste	0f9a54d454917d87f8a8e63	1545935
SRS020263	right_retroauricular_crease	/data/Illumina/right_retr	b11f106d34e623c0e	4532718346	/data/HMASM/PGAs/right_	e9cf718cfa5ff94542360f5e	6055489
SRS024655	right_retroauricular_crease	/data/Illumina/right_retr	b86727154990539e	770084635	/data/HMASM/PGAs/right_	3e9ba9eb07324af248c739f	1755980
SRS057083	right_retroauricular_crease	/data/Illumina/right_retr	6e43d367e231f446a	449760024	/data/HMASM/PGAs/right_	5d4dddddf298e7bc3499ce	819099
SRS021483	anterior_nares	/data/Illumina/anterior_	73e6f0cf582ac3d0bf	40815560	/data/HMASM/PGAs/anter	67bf7428aaa34cf9cf4b793f	139649
SRS014459	stool	/data/Illumina/stool/SRS	be206b81370ebeb8	5414215806	/data/HMASM/PGAs/stool_	2ffbe13743578e8c409507a	56289998
SRS054061	anterior_nares	/data/Illumina/anterior_	e0ff183c71025268a	138711242	/data/HMASM/PGAs/anter	8026bba2c90c9f4057c269c	1784810
SRS016203	stool	/data/Illumina/stool/SRS	2333ea5706de8b2c	1798861134	/data/HMASM/PGAs/stool_	f811b4568c990375dd51d9	24254774
SRS020328	stool	/data/Illumina/stool/SRS	54363635a712598d	7042085471	/data/HMASM/PGAs/stool_	65267e036d19c198df7748	53545856
SRS022609	stool	/data/Illumina/stool/SRS	ca48a5818f94a37d6	8369599616	/data/HMASM/PGAs/stool_	020cdc6cd4a8993a35ffc6a	63058723
SRS022532	buccal_mucosa	/data/Illumina/buccal_m	ebad3a9d8e582ad6	444067967	/data/HMASM/PGAs/bucca	d752454bfc5913592da695	4356596
SRS019033	right_retroauricular_crease	/data/Illumina/right_retr	eedd1c1025db27cc3	189774723	/data/HMASM/PGAs/right_	6e951b858dfb7b6bbe01b3	3263193
SRS024641	buccal_mucosa	/data/Illumina/buccal_m	c5de8062c5a9161b	40142960	/data/HMASM/PGAs/bucca	0fc08e72b413b0db3c5574f	5055
SRS063985	stool	/data/Illumina/stool/SRS	555fcb2cf2bfcefdf48	6926974329	/data/HMASM/PGAs/stool_	9e5d4549e5e5ea8b4e0afa	39164620
SRS019161	stool	/data/Illumina/stool/SRS	fd73eeedc68a3688f	6531717600	/data/HMASM/PGAs/stool_	7d7465cecfda2e73e703bb	57141477
SRS014477	subgingival_plaque	/data/Illumina/subgingiv	70224e789b976a5c	782338546	/data/HMASM/PGAs/subgi	5a5fa89c2b81f19f423124d	11749940
SRS024355	supragingival_plaque	/data/Illumina/supraging	11a4f66420644f19d	7470118362	/data/HMASM/PGAs/supra	e71f816f4dd0cc1f8381f5ce	40465972
SRS014475	throat	/data/Illumina/throat/SR	dd85ff79c0d7e6710	99527955	/data/HMASM/PGAs/throa	d583b65aa731de05df6eb0	1124281
SRS011239	stool	/data/Illumina/stool/SRS	db23cae902ca6fbed	9533267191	/data/HMASM/PGAs/stool_	15b254b028fd0704cd4e3b	40279637
SRS018656	stool	/data/Illumina/stool/SRS	81a214876d84a8f75	3160629157	/data/HMASM/PGAs/stool_	bb0d5134510131d6612029	20884623
SRS022621	tongue_dorsum	/data/Illumina/tongue_d	bedaf53a077b62301	8080121263	/data/HMASM/PGAs/tongu	8f474862504a0303a23613	33495011
SRS021473	buccal_mucosa	/data/Illumina/buccal_m	5d8fda2604c33f8f7a	700055329	/data/HMASM/PGAs/bucca	ec1d4eeb584a6c8138da35	3663697
SRS024265	stool	/data/Illumina/stool/SRS	b97321a49c7e4a0a	5833134537	/data/HMASM/PGAs/stool_	703f17ff409800d3f45a3b7	22581364
SRS053603	tongue_dorsum	/data/Illumina/tongue_d	cb86a415ea9c8ae86	5002600622	/data/HMASM/PGAs/tongu	dce04fc6160928d132592c	36706479
SRS054687	tongue_dorsum	/data/Illumina/tongue_d	e457523d157db3ad	5386561226	/data/HMASM/PGAs/tongu	a8a9a8509247f39ef367259	21498038
SRS015065	stool	/data/Illumina/stool/SRS	cddfd0595244de2b1	6165877439	/data/HMASM/PGAs/stool_	74ca4008d53f0e26daf9750	55227994
SRS063215	subgingival_plaque	/data/Illumina/subgingiv	957c4c8e33a39057d	1740933476	/data/HMASM/PGAs/subgi	1912dadb38894a75ef3a8a	20759064
SRS011152	supragingival_plaque	/data/Illumina/supraging	f7be177a323787e46	4856543235	/data/HMASM/PGAs/supra	bf258b6a3ecffcd83c24244	30887441
SRS011098	supragingival_plaque	/data/Illumina/supraging	a4871ffaea2c743d9	3776882724	/data/HMASM/PGAs/supra	08cc3e705fb38d7e7f89caf1	20700399
SRS015574	supragingival_plaque	/data/Illumina/supraging	7cba350bc9f51a3a3	3950520104	/data/HMASM/PGAs/supra	a2c9fd679e0b2f412729a71	39043971
SRS015803	supragingival_plaque	/data/Illumina/supraging	f87e0202336e50943	4271211788	/data/HMASM/PGAs/supra	f445604df1d54c1b958c55f	26087611
SRS016575	supragingival_plaque	/data/Illumina/supraging	1dc871e14e7db86d	4682422197	/data/HMASM/PGAs/supra	522c3c25fa4ca5ea1b8b3f8	43519184
SRS017511	supragingival_plaque	/data/Illumina/supraging	2e0dbfb2fddd0d93e	4969853414	/data/HMASM/PGAs/supra	bd5754a80aa9eb1a97c223	38685350
SRS055401	supragingival_plaque	/data/Illumina/supraging	756c314b770b663c	2208415867	/data/HMASM/PGAs/supra	f1d1a3131b0a35f2edc1e02	21192900
SRS015038	tongue_dorsum	/data/Illumina/tongue_d	c6adc2faaed4a3aeb	2869480332	/data/HMASM/PGAs/tongu	ff74575a82b283c83695c0d	23693729
SRS016740	tongue_dorsum	/data/Illumina/tongue_d	7671c5bcbcc376dfd	3495379991	/data/HMASM/PGAs/tongu	c7d256be631f10cea0d6f39	28316552

SRS024375	tongue_dorsum	/data/Illumina/tongue_d	d443ce0677babecee	6025968451	/data/HMASM/PGAs/tongu	e59df018030b9b1542307	20114108
SRS050244	tongue_dorsum	/data/Illumina/tongue_d	60d9e5f613b829b7f	7586847483	/data/HMASM/PGAs/tongu	4d9b1fedd82b356ffddcf69	45472083
SRS023617	tongue_dorsum	/data/Illumina/tongue_d	bf6b679f66215c67c	7734608011	/data/HMASM/PGAs/tongu	f25fc709567fd450dbce658	30570041
SRS042131	tongue_dorsum	/data/Illumina/tongue_d	853ab5fd73822894	5856439617	/data/HMASM/PGAs/tongu	30250802eecab1f2454793a	30773214
SRS016033	anterior_nares	/data/Illumina/anterior_	fd9a7d1556834b1c1	25555143	/data/HMASM/PGAs/anter	536b9c0cfe63c38e40ca116	16824
SRS019024	buccal_mucosa	/data/Illumina/buccal_m	788ed7d032ee4336	224976290	/data/HMASM/PGAs/bucca	ae5bc80ceb0d0b0c1f4eb2f	2692728
SRS019391	buccal_mucosa	/data/Illumina/buccal_m	499429546ed42ffa6	263891253	/data/HMASM/PGAs/bucca	23ccb0d2815300c5470faea	1779014
SRS062427	stool	/data/Illumina/stool/SRS	c248e43408196237	6327674999	/data/HMASM/PGAs/stool_	4aec88cb4f90c4cb0dfdbca	19998455
SRS024441	tongue_dorsum	/data/Illumina/tongue_d	7e6534dd6df1fe255	7161584189	/data/HMASM/PGAs/tongu	100fd81605c0bdfa60e3656	34120216
SRS043772	supragingival_plaque	/data/Illumina/supraging	6d68d979022ab15a	2247428689	/data/HMASM/PGAs/supra	e321fca760c09f2cefc79d54	18563937
SRS020856	tongue_dorsum	/data/Illumina/tongue_d	c72680570e67ddda	6548591340	/data/HMASM/PGAs/tongu	e7493e3b7b59b175bdfeba	25383866
SRS063932	tongue_dorsum	/data/Illumina/tongue_d	9a4360a9bebc3f0d7	6739000374	/data/HMASM/PGAs/tongu	24f31ee148da81ac9af3410	53628234
SRS015893	tongue_dorsum	/data/Illumina/tongue_d	0783704f13a3fa892	5122237358	/data/HMASM/PGAs/tongu	eaf269cb6d6845b576de54	29231862
SRS019327	tongue_dorsum	/data/Illumina/tongue_d	e69590f1cb68cacc3	5457787288	/data/HMASM/PGAs/tongu	dfc591de7d4e1fe9244a069	34699121
SRS022719	tongue_dorsum	/data/Illumina/tongue_d	f284df8c8dd05ece0	6184193442	/data/HMASM/PGAs/tongu	1ab4c0f5a2b97c0626a5a75	25472420
SRS019872	buccal_mucosa	/data/Illumina/buccal_m	2fb9c3977fc54a00b	207953983	/data/HMASM/PGAs/bucca	ed140a46e1637c21624ff4d	1780090
SRS023837	buccal_mucosa	/data/Illumina/buccal_m	f734f72f6757c962aa	284779410	/data/HMASM/PGAs/bucca	9b1fe64e12379496f853aa3	1210238
SRS016335	stool	/data/Illumina/stool/SRS	476929bdb845bb0c	8510080354	/data/HMASM/PGAs/stool_	efd3f74aebe2d12e3ca44cd	53138251
SRS016944	left_retroauricular_crease	/data/Illumina/left_retro	225af4d878d9a3c8d	270781891	/data/HMASM/PGAs/left_	755479118b8a1a535f1a44	1397965
SRS015854	stool	/data/Illumina/stool/SRS	bf6fe806870e4ebb8	6920158103	/data/HMASM/PGAs/stool_	94c6e042db6f3877a4a479	34117480
SRS053214	stool	/data/Illumina/stool/SRS	9251b6f99948ab015	7101066291	/data/HMASM/PGAs/stool_	28ec655bcd373898c8247fc	35059395
SRS017227	supragingival_plaque	/data/Illumina/supraging	1ac0044b0d54834b	6503833244	/data/HMASM/PGAs/supra	9d62bc9f21a41fac8caeba3	60750431
SRS018778	supragingival_plaque	/data/Illumina/supraging	a8398471415aa938	1489086412	/data/HMASM/PGAs/supra	81ee9842e9d5680c4d6b6f	6220098
SRS015378	supragingival_plaque	/data/Illumina/supraging	2997e84f30a80fa02	1791342187	/data/HMASM/PGAs/supra	8037294b7c7e0e0db63b4b	10044668
SRS049318	supragingival_plaque	/data/Illumina/supraging	999642297314ed05	5509103050	/data/HMASM/PGAs/supra	3ae0d61eb4d8a150a463b5	59063872
SRS015996	anterior_nares	/data/Illumina/anterior_	a70c227876fe5955f	365463394	/data/HMASM/PGAs/anter	80901b633f8131eaec7f9ea	3120347
SRS017244	anterior_nares	/data/Illumina/anterior_	b7c2dec67738f317c	24687838	/data/HMASM/PGAs/anter	560a9b0b50ef3be3cdb7a2	21797
SRS016105	anterior_nares	/data/Illumina/anterior_	ebca47d19f3afb2e7	72239353	/data/HMASM/PGAs/anter	abe4593942ae71a0cedc22	383045
SRS023930	buccal_mucosa	/data/Illumina/buccal_m	3c6d608c77567cb7e	6268974470	/data/HMASM/PGAs/bucca	4aee49c8e5a517e5a24789	7581022
SRS063272	buccal_mucosa	/data/Illumina/buccal_m	f2d6a1e84230294eb	480903764	/data/HMASM/PGAs/bucca	52c88f8d1adc81111a9765a	6815940
SRS019119	anterior_nares	/data/Illumina/anterior_	eb1e7ee0774e4249	51365975	/data/HMASM/PGAs/anter	4ed1f15a788722a338e9f3c	234316
SRS019379	posterior_fornix	/data/Illumina/posterior_	a8a4a9b8be916d97	49641194	/data/HMASM/PGAs/poste	744d7afa0871854902a529	547758
SRS017215	buccal_mucosa	/data/Illumina/buccal_m	f4866091343f7dafe	3120151339	/data/HMASM/PGAs/bucca	b32990c8b395d7e9c8cf90	7757406
SRS023591	buccal_mucosa	/data/Illumina/buccal_m	2e6eb66cd3e4a22fe	201531253	/data/HMASM/PGAs/bucca	97ceeefbea3b77b699edd8	748265
SRS023970	anterior_nares	/data/Illumina/anterior_	1fbadcd34f0df81bc3	38143134	/data/HMASM/PGAs/anter	907a8bfbf397eb40c03aff5f	45437
SRS045049	buccal_mucosa	/data/Illumina/buccal_m	ca73a5e8cadefaaa3	834366498	/data/HMASM/PGAs/bucca	a613805dd70b796d43e20b	8286174
SRS015061	palatine_tonsils	/data/Illumina/palatine_	8cf0c547f6113328b	272327125	/data/HMASM/PGAs/palat	dcc894d46f2cee2aae8a9ea	3708935
SRS016503	buccal_mucosa	/data/Illumina/buccal_m	762712f179c9b3776	326604838	/data/HMASM/PGAs/bucca	1447a0bab4e5c2e715f68b	3210642
SRS018329	buccal_mucosa	/data/Illumina/buccal_m	2e37722e4b2b7057	1754273457	/data/HMASM/PGAs/bucca	89fdbfef06fbd6a1e73a644	4167405
SRS047044	stool	/data/Illumina/stool/SRS	15a1f81f6756f0372	6048987584	/data/HMASM/PGAs/stool_	eb241135cf99c7d8697c560	31167815
SRS019129	subgingival_plaque	/data/Illumina/subgingiva	6cae06249b1888e3	1184759385	/data/HMASM/PGAs/subgi	c668c7c63a6858adc93cc14	19140631
SRS046623	buccal_mucosa	/data/Illumina/buccal_m	82efb6cf0b96a8e21	422161568	/data/HMASM/PGAs/bucca	e5cf16ef2379311766d8705	1888529
SRS015044	supragingival_plaque	/data/Illumina/supraging	7c7571042356b3e7	3652256371	/data/HMASM/PGAs/supra	6c9b2549606c4d47caf6e5e	30459617
SRS055118	buccal_mucosa	/data/Illumina/buccal_m	683f91778b63355fa	457236910	/data/HMASM/PGAs/bucca	c67fee89fb450a01f082aed	558066
SRS017701	stool	/data/Illumina/stool/SRS	34c0e07987281b86	7489299183	/data/HMASM/PGAs/stool_	e0be9ea755ed87ba8991fd	34339177
SRS052668	buccal_mucosa	/data/Illumina/buccal_m	b1038596904f5afb2	909310851	/data/HMASM/PGAs/bucca	7601ec2f4f2c845288e3dc6	8649983
SRS064704	posterior_fornix	/data/Illumina/posterior_	308533e2d78dd745	133326453	/data/HMASM/PGAs/poste	a0a7f8d57608410d837b7e	862859
SRS046688	right_retroauricular_crease	/data/Illumina/right_retr	0c057651f6fdc022d	1163006142	/data/HMASM/PGAs/right_	b7404bf8a258d6d5642add	1937854
SRS058182	right_retroauricular_crease	/data/Illumina/right_retr	ea61382fb1a91e000	3691217335	/data/HMASM/PGAs/right_	2d2efb8b26f69e3a3a805b5	8083372
SRS019120	saliva	/data/Illumina/saliva/SRS	c884c9135361f2ae4	567445993	/data/HMASM/PGAs/saliva	7ef63ca7c4109a0b9da2e92	6702897

SRS015430	anterior_nares	/data/Illumina/anterior_	86dbd47ab7007e65	35575892	/data/HMASM/PGAs/anter	b69833bed137f3d1328ad7	92236
SRS013711	buccal_mucosa	/data/Illumina/buccal_m	553efd9c091aca753	751634275	/data/HMASM/PGAs/bucca	b89c4d599526a6eb9b05d4	6351897
SRS018351	stool	/data/Illumina/stool/SRS	47583391fb44b8d03	7619945198	/data/HMASM/PGAs/stool_	79c869ff64c4ae035ef20ad8	32190108
SRS047210	tongue_dorsum	/data/Illumina/tongue_d	69b7044c1fa3f9c778	5947807631	/data/HMASM/PGAs/tongu	92712cfd12e268fa9248229	29483466
SRS022083	supragingival_plaque	/data/Illumina/supraging	83fb56de167b379a7	2343809812	/data/HMASM/PGAs/supra	448e217b262a70b4d394ba	17268263
SRS045313	supragingival_plaque	/data/Illumina/supraging	e510f933fda8e09d5	1238455168	/data/HMASM/PGAs/supra	6055767381f5fb7e6b0c7c8	9635019
SRS023352	tongue_dorsum	/data/Illumina/tongue_d	c55ffb39f17936d9ba	6679910716	/data/HMASM/PGAs/tongu	9026db5b0042a8c80e253a	30629463
SRS024081	tongue_dorsum	/data/Illumina/tongue_d	72fde2e9989673814	7573215102	/data/HMASM/PGAs/tongu	6638ac97fb2e8813dc1cdbf	39181883
SRS058213	anterior_nares	/data/Illumina/anterior_	8aeb6b833632607b	205940081	/data/HMASM/PGAs/anter	b019fb4f86cdc034b3c9d53	2524357
SRS024017	buccal_mucosa	/data/Illumina/buccal_m	17e033c1eca98d39d	930067873	/data/HMASM/PGAs/bucca	dcb517fefdc376489f69340	4794169
SRS016516	posterior_fornix	/data/Illumina/posterior_	64f926a939b2bb8ec	80023964	/data/HMASM/PGAs/poste	6a23479d8012613bf7938b	614696
SRS023468	posterior_fornix	/data/Illumina/posterior_	49bfd9274d9450c38	6862114031	/data/HMASM/PGAs/poste	3d8b3c5dba7d9de53453de	14854842
SRS011586	stool	/data/Illumina/stool/SRS	641094595cd6562d	8768974280	/data/HMASM/PGAs/stool_	172876af016afe489989511	43664173
SRS017433	stool	/data/Illumina/stool/SRS	248ffbd498a628f71f	7680151901	/data/HMASM/PGAs/stool_	bdaaea6f69bd06cbebc430	32845396
SRS012279	tongue_dorsum	/data/Illumina/tongue_d	c854d1dd144c2d7ce	7637343983	/data/HMASM/PGAs/tongu	946ae772b050dc3506c624	41815543
SRS016086	tongue_dorsum	/data/Illumina/tongue_d	259c519cb0757b8df	4767707191	/data/HMASM/PGAs/tongu	c48234aab924751b9224a7	20782791
SRS016188	anterior_nares	/data/Illumina/anterior_	76423400ce6ef6c2d	35536436	/data/HMASM/PGAs/anter	a7c64a826826de284b83b6	180957
SRS019125	attached_keratinized_gingiv	/data/Illumina/attached_	b3bfd385662f10c9c	4566153548	/data/HMASM/PGAs/attac	defca5aa68508f46f05ad27	13854697
SRS018569	buccal_mucosa	/data/Illumina/buccal_m	37de4216b2df4098f	146777758	/data/HMASM/PGAs/bucca	3a8c343350f38f1b77ef3e1	1229641
SRS020336	buccal_mucosa	/data/Illumina/buccal_m	23753ef8187bca368	163988459	/data/HMASM/PGAs/bucca	7fbc432ef86e7538c3352fa	553810
SRS051116	buccal_mucosa	/data/Illumina/buccal_m	daa356067ddd5d49	349640001	/data/HMASM/PGAs/bucca	401302cfce4cc0ace913359	2353531
SRS015663	stool	/data/Illumina/stool/SRS	ac731a2fc6c7813f1a	7343149791	/data/HMASM/PGAs/stool_	de6e5d2f194911e5f5dfd21	54907059
SRS019601	stool	/data/Illumina/stool/SRS	7da71fb37fd63f51f4	7732004821	/data/HMASM/PGAs/stool_	c1233678e6782d3e28b336	40813721
SRS055378	supragingival_plaque	/data/Illumina/supraging	59108bd52174969d	4381377331	/data/HMASM/PGAs/supra	4564902951eea28bfb8626	44494889
SRS019894	tongue_dorsum	/data/Illumina/tongue_d	96ab8b94903a51dd	6269259390	/data/HMASM/PGAs/tongu	85bd4910136c1aebfa1afd2	23491129
SRS044662	tongue_dorsum	/data/Illumina/tongue_d	736fe29ec7c79fdf37	4092820632	/data/HMASM/PGAs/tongu	204b1520e1d67f30ca4d9d	21121212
SRS047824	tongue_dorsum	/data/Illumina/tongue_d	3a34fe3f31466a9ca	6636476636	/data/HMASM/PGAs/tongu	3c2480263cbc804d74374d	32923595
SRS018369	anterior_nares	/data/Illumina/anterior_	f210fded219622315	25614289	/data/HMASM/PGAs/anter	57742e10ab24b7aacbe3a9	35681
SRS022137	stool	/data/Illumina/stool/SRS	72ce3c2847afe07c4	4852439595	/data/HMASM/PGAs/stool_	00ff154d53e332e2b4cc988	36220267
SRS014468	saliva	/data/Illumina/saliva/SRS	322e815c760b4c8b5	174865669	/data/HMASM/PGAs/saliva	7119b2fc56804818167c10	909834
SRS024009	stool	/data/Illumina/stool/SRS	692246b99835d570	7242857903	/data/HMASM/PGAs/stool_	99011052adfe50e6b85ce5	24290616
SRS055450	supragingival_plaque	/data/Illumina/supraging	334dadf765739d53e	2143935451	/data/HMASM/PGAs/supra	b3787078b6d6589706146a	18914929
SRS065310	supragingival_plaque	/data/Illumina/supraging	bf8f8540f184da32b	984900093	/data/HMASM/PGAs/supra	51c00c2d063ac5ddb9f87fa	13020948
SRS020226	supragingival_plaque	/data/Illumina/supraging	421eb5a9fb383f827	5830956791	/data/HMASM/PGAs/supra	4a8ead31da65b4e99e994c	39331567
SRS015578	stool	/data/Illumina/stool/SRS	96bba4e95aa27b69	7950752989	/data/HMASM/PGAs/stool_	c8b8202a384e17d876922b	44486833
SRS016056	stool	/data/Illumina/stool/SRS	ec8f31d3a192169b3	5781036758	/data/HMASM/PGAs/stool_	db63eff8dfe93ed7bd85172	20927133
SRS019087	anterior_nares	/data/Illumina/anterior_	fc6b8971e6c38991b	51585202	/data/HMASM/PGAs/anter	59f14826230e11dffd64c5c	176215
SRS019867	anterior_nares	/data/Illumina/anterior_	f5d859bcbe9ae88e9	60795841	/data/HMASM/PGAs/anter	9b8d28e288fea62eeef217b	969023
SRS051031	stool	/data/Illumina/stool/SRS	affd9f7eff0de88d3c	7846658401	/data/HMASM/PGAs/stool_	1e19d3aaef231a615c495b	56695076
SRS016111	posterior_fornix	/data/Illumina/posterior_	697001eaa1dab38d	41372844	/data/HMASM/PGAs/poste	a492390b5aeeaf8da23bab	373821
SRS015985	buccal_mucosa	/data/Illumina/buccal_m	b07b7e0298e65961	2579033527	/data/HMASM/PGAs/bucca	8c14fa3e3244d025efa6381	14741490
SRS018774	buccal_mucosa	/data/Illumina/buccal_m	6abffd5d2ad39ffefe	641949056	/data/HMASM/PGAs/bucca	187b5bb1f56dd2de429599	4077837
SRS018585	anterior_nares	/data/Illumina/anterior_	9d4129d2f5fdd51b9	17652230	/data/HMASM/PGAs/anter	44bd3fefb5e8722b3128a0	8061
SRS015470	supragingival_plaque	/data/Illumina/supraging	3f7e13c7aa4ba5d83	4818046997	/data/HMASM/PGAs/supra	6bdd63d4779f458a8662bd	26930066
SRS049744	anterior_nares	/data/Illumina/anterior_	6d9e2ffc82b08ef37	22519445	/data/HMASM/PGAs/anter	6126c99791388f4c3e27834	14609
SRS050925	stool	/data/Illumina/stool/SRS	6e64aa409899b10f2	6576976714	/data/HMASM/PGAs/stool_	9951c77eca034e7a528e65	51731258
SRS019128	supragingival_plaque	/data/Illumina/supraging	2a881c1d348d5134	4006863179	/data/HMASM/PGAs/supra	87caab0a44ae02a30ccaaf0	33978676
SRS052590	anterior_nares	/data/Illumina/anterior_	93173e6051ad9c37	219829246	/data/HMASM/PGAs/anter	6e56386917996a4c41b2f0	115703
SRS019333	supragingival_plaque	/data/Illumina/supraging	2f06c1f883be4f9ab	1654702420	/data/HMASM/PGAs/supra	4591942ba0d694e94a911f	11920385
SRS019026	palatine_tonsils	/data/Illumina/palatine_	995f97158cc50a990	1869625684	/data/HMASM/PGAs/palat	ba4f85c4043867a8ccd1d0d	23560201

SRS024289	supragingival_plaque	/data/Illumina/supraging	338028ce4e64dd9d	2710900286	/data/HMASM/PGAs/supra	6cdec210fc6b7571a1d34aa	30762659
SRS022158	posterior_fornix	/data/Illumina/posterior_	0c5e829cbd1f79451	69622218	/data/HMASM/PGAs/poste	12fc6602cec94250c5f316d	712591
SRS045978	buccal_mucosa	/data/Illumina/buccal_m	edb10556664f14571	616878910	/data/HMASM/PGAs/bucca	952d7a8fc6278da88403b7	4456626
SRS052604	supragingival_plaque	/data/Illumina/supraging	5be0f8527855b1c07	1380930063	/data/HMASM/PGAs/supra	9c636a8f690f4912252d068	15526318
SRS063999	supragingival_plaque	/data/Illumina/supraging	6979f66cfc2782fc3e	4719944144	/data/HMASM/PGAs/supra	fbf8a223c5d2367c2469bad	45283330
SRS011140	tongue_dorsum	/data/Illumina/tongue_d	7a7931f58a5bc0709	5585336024	/data/HMASM/PGAs/tongu	74d920e50396be6f1122c6	22386212
SRS011405	stool	/data/Illumina/stool/SRS	d764dd5c06ea433c4	8808562205	/data/HMASM/PGAs/stool_	74df8ba3301de24c28941b	27624484
SRS050422	stool	/data/Illumina/stool/SRS	421f0cec34c8946dd	5573582754	/data/HMASM/PGAs/stool_	54f2be69513e2dc2cf9e2c9	50657958
SRS015055	saliva	/data/Illumina/saliva/SRS	2841467ebbbff274c	597489127	/data/HMASM/PGAs/saliva	341cef4fa4572aac9e0da1c	5757231
SRS051613	anterior_nares	/data/Illumina/anterior_	bbb25b41df1a88f74	63601603	/data/HMASM/PGAs/anter	3a57134fe4df0ed21dbdfb1	196934
SRS011529	stool	/data/Illumina/stool/SRS	690aeaf18183c8004	9059911056	/data/HMASM/PGAs/stool_	8518800d8a8c269d7d541f	41338630
SRS065142	anterior_nares	/data/Illumina/anterior_	3b05d6fcb205106fb	14140647	/data/HMASM/PGAs/anter	3210d30d49e882cb251e73	7811
SRS014894	supragingival_plaque	/data/Illumina/supraging	da1aee663228bb22	2292838686	/data/HMASM/PGAs/supra	64468d58abf402e92b6057	20546758
SRS014923	stool	/data/Illumina/stool/SRS	92838f9c63ffc0f4a4	7263407700	/data/HMASM/PGAs/stool_	7042fb6272048043eb8784	55722283
SRS020862	supragingival_plaque	/data/Illumina/supraging	9044de9b99a763a3	2102844444	/data/HMASM/PGAs/supra	90a48230106e8b9aebc979	12810023
SRS013950	subgingival_plaque	/data/Illumina/subgingiva	9f66ffc4fbd1abb0e9	1907044343	/data/HMASM/PGAs/subgi	ad9eb69b2d5b7173dc6a36	24955636
SRS023835	tongue_dorsum	/data/Illumina/tongue_d	656d1eddc2ad3f000	5822499875	/data/HMASM/PGAs/tongu	cfa1ea0cfec0c5d1a64333c2	28381115
SRS015072	mid_vagina	/data/Illumina/mid_vagi	039f08ba2b6405d8c	70285643	/data/HMASM/PGAs/mid_	2fc7d2c323c7778b8b3fa2e	1056986
SRS016559	posterior_fornix	/data/Illumina/posterior_	ad768eee242c70b5	117178244	/data/HMASM/PGAs/poste	7c8562d4f751f08ff1648ec0	613305
SRS015381	right_retroauricular_crease	/data/Illumina/right_retr	d67604da7be24fe13	1125244822	/data/HMASM/PGAs/right_	22a5105d2d294e76e40e63	1862297
SRS057539	tongue_dorsum	/data/Illumina/tongue_d	04e27d65de82e0fd	6157438753	/data/HMASM/PGAs/tongu	3870b5bcd0417ce92b368b	32627153
SRS019116	right_retroauricular_crease	/data/Illumina/right_retr	2508f17cdce2290ae	493711073	/data/HMASM/PGAs/right_	b0159b23c3667309c8d364	7252688
SRS018784	anterior_nares	/data/Illumina/anterior_	e061c986c11bfc307	35267700	/data/HMASM/PGAs/anter	b4fbd25bedd357f1f0cdef4	217081
SRS065278	tongue_dorsum	/data/Illumina/tongue_d	2e997e10207890c5	5236413047	/data/HMASM/PGAs/tongu	169dbef66bc9cd3fc1a525e	31200191
SRS017191	stool	/data/Illumina/stool/SRS	50089d990bfbba374	7417389884	/data/HMASM/PGAs/stool_	72103dcae640319a22d8d3	31437083
SRS015274	buccal_mucosa	/data/Illumina/buccal_m	7c768f2a2d5e12050	211648279	/data/HMASM/PGAs/bucca	28d7810ec95ee7b8916d01	1563562
SRS015745	buccal_mucosa	/data/Illumina/buccal_m	e96b33f32ba734e0d	720502100	/data/HMASM/PGAs/bucca	210264a5f29a7e68fd5fe62	6582029
SRS045004	stool	/data/Illumina/stool/SRS	656a1140024ae0ee	8620371571	/data/HMASM/PGAs/stool_	2077c05b251bd29c42e303	33960250
SRS019329	buccal_mucosa	/data/Illumina/buccal_m	ab9ba71cd9f0564f2	566633476	/data/HMASM/PGAs/bucca	54985c776d6c0a66f0975f4	6340555
SRS015217	stool	/data/Illumina/stool/SRS	95293250d7f81404a	6471556130	/data/HMASM/PGAs/stool_	48e0331056b41a0af546e6	47811432
SRS017697	anterior_nares	/data/Illumina/anterior_	5ba8b1ef44f587caa	48153493	/data/HMASM/PGAs/anter	31da48838814d493bb1406	599185
SRS021986	buccal_mucosa	/data/Illumina/buccal_m	8e051c36b27cec20e	896937100	/data/HMASM/PGAs/bucca	dc4f500913d481d8415f4b5	2917788
SRS024549	stool	/data/Illumina/stool/SRS	2aa13abdeae695671	6857386524	/data/HMASM/PGAs/stool_	f8fa588a7c90886eca3343e	15273913
SRS043001	stool	/data/Illumina/stool/SRS	ce546d14f5f48825e	8386424977	/data/HMASM/PGAs/stool_	af48c13ca5a1048f77022fe	17667636
SRS014690	supragingival_plaque	/data/Illumina/supraging	d690eb77a5917386	3976909176	/data/HMASM/PGAs/supra	61b7aff7a506bc9b81d6e5d	19957216
SRS015440	supragingival_plaque	/data/Illumina/supraging	cd0c21f923c0a08e4	3994596274	/data/HMASM/PGAs/supra	8dd8f772cab4725b7ea6d6	26253352
SRS023358	supragingival_plaque	/data/Illumina/supraging	c0a0984de86e61082	2076612375	/data/HMASM/PGAs/supra	ebac6035b3796a73163d63	15059919
SRS019077	supragingival_plaque	/data/Illumina/supraging	df0d82ed16e4565d	3277617930	/data/HMASM/PGAs/supra	01013d631b686924603e84	26821250
SRS011243	tongue_dorsum	/data/Illumina/tongue_d	b08d4022eca8752d	4928787308	/data/HMASM/PGAs/tongu	eb8380e68e731e7570000c	16441843
SRS054430	supragingival_plaque	/data/Illumina/supraging	c4fbb40489e81f4e9	2107136363	/data/HMASM/PGAs/supra	3e5ccfd28e65c45487b7f15	18734580
SRS054653	supragingival_plaque	/data/Illumina/supraging	e26924f9eaf4668be	2896055045	/data/HMASM/PGAs/supra	34f1890f4c712dc9429912d	14564517
SRS013879	tongue_dorsum	/data/Illumina/tongue_d	30d69df4fb4b1778c	6112464420	/data/HMASM/PGAs/tongu	b47f1e4836f532a5a58718d	23153672
SRS048791	tongue_dorsum	/data/Illumina/tongue_d	7100eb98175709ed	4602010403	/data/HMASM/PGAs/tongu	87a97b99bf6f54a127a01c7	28749136
SRS045127	tongue_dorsum	/data/Illumina/tongue_d	31f6cefb7adb38b35	2405705857	/data/HMASM/PGAs/tongu	36c6b8784ace82e0a05a83	14296604
SRS052227	tongue_dorsum	/data/Illumina/tongue_d	262834c6b0fa4e02d	3166044433	/data/HMASM/PGAs/tongu	18766cc3ef731fa28c8c5f3a	15408396
SRS062540	tongue_dorsum	/data/Illumina/tongue_d	f14bbf5a01462f557	1957219605	/data/HMASM/PGAs/tongu	ababe0f2dd708743958d7f2	14376386
SRS022077	tongue_dorsum	/data/Illumina/tongue_d	140ad82614fa1be47	6503340151	/data/HMASM/PGAs/tongu	7b7336d6fe30c033fa15d61	34789097
SRS023354	buccal_mucosa	/data/Illumina/buccal_m	2dcaef6fec0e0c0494	260051081	/data/HMASM/PGAs/bucca	c79f6a31d7937911078a04	1372308
SRS016584	posterior_fornix	/data/Illumina/posterior_	d4e3ed0ed4382186	150786030	/data/HMASM/PGAs/poste	e864c07bdce4d3380ab629	994497
SRS019600	posterior_fornix	/data/Illumina/posterior_	f3be706eb7bf0a534	82607177	/data/HMASM/PGAs/poste	10aacf8fe1940b8981785f1	608750

SRS016349	buccal_mucosa	/data/Illumina/buccal_m	4ebfbed79a264eca6	360385642	/data/HMASM/PGAs/bucca	f7603b10784b4b6104b8e7	2046971
SRS023604	posterior_fornix	/data/Illumina/posterior_	7ce7bb12f6903e0ac	5111660875	/data/HMASM/PGAs/poste	5cff2b5156613f2c7bacf7af	14599576
SRS042858	posterior_fornix	/data/Illumina/posterior_	e6e9b65a1fe04da0f	226854563	/data/HMASM/PGAs/poste	9b7edd1f9695a0a3bc4203	613791
SRS019386	anterior_nares	/data/Illumina/anterior_	29502fb506bce65e2	42299969	/data/HMASM/PGAs/anter	9fdc7951cbebfb81d533017	573587
SRS043422	buccal_mucosa	/data/Illumina/buccal_m	565e3f35a3ea7336d	6435497287	/data/HMASM/PGAs/bucca	c6f4a2222f9958171beecb1	20288935
SRS018978	right_retroauricular_crease	/data/Illumina/right_retr	3a5609c68f458658b	738526173	/data/HMASM/PGAs/right_	28a94a4769b70bccfea781d	6317212
SRS044474	anterior_nares	/data/Illumina/anterior_	0b60b8702094797f	241202975	/data/HMASM/PGAs/anter	38db9289883fe43d24fed1	3316423
SRS016989	stool	/data/Illumina/stool/SRS	a7eec5eec004b27c1	9057098152	/data/HMASM/PGAs/stool_	22a7dbc786855a12c08cb1	26069392
SRS019582	stool	/data/Illumina/stool/SRS	2cd776921f36c7e0f	5599855779	/data/HMASM/PGAs/stool_	d5a7a4356c5a3df68ff4eac	49582076
SRS022071	stool	/data/Illumina/stool/SRS	d6fb120611b0cd3cc	7147617683	/data/HMASM/PGAs/stool_	db6737b189e3aaa29f2f97d	48841288
SRS057807	posterior_fornix	/data/Illumina/posterior_	efbdd32d40016b0d	412788470	/data/HMASM/PGAs/poste	c63e5bc56b720d1b01f52b	663991
SRS062520	posterior_fornix	/data/Illumina/posterior_	07c54eec215d5dbc6	60176638	/data/HMASM/PGAs/poste	aea762d4b48681f913ce0e	570309
SRS022721	buccal_mucosa	/data/Illumina/buccal_m	33ce775c1579a4b5	276573920	/data/HMASM/PGAs/bucca	0f6d92839c432f4dbd83c28	2631420
SRS024625	stool	/data/Illumina/stool/SRS	13c6e1fff2db54113	7335462419	/data/HMASM/PGAs/stool_	39a9ebe6c8f433b9b4fd07e	29680025
SRS052874	buccal_mucosa	/data/Illumina/buccal_m	cff3e743a15ff0aa88	1150556735	/data/HMASM/PGAs/bucca	f79e25b28b650da7f99c43d	6058660
SRS054569	buccal_mucosa	/data/Illumina/buccal_m	e7c63f948e3af2579	1760409564	/data/HMASM/PGAs/bucca	3cc34383b4551aff4782c83	9490731
SRS018427	stool	/data/Illumina/stool/SRS	3a09e4d580cdf5d6b	7211876581	/data/HMASM/PGAs/stool_	19695acdbec3532acb295c	35711856
SRS024132	stool	/data/Illumina/stool/SRS	98a0264de28103a8	7515233254	/data/HMASM/PGAs/stool_	aff9793fb53121c9ed5c0f0c	63223531
SRS045713	stool	/data/Illumina/stool/SRS	6b5839175e540de9	7769376975	/data/HMASM/PGAs/stool_	6b4e93e21317413eeaccac	27259539
SRS055982	stool	/data/Illumina/stool/SRS	920f4bddc3abaa26f	5960020424	/data/HMASM/PGAs/stool_	5124086d4ca7b621291181	30512371
SRS051378	supragingival_plaque	/data/Illumina/supraging	224190c5bf259e569	1559654089	/data/HMASM/PGAs/supra	70607caac07d4d2ce3c0dc4	16646733
SRS013818	tongue_dorsum	/data/Illumina/tongue_d	a7a09b59e8a66c4a	4084013557	/data/HMASM/PGAs/tongu	bc214a6ec78a4a60c255ae	21955264
SRS015797	tongue_dorsum	/data/Illumina/tongue_d	791bcf081cf590682	7059520690	/data/HMASM/PGAs/tongu	4fd1e2b3d1e7efc9b15fd46	29121065
SRS043755	supragingival_plaque	/data/Illumina/supraging	7df473a291d96c2c1	2610213020	/data/HMASM/PGAs/supra	4c2470fc5be51dc54a027fc	20030647
SRS017209	tongue_dorsum	/data/Illumina/tongue_d	a47eff41bbcfa4ece6	6074802435	/data/HMASM/PGAs/tongu	3f0550b587c5e9e0eb3822	30707215
SRS043239	buccal_mucosa	/data/Illumina/buccal_m	1e2661f6d7eb4a239	897187967	/data/HMASM/PGAs/bucca	7836ed424b2c17d20bd18c	9366203
SRS013258	left_retroauricular_crease	/data/Illumina/left_retro	a56827ec36c7f7273	1550446872	/data/HMASM/PGAs/left_	6e1622acbe89ed9bbc4224	3092776
SRS012902	stool	/data/Illumina/stool/SRS	ccb357d1fa93a8115	7253047686	/data/HMASM/PGAs/stool_	30f33f0149f370147663485	9759588
SRS015190	stool	/data/Illumina/stool/SRS	ea08286e92d9704e	9094546546	/data/HMASM/PGAs/stool_	2318d2f6010c49e41d0383	28352192
SRS050484	anterior_nares	/data/Illumina/anterior_	4bb769aab6b4e008	467527955	/data/HMASM/PGAs/anter	1854114d8723853539f174	65526
SRS017441	buccal_mucosa	/data/Illumina/buccal_m	347faf375c8243730	736462935	/data/HMASM/PGAs/bucca	fadee6b6c83ebae4007e29	2155019
SRS018359	buccal_mucosa	/data/Illumina/buccal_m	784fe32a0b0628e28	1086453343	/data/HMASM/PGAs/bucca	3b080241fac099986d7d8b	4465641
SRS011132	anterior_nares	/data/Illumina/anterior_	b6602114a83d101d	30874163	/data/HMASM/PGAs/anter	f045f8895e7c8ba62a9f473	42343
SRS018463	anterior_nares	/data/Illumina/anterior_	150a3559ceaf98dd8	35647099	/data/HMASM/PGAs/anter	e62bc474804b5064e8fcdb	194804
SRS014890	buccal_mucosa	/data/Illumina/buccal_m	16e3de6268788bd2	157995771	/data/HMASM/PGAs/bucca	b63cbacc9851ec66d8bbe4	712791
SRS015154	buccal_mucosa	/data/Illumina/buccal_m	c34d29fd263d4b35	486127009	/data/HMASM/PGAs/bucca	14742264ef130905b10efd	4967382
SRS014682	anterior_nares	/data/Illumina/anterior_	c5a22d2d16cd5d53	382236711	/data/HMASM/PGAs/anter	748e4db4f7681e0f45ef836	3098986
SRS062713	anterior_nares	/data/Illumina/anterior_	b20ab15ba8a2cd91	105015419	/data/HMASM/PGAs/anter	67ebee9a76569987270c47	1613212
SRS013239	buccal_mucosa	/data/Illumina/buccal_m	5b5cae3791f8b5bd5	896066089	/data/HMASM/PGAs/bucca	e5fd2d63713b2891ae34d7	5752402
SRS075406	buccal_mucosa	/data/Illumina/buccal_m	9a0e2eae3cc7e5f8f	698311168	/data/HMASM/PGAs/bucca	fd93174b29252856f6bcaef	5549067
SRS016517	stool	/data/Illumina/stool/SRS	0e59f2386bf8ff106f	225	/data/HMASM/PGAs/stool_	12eb35f21626f9c23019fbf	23578724
SRS065504	stool	/data/Illumina/stool/SRS	4f0413c6e979c7f52	8381979466	/data/HMASM/PGAs/stool_	c06b552b2746f8d0a59219	50677749
SRS023595	supragingival_plaque	/data/Illumina/supraging	0fe28ed433202b7fe	6912131371	/data/HMASM/PGAs/supra	58a13a6823e4553f0e83728	58936951
SRS019122	tongue_dorsum	/data/Illumina/tongue_d	fa8f0096a2dd4030c	7180433458	/data/HMASM/PGAs/tongu	8ffd293e5b1f91c530cd6fc7	25797753
SRS020334	tongue_dorsum	/data/Illumina/tongue_d	cb79c4f274b4e19f3	3762602524	/data/HMASM/PGAs/tongu	72ad8cfb37fae8e3e6ac0c5	18819257
SRS044486	tongue_dorsum	/data/Illumina/tongue_d	002fcb8a4ea6133cd	4539154013	/data/HMASM/PGAs/tongu	ce6cc983960ff28d300a97d	17808838
SRS045715	tongue_dorsum	/data/Illumina/tongue_d	a32655f43b61a1c92	7167523487	/data/HMASM/PGAs/tongu	29904717fdc049bc6bd7ed	34013973
SRS056323	tongue_dorsum	/data/Illumina/tongue_d	eb17d34d5a7f6e01f	4321880481	/data/HMASM/PGAs/tongu	5112a976db6c72360f124c	21701725
SRS016600	buccal_mucosa	/data/Illumina/buccal_m	8d2d78e858527b11	868467112	/data/HMASM/PGAs/bucca	6d74cc5d17fa310d003ade	2527662
SRS014692	saliva	/data/Illumina/saliva/SRS	9dbffa1ea3a6713db	918863743	/data/HMASM/PGAs/saliva	746daca76d88caf39079de5	12421645

SRS014613	stool	/data/Illumina/stool/SRS	cab2dc0c7fae7a219	7414916626	/data/HMASM/PGAs/stool	b06f98771da6c9c6b4ee5d1	37936415
SRS015264	stool	/data/Illumina/stool/SRS	57d9e78fb6581724	4144734815	/data/HMASM/PGAs/stool	de3c89303bd89a27dec79a	39698323
SRS019787	stool	/data/Illumina/stool/SRS	4e55def8839ec1cdb	7245914840	/data/HMASM/PGAs/stool	abc0a111f2b740b97e897d	23679684
SRS023971	stool	/data/Illumina/stool/SRS	7877d36da08c3bc9	8909817937	/data/HMASM/PGAs/stool	42c4a28e8f08fcf88c5b86f9	16259996
SRS049959	stool	/data/Illumina/stool/SRS	05c2203bc32eaf418	7896740348	/data/HMASM/PGAs/stool	934dc155b4f8ed021fdbbd4	65343291
SRS049995	stool	/data/Illumina/stool/SRS	731f003f0cab5e42ff	8728263127	/data/HMASM/PGAs/stool	1268e159350d6d41a5368d	51153863
SRS019028	supragingival_plaque	/data/Illumina/supraging	0fe5d766f53c436a9	4586058344	/data/HMASM/PGAs/supra	379ba700bb4b05dce3a664	32597981
SRS062761	tongue_dorsum	/data/Illumina/tongue_d	ad056ff1b2f7c1b22	8417525290	/data/HMASM/PGAs/tongu	1511060bcafa107ed3ee03	24104793
SRS049268	supragingival_plaque	/data/Illumina/supraging	c466bc01bfcc44e6d	3765649474	/data/HMASM/PGAs/supra	ec6802b67f370c493322df7	37182627
SRS019045	tongue_dorsum	/data/Illumina/tongue_d	5483442fc541dfc87	4892040766	/data/HMASM/PGAs/tongu	21ebfa5bf06bb1ed7e755d5	21481499
SRS022530	tongue_dorsum	/data/Illumina/tongue_d	5261e3f10953cf59c	3598556015	/data/HMASM/PGAs/tongu	a91a3ab59feb977318a693	29674745
SRS015937	anterior_nares	/data/Illumina/anterior_	da19867b56dd5d2e	202878197	/data/HMASM/PGAs/anter	0fe41a3a4930665bfc57de0	2947312
SRS024424	anterior_nares	/data/Illumina/anterior_	552427d1ac2cdbe52	310843441	/data/HMASM/PGAs/anter	7d83256c62525a75d2495b	3063757
SRS011310	buccal_mucosa	/data/Illumina/buccal_m	405c0e4bfddbb4407	1434647151	/data/HMASM/PGAs/bucc	35f650190d2c52e31a7f9a6	7608105
SRS016039	buccal_mucosa	/data/Illumina/buccal_m	13052a10599982d3	672006614	/data/HMASM/PGAs/bucc	a6b66c9fc681439fb3e5cfe	5567486
SRS077738	buccal_mucosa	/data/Illumina/buccal_m	27a41fc805b99b553	289524370	/data/HMASM/PGAs/bucc	3d15772ec044e653bc149c	3203002
SRS020349	posterior_fornix	/data/Illumina/posterior_	f2e52704cafe6d78a	200854178	/data/HMASM/PGAs/post	85ca2029ed36c52e98dad1	673186
SRS050184	posterior_fornix	/data/Illumina/posterior_	ae13118083b5fb8e1	87627427	/data/HMASM/PGAs/post	eed0521fb6e81bfa2d6e87	867919
SRS045606	right_retroauricular_crease	/data/Illumina/right_retr	6249882a442ff5406	1682424814	/data/HMASM/PGAs/right_	1cbd28422e524f9cccbb869	6907502
SRS057717	stool	/data/Illumina/stool/SRS	581b6c62ab14f675a	7019221660	/data/HMASM/PGAs/stool	87d218586250cfedbc616d	13195524
SRS043018	supragingival_plaque	/data/Illumina/supraging	533ceab3b49af8205	3575719953	/data/HMASM/PGAs/supra	bf1e758e5bead308b33d7b	31246815
SRS047113	supragingival_plaque	/data/Illumina/supraging	6c10b5ac1bfeed79a	5764834568	/data/HMASM/PGAs/supra	74f01939163577f8979497	47736766
SRS022129	anterior_nares	/data/Illumina/anterior_	99fdcc156f7e647f94	79658511	/data/HMASM/PGAs/anter	80c5a8f71d0bc97eec65a9e	557049
SRS055495	tongue_dorsum	/data/Illumina/tongue_d	b027ee03f8450788	488015643	/data/HMASM/PGAs/tongu	dc62d2d984c1366a927e89	3884277
SRS057791	tongue_dorsum	/data/Illumina/tongue_d	386a41b9db14c84e	6335922877	/data/HMASM/PGAs/tongu	0d5afe19f34b696912a99fc	31278055
SRS065431	buccal_mucosa	/data/Illumina/buccal_m	7fe2439fc70e34fc3c	39886438	/data/HMASM/PGAs/bucc	ea06e0ba3e182a5b0eb434	378733
SRS011452	stool	/data/Illumina/stool/SRS	e0f9f04c6072c7719	2440316702	/data/HMASM/PGAs/stool	5eeea2b78ffff7188f48d445	13156139
SRS015793	anterior_nares	/data/Illumina/anterior_	48b72a38332af4618	55521914	/data/HMASM/PGAs/anter	4efe66cc5bbca6cffda2f9b0	1731699
SRS017103	stool	/data/Illumina/stool/SRS	679348e08b0badae	8192112367	/data/HMASM/PGAs/stool	93f58d99044912125bce70	47139329
SRS019597	anterior_nares	/data/Illumina/anterior_	60ff893c47fc93a2af	54586552	/data/HMASM/PGAs/anter	63fcf21ed03f27285dea60f4	763697
SRS018575	stool	/data/Illumina/stool/SRS	52cc8f42addff578b9	6360611802	/data/HMASM/PGAs/stool	9204a86ec2f6e0f2b29513a	15412805
SRS024557	buccal_mucosa	/data/Illumina/buccal_m	0f6a4ffd33702d25d	275426207	/data/HMASM/PGAs/bucc	619666b022ef6abad97b2f	1902882
SRS023847	anterior_nares	/data/Illumina/anterior_	2f31a00251ad05574	484888188	/data/HMASM/PGAs/anter	745355fd8768462edcaed6	2703164
SRS050752	stool	/data/Illumina/stool/SRS	061952621c88c90b	7038559068	/data/HMASM/PGAs/stool	cf26e81b4f776dc773d285d	41086613
SRS063035	anterior_nares	/data/Illumina/anterior_	27c5b786ec26ed70	76757470	/data/HMASM/PGAs/anter	8297734c19a054c2765a91	915119
SRS045262	buccal_mucosa	/data/Illumina/buccal_m	fb5a67d924954d5c6	1269722409	/data/HMASM/PGAs/bucc	526be37778ae76c61b43c2	7388220
SRS019267	stool	/data/Illumina/stool/SRS	17aef8297fcc43eb8	8595158476	/data/HMASM/PGAs/stool	8bce18d419d43d2ec1b9d5	20266146
SRS064645	stool	/data/Illumina/stool/SRS	ce1a93a65c1596d7d	1471794270	/data/HMASM/PGAs/stool	c77e8d7a70d8c204612ebb	13034380
SRS019124	buccal_mucosa	/data/Illumina/buccal_m	9428d7706ac7af7bb	1795684950	/data/HMASM/PGAs/bucc	a538e9d4a5b8c9bfc53169f	10619596
SRS020858	buccal_mucosa	/data/Illumina/buccal_m	86a970b2c854b706	957060483	/data/HMASM/PGAs/bucc	9ab5dcf5caf6365f6cfaaa83	3958271
SRS019126	palatine_tonsils	/data/Illumina/palatine_	b8e2b71ec3cfd63ad	4513377194	/data/HMASM/PGAs/palat	7861cc469e7a60b26615f61	27101375
SRS018791	tongue_dorsum	/data/Illumina/tongue_d	49f32bada280c361d	8285648079	/data/HMASM/PGAs/tongu	8d59e0221340ebe2ef0789	26516195
SRS020869	stool	/data/Illumina/stool/SRS	c3512cc1711857ff8	7293015464	/data/HMASM/PGAs/stool	95d29c03f2ddadb69f04285	51152095
SRS013951	stool	/data/Illumina/stool/SRS	4481be537e82b431	5938989481	/data/HMASM/PGAs/stool	2a0e25e5e9be36b4a3df53	51560944
SRS050299	stool	/data/Illumina/stool/SRS	75f2d9fcdfdfd808ac	4823110306	/data/HMASM/PGAs/stool	cc0d9b509e79ef8d24a1ca4	25570750
SRS049147	tongue_dorsum	/data/Illumina/tongue_d	062681c098d9469f5	7146880245	/data/HMASM/PGAs/tongu	f69b79b3ae3f68306c0dd3b	24539753
SRS015782	stool	/data/Illumina/stool/SRS	7e55ea6164626c2b	6979382746	/data/HMASM/PGAs/stool	c2b5b47260bd875b56edf5	56557203
SRS023176	stool	/data/Illumina/stool/SRS	3715f9f15ddbe11f3	8307317996	/data/HMASM/PGAs/stool	15d5b0adb4741efcc761217	19751660
SRS024435	stool	/data/Illumina/stool/SRS	4d7681a08b7532e1	8345669788	/data/HMASM/PGAs/stool	836cf3c5770e19365b5533	59471176
SRS015059	buccal_mucosa	/data/Illumina/buccal_m	9d4dd8f13177dbf6c	71631974	/data/HMASM/PGAs/bucc	34d6b9221fea7b7b6f7598	176308

SRS052027	stool	/data/Illumina/stool/SRS	468957efc0e08f59e	8566463067	/data/HMASM/PGAs/stool	d7095b0dc8b7f5e7610bb0	31244212
SRS023841	supragingival_plaque	/data/Illumina/supraging	7a055aaa299934ac8	2159871106	/data/HMASM/PGAs/supra	f7236aa06540a4df2f6de05	24320414
SRS064557	stool	/data/Illumina/stool/SRS	9bc5ef80e9b602dce	8127610522	/data/HMASM/PGAs/stool	fff5590d748a52bfd47121d	26437395
SRS024447	supragingival_plaque	/data/Illumina/supraging	8846ed0c7ed540f07	5430502499	/data/HMASM/PGAs/supra	422f9ef6ef0f01bde680ac9	27707779
SRS015540	buccal_mucosa	/data/Illumina/buccal_m	73ff6d9a189919640	765432063	/data/HMASM/PGAs/bucc	86f5add833d3ba5f3c41db3	7875118
SRS019219	tongue_dorsum	/data/Illumina/tongue_d	3f9bec4017900fbcb	5206973451	/data/HMASM/PGAs/tong	d7d25e0120e5caaa29f3c30	32008961
SRS013234	tongue_dorsum	/data/Illumina/tongue_d	1fadcde6977d79ec6	5389142253	/data/HMASM/PGAs/tong	af1f1f24e577ce6938393bf1	16269638
SRS048719	buccal_mucosa	/data/Illumina/buccal_m	f4bbd30608d09c592	482110459	/data/HMASM/PGAs/bucc	1a37c23c3d20901b02f1551	4086660
SRS013705	tongue_dorsum	/data/Illumina/tongue_d	4590be46ffc341b74	7286921783	/data/HMASM/PGAs/tong	4205e8ff87fbc7c98b9f4880	46599290
SRS016342	tongue_dorsum	/data/Illumina/tongue_d	c8bcb7ee44ae54444	5341715792	/data/HMASM/PGAs/tong	50ef3094065fb856f262ea3	24894719
SRS015644	tongue_dorsum	/data/Illumina/tongue_d	f92b66a93c7365306	5326378549	/data/HMASM/PGAs/tong	fc0df32183f77078aa2b834	23821709
SRS015762	tongue_dorsum	/data/Illumina/tongue_d	eead6e1bc3304779	7069600296	/data/HMASM/PGAs/tong	22670f0d67880ac83cb942	42954330
SRS016002	tongue_dorsum	/data/Illumina/tongue_d	575cb350b7694e43	6585699864	/data/HMASM/PGAs/tong	199e541392d0c5fd3b92e2	32935628
SRS017520	posterior_fornix	/data/Illumina/posterior_	7006c252fa10233c5	92434230	/data/HMASM/PGAs/post	d0c285792d4756154eec77	707177
SRS024068	posterior_fornix	/data/Illumina/posterior_	4edbf627ff498bdc67	116317194	/data/HMASM/PGAs/post	cf3e363b3387d87a9760ce	960051
SRS048411	tongue_dorsum	/data/Illumina/tongue_d	5add03e3b77d0f435	6577710883	/data/HMASM/PGAs/tong	aa041a16fce0e758b6deec9	25445696
SRS024428	posterior_fornix	/data/Illumina/posterior_	54115fb50dc5945e5	120064567	/data/HMASM/PGAs/post	4764d82e38571fbc82b3c36	1041586
SRS016581	anterior_nares	/data/Illumina/anterior_	19a44dbe7057ff17e	29298648	/data/HMASM/PGAs/anter	9268fbfbc91eeeaafa860f6d	22665
SRS015071	vaginal_introitus	/data/Illumina/vaginal_ir	2372fa7816fa0e3b3	33916101	/data/HMASM/PGAs/vagin	60450dc90439f023b08deaf	675653
SRS013946	attached_keratinized_gingi	/data/Illumina/attached_	2f3eb882d63b22a16	5104144709	/data/HMASM/PGAs/attac	f502f3c3e5ec7129c95c77d	14015539
SRS019215	anterior_nares	/data/Illumina/anterior_	55de248bbfa8c1bbf	10626323	/data/HMASM/PGAs/anter	d2e2220376ad9fd3a3ba75	12361
SRS017127	buccal_mucosa	/data/Illumina/buccal_m	ccc7105414e3d9230	2220737513	/data/HMASM/PGAs/bucc	73003fb8ebcbd1a6c453391	6365559
SRS017687	buccal_mucosa	/data/Illumina/buccal_m	374a83803b64bcc7e	614938324	/data/HMASM/PGAs/bucc	b1833f1e07d1b2616c8222	5688226
SRS049900	stool	/data/Illumina/stool/SRS	325e8a07a84eeab2	9178874642	/data/HMASM/PGAs/stool	131a19cf815142b6ff64245	18158142
SRS050628	buccal_mucosa	/data/Illumina/buccal_m	7f6a3b78dcea8bf8b	1556971104	/data/HMASM/PGAs/bucc	43007059a78dd3d7f16bcf5	13070468
SRS015960	stool	/data/Illumina/stool/SRS	5e653cf5b31ec32c5	6463200259	/data/HMASM/PGAs/stool	6e2d65d0903c94c2c460e6	35110325
SRS012294	posterior_fornix	/data/Illumina/posterior_	baee3d1f829ff0e96	74283395	/data/HMASM/PGAs/post	f0633fe8902a69b804c524e	515699
SRS018969	tongue_dorsum	/data/Illumina/tongue_d	a15ae345ed5ff0caaf	3176315970	/data/HMASM/PGAs/tong	be524241b1405b5a2779e0	14647974
SRS022143	tongue_dorsum	/data/Illumina/tongue_d	bb1c76c2d1993f071	7989265389	/data/HMASM/PGAs/tong	3ceb12e7417d8619298afa	39935454
SRS024331	stool	/data/Illumina/stool/SRS	87dd5d3dc3a89fea2	7911163871	/data/HMASM/PGAs/stool	e418748779fb2e9afd0bac9	44758533
SRS054956	stool	/data/Illumina/stool/SRS	423f5b29648c5bb5d	6508180205	/data/HMASM/PGAs/stool	92e26f53e0c30c4d77aadc2	26201035
SRS058723	stool	/data/Illumina/stool/SRS	81025a313cd7d9ca	7556866011	/data/HMASM/PGAs/stool	293887215dfeac268df34d	18021914
SRS014979	stool	/data/Illumina/stool/SRS	d2f226b925026b98	6868025827	/data/HMASM/PGAs/stool	018bae609dd17f4c7979f4c	45407381
SRS021484	stool	/data/Illumina/stool/SRS	9846b52f23489493	8843012717	/data/HMASM/PGAs/stool	090cde374f812accae107d8	40661084
SRS048870	stool	/data/Illumina/stool/SRS	6f9002660318ff2a7	6860240628	/data/HMASM/PGAs/stool	a867903db27f056c3e2e99	38330812
SRS013252	supragingival_plaque	/data/Illumina/supraging	1d087f2bd09b40ee	7097122456	/data/HMASM/PGAs/supra	5e73cd2c92770caac63cd35	39692631
SRS016043	supragingival_plaque	/data/Illumina/supraging	19507605fa72d997	2550128000	/data/HMASM/PGAs/supra	6f74804bd9a2a35c73b90d	27596151
SRS016200	supragingival_plaque	/data/Illumina/supraging	8f2c94972ab91f615	4181718026	/data/HMASM/PGAs/supra	9c2b3126d5e0a6f468434c	21236348
SRS024021	supragingival_plaque	/data/Illumina/supraging	c70daa57f8b841cb7	6058508753	/data/HMASM/PGAs/supra	c6eea10151d2f87d8d0577	20925010
SRS011306	tongue_dorsum	/data/Illumina/tongue_d	9e379adff6f2ec3a04	7801267867	/data/HMASM/PGAs/tong	09fc7645a1ada9df261ec61	33475117
SRS018300	tongue_dorsum	/data/Illumina/tongue_d	e12ef1518c7a9cb45	7104481703	/data/HMASM/PGAs/tong	b202b473db355162f11618	33111414
SRS013502	tongue_dorsum	/data/Illumina/tongue_d	e7ebc1f6df3b9414a	2689117899	/data/HMASM/PGAs/tong	9e5fd1a0d177d19455186c	14960016
SRS015941	tongue_dorsum	/data/Illumina/tongue_d	1bf4c101446152c11	5941084331	/data/HMASM/PGAs/tong	8f2f6f95067fe9ccd04f758c	30802005
SRS014573	tongue_dorsum	/data/Illumina/tongue_d	797729ef9ef352857	5509097485	/data/HMASM/PGAs/tong	32e2b434e6e0f353970a49	37694855
SRS014888	tongue_dorsum	/data/Illumina/tongue_d	8be2bf0c19af36a31	5691058068	/data/HMASM/PGAs/tong	1baa9f0dc75dab0a9c9403e	21683663
SRS063193	tongue_dorsum	/data/Illumina/tongue_d	8c9054e30cdae835e	4148156790	/data/HMASM/PGAs/tong	3456efbcee41001778b864	26624778
SRS075404	tongue_dorsum	/data/Illumina/tongue_d	df4195acddc7d0e1f	9463055941	/data/HMASM/PGAs/tong	88d5adb392eacafe878edb	38297829
SRS014686	buccal_mucosa	/data/Illumina/buccal_m	6fb82bf74647224e9	438999628	/data/HMASM/PGAs/bucc	14ef847e15ee61eb68a345	3613086
SRS052988	right_retroauricular_crease	/data/Illumina/right_retr	9dfe56d9005d88efa	1117577026	/data/HMASM/PGAs/right_	de85f8eb4146ac3ea26e6f1	981819
SRS023526	stool	/data/Illumina/stool/SRS	bc27f92c16779b402	7655517585	/data/HMASM/PGAs/stool	07aaf2cb2a9f2197a48c99ff	49905735

SRS015064	subgingival_plaque	/data/Illumina/subgingiv	1cf8bd7f3cd906ab1	1675708890	/data/HMASM/PGAs/subgi	19c2f1dba3785545b1d2b2	14127622
SRS018739	tongue_dorsum	/data/Illumina/tongue_d	ae7d4c0f925e76c48	5618655987	/data/HMASM/PGAs/tongu	d870f1ebf40f9930ad7193f	41732947
SRS019071	tongue_dorsum	/data/Illumina/tongue_d	65ca02c6da95e2c75	7893442784	/data/HMASM/PGAs/tongu	09b7617666c3a27b0041c4	35047549
SRS019389	tongue_dorsum	/data/Illumina/tongue_d	cd98d71072589509	2382375220	/data/HMASM/PGAs/tongu	3106752dea0114acc26b80	14300934
SRS050669	tongue_dorsum	/data/Illumina/tongue_d	1bdac6d189f91d035	8091627924	/data/HMASM/PGAs/tongu	6dd5f0d8db39fe927bf0d6c	31653045
SRS019039	anterior_nares	/data/Illumina/anterior_	b1c5475b63bc694a1	113283010	/data/HMASM/PGAs/anter	6a435c792832d97af6175c6	1521415
SRS020222	buccal_mucosa	/data/Illumina/buccal_m	5e15b7247ba76a26	426285456	/data/HMASM/PGAs/bucca	0536695a59073f9888fbc1d	3964677
SRS019019	anterior_nares	/data/Illumina/anterior_	702dc4439b7039f21	152954704	/data/HMASM/PGAs/anter	99de96ebb930c388a9872d	1696363
SRS011584	posterior_fornix	/data/Illumina/posterior_	28fbe41f89ddc598d	28421624	/data/HMASM/PGAs/poste	ad000734baf6747cc0623e6	467495
SRS063178	anterior_nares	/data/Illumina/anterior_	336f0b31b92880224	13701163	/data/HMASM/PGAs/anter	731eda8d45f9a3c798552d	10938
SRS017537	buccal_mucosa	/data/Illumina/buccal_m	8b5a9510995631f8a	428783521	/data/HMASM/PGAs/bucca	1fc001ae94bde7ddd8a39a5	1602811
SRS012663	anterior_nares	/data/Illumina/anterior_	ce5e83e17d85cdcd9	105667144	/data/HMASM/PGAs/anter	aaa53f67dfaff5404b86d217	870145
SRS065347	posterior_fornix	/data/Illumina/posterior_	107f4a7575a8209f2	217562409	/data/HMASM/PGAs/poste	3b28e929dbfabd0b186494	1010468
SRS075419	posterior_fornix	/data/Illumina/posterior_	9c1b6f603a9b688dc	52638667	/data/HMASM/PGAs/poste	0fdf1737c829c7e755ec70d	831588
SRS016495	stool	/data/Illumina/stool/SRS	7401e1f7f47074fdd	7845447222	/data/HMASM/PGAs/stool_	f54865629d43f77e800c86b	25852709
SRS011247	buccal_mucosa	/data/Illumina/buccal_m	78a6db8899e74fc4d	1742396548	/data/HMASM/PGAs/bucca	aae168230eb2a1cb9a6341	8778372
SRS018313	stool	/data/Illumina/stool/SRS	abc7771c6eaffb59b	8544547833	/data/HMASM/PGAs/stool_	f27f5785e98f2756403c518	19722640
SRS015646	buccal_mucosa	/data/Illumina/buccal_m	6142bedc0c9b7b03	1185179767	/data/HMASM/PGAs/bucca	08f4811ab4f7b52cedb28e1	2789565
SRS017700	posterior_fornix	/data/Illumina/posterior_	f7d27d1b34fac345f4	1010113435	/data/HMASM/PGAs/poste	e722472690224a4c4b2089	682065
SRS017810	buccal_mucosa	/data/Illumina/buccal_m	1212c9980028c660	749983286	/data/HMASM/PGAs/bucca	87dce8b858b200b4b252f1	6597428
SRS019910	stool	/data/Illumina/stool/SRS	88201d804cb1c52b	4410497968	/data/HMASM/PGAs/stool_	d86eb148f90eb94ab9667d	30196696
SRS024598	right_retroauricular_crease	/data/Illumina/right_retr	983d7ab046d1345e	3183304154	/data/HMASM/PGAs/right_	077d134bd7e45f7b1ed525	3930039
SRS011134	stool	/data/Illumina/stool/SRS	c7823681575fd7198	8972422892	/data/HMASM/PGAs/stool_	27370bed4dc14ca7b9de61	61032910
SRS015755	supragingival_plaque	/data/Illumina/supraging	1ddaac0ce2d01033	1427058883	/data/HMASM/PGAs/supra	c57f47ac435d856dd6fc7a2	16526068
SRS015947	supragingival_plaque	/data/Illumina/supraging	ff7af2dbc667a7ec63	3333821406	/data/HMASM/PGAs/supra	3d6dbcf244d919e3eccc085	11692263
SRS017139	supragingival_plaque	/data/Illumina/supraging	afb7f0249c41134d2	7052164791	/data/HMASM/PGAs/supra	95026fe777887c1c7255cf8	38642320
SRS047100	supragingival_plaque	/data/Illumina/supraging	b5b362b3ddb46ba0	3350357258	/data/HMASM/PGAs/supra	63d015d48c5c9d8b5cd47d	17281817
SRS052876	supragingival_plaque	/data/Illumina/supraging	ff9e99a5113e8109c	4381858130	/data/HMASM/PGAs/supra	0bf79e90741fe71437a0016	30824961
SRS015158	supragingival_plaque	/data/Illumina/supraging	ff65fd6e9c1b0b585	4719616562	/data/HMASM/PGAs/supra	7550e9b33f4df5492dbd19	22197273
SRS021477	supragingival_plaque	/data/Illumina/supraging	453abf5aedbdabaff	4331138919	/data/HMASM/PGAs/supra	077f0e1ab650599a5cbef34	34150193
SRS064423	tongue_dorsum	/data/Illumina/tongue_d	20d964486e238840	5505444586	/data/HMASM/PGAs/tongu	e4063ff433664e0f3ec9756	33040419
SRS013506	buccal_mucosa	/data/Illumina/buccal_m	1c0bdf458fe597b36	1465158100	/data/HMASM/PGAs/bucca	f8cbc23b5b653506a0dcc28	6868205
SRS024347	buccal_mucosa	/data/Illumina/buccal_m	971c25ce7459bc811	820122950	/data/HMASM/PGAs/bucca	6d28fbea7ffef63fbae190b4	5638228
SRS017044	anterior_nares	/data/Illumina/anterior_	28eee9f17d9d34a91	91197174	/data/HMASM/PGAs/anter	b819ad552f714885c9aad1	1520625
SRS014472	buccal_mucosa	/data/Illumina/buccal_m	82559820fcaec607d	181856675	/data/HMASM/PGAs/bucca	a3911fc3fd2e4e55c0c90dc	1290240
SRS015040	buccal_mucosa	/data/Illumina/buccal_m	791ce399ab1a51ff2	702387979	/data/HMASM/PGAs/bucca	c1e79301160541efb52701	4509331
SRS015799	buccal_mucosa	/data/Illumina/buccal_m	54201a11fb25cd05a	969863949	/data/HMASM/PGAs/bucca	a19fe35aeb6ef61f0165146	5352202
SRS016088	buccal_mucosa	/data/Illumina/buccal_m	adbdc5b00803c204	97144961	/data/HMASM/PGAs/bucca	65e6d101b7b0b4a0964391	1128653
SRS014474	palatine_tonsils	/data/Illumina/palatine_	2e12e7fc5250d4626	257104096	/data/HMASM/PGAs/palat	c1712567efd586691d9004	2396587
SRS022713	stool	/data/Illumina/stool/SRS	a9c10a312b4931c46	6306825384	/data/HMASM/PGAs/stool_	ea7419891d8a4e4c893b00	17462835
SRS015269	anterior_nares	/data/Illumina/anterior_	7a56000f9a9306745	40433896	/data/HMASM/PGAs/anter	1c23fea2c0c6c009a0f25cea	533013
SRS024015	tongue_dorsum	/data/Illumina/tongue_d	7754c9d92adb8814	6506136980	/data/HMASM/PGAs/tongu	9f2af318723b14f6089820f	26140523
SRS016292	anterior_nares	/data/Illumina/anterior_	5d9905f6c8516e086	85435039	/data/HMASM/PGAs/anter	c5d13233d07239875fe2a3	1730811
SRS016513	anterior_nares	/data/Illumina/anterior_	5a9e6a77146ff6e06	25487311	/data/HMASM/PGAs/anter	f1e61934810f44d5df3bf39	58896
SRS019986	anterior_nares	/data/Illumina/anterior_	701ecd9a9a85ca02f	220157879	/data/HMASM/PGAs/anter	a7a9ceb2fb79500432e076	3327812
SRS056906	anterior_nares	/data/Illumina/anterior_	cfbac7182d439ae2b	374071756	/data/HMASM/PGAs/anter	d21eeb25240fdc38e2986b	2430089
SRS012281	buccal_mucosa	/data/Illumina/buccal_m	e441c4a0128835001	87026816	/data/HMASM/PGAs/bucca	62fa1ba7a98c90d2258740	589995
SRS016533	buccal_mucosa	/data/Illumina/buccal_m	82f0837e9f60dc2e7	487607069	/data/HMASM/PGAs/bucca	76a09abf52fba300bc25cc7	3700040
SRS019587	buccal_mucosa	/data/Illumina/buccal_m	f997596209df58c11	1662452218	/data/HMASM/PGAs/bucca	81ba9aae894ede4df87185	6029553
SRS024140	buccal_mucosa	/data/Illumina/buccal_m	19ca81fab81d34f5f5	815819851	/data/HMASM/PGAs/bucca	a1f7f4e0917391c45226da7	5567045

SRS043676	buccal_mucosa	/data/Illumina/buccal_m	48962e5fd0f95722e	1724582664	/data/HMASM/PGAs/bucca	1c2f1b48eb3ff4d4cbee37a	6360570
SRS019064	right_retroauricular_crease	/data/Illumina/right_retr	5d9a61a229b6e95d	1266269195	/data/HMASM/PGAs/right_	411d733ef04dfbb2f6473bc	17394160
SRS014313	stool	/data/Illumina/stool/SRS	436f8928f69414f50a	7395358011	/data/HMASM/PGAs/stool_	cc13fa1d3e869777f0598a9	15604043
SRS016018	stool	/data/Illumina/stool/SRS	d6b2f355b7acc7a13	5391230940	/data/HMASM/PGAs/stool_	6ccb5f4375400f8f47a1d02	36345825
SRS016095	stool	/data/Illumina/stool/SRS	bce76265052f0d1fb	7736266200	/data/HMASM/PGAs/stool_	fc5fe74c91b15270673d0a3	49687566
SRS016092	supragingival_plaque	/data/Illumina/supraging	fe88d92980890e652	3588793218	/data/HMASM/PGAs/supra	abf2ea99a57da45d4206d0	14620007
SRS016753	stool	/data/Illumina/stool/SRS	e19674905ca2c0558	7927683465	/data/HMASM/PGAs/stool_	8137a059693902c45670d6	19628395
SRS017814	supragingival_plaque	/data/Illumina/supraging	02a597993dd9f5aa7	3201155257	/data/HMASM/PGAs/supra	d6dea2015378c795e293b9	11930260
SRS047014	stool	/data/Illumina/stool/SRS	aa6b1bb01dbd1ae3	6514890014	/data/HMASM/PGAs/stool_	4cff1361f5e8d6d65872329	46338740
SRS013533	supragingival_plaque	/data/Illumina/supraging	1f10a6d475fbf7b09	3027568431	/data/HMASM/PGAs/supra	718e53e6e540b17e7fcc0bf	45334049
SRS014578	supragingival_plaque	/data/Illumina/supraging	675e435133e476ee	3368442105	/data/HMASM/PGAs/supra	8f073a68ab7b89fb104335	32451927
SRS016225	tongue_dorsum	/data/Illumina/tongue_d	6bb6c6cb605a2c45a	7254201150	/data/HMASM/PGAs/tongu	a66ea7bd6ba1cc30b9246c	25825630
SRS047634	supragingival_plaque	/data/Illumina/supraging	089e252f2bb4d89a1	4804163593	/data/HMASM/PGAs/supra	d056f5ea682522a7620ce7	57445488
SRS044373	tongue_dorsum	/data/Illumina/tongue_d	e7776fc6cb0fd9690	6649589799	/data/HMASM/PGAs/tongu	3607248423cb29b5f38d80	31479200
SRS019027	throat	/data/Illumina/throat/SR	eeee1b1361e7ff547	1890759330	/data/HMASM/PGAs/throa	245ec3fe477c73feba27803	18948399
SRS022645	anterior_nares	/data/Illumina/anterior_	ce202d8eba5a31a53	25266970	/data/HMASM/PGAs/anter	1f5076a37991db792664c2	244880
SRS047219	tongue_dorsum	/data/Illumina/tongue_d	97ea612549342cfed	5892787878	/data/HMASM/PGAs/tongu	d2e9d0007481717a9c5413	33463544
SRS063288	tongue_dorsum	/data/Illumina/tongue_d	1a6e6fda3f9789759	5035366104	/data/HMASM/PGAs/tongu	36acb4047a1ba852d51771	23896433
SRS077736	tongue_dorsum	/data/Illumina/tongue_d	8791c34c3f09ae46f	5659358981	/data/HMASM/PGAs/tongu	ab69f8f81779928b3f17ee8	17724261
SRS011355	posterior_fornix	/data/Illumina/posterior_	c4dc2da9a02334e85	687350435	/data/HMASM/PGAs/poste	bb066084f4b6204c372644	660934
SRS015073	posterior_fornix	/data/Illumina/posterior_	0ae00a03617119572	41534366	/data/HMASM/PGAs/poste	98818f0aac9db534bf5317c	804367
SRS052756	posterior_fornix	/data/Illumina/posterior_	99d708b3aaa8e998	28093376	/data/HMASM/PGAs/poste	f86d9dab1583a47001aca47	383435
SRS011302	stool	/data/Illumina/stool/SRS	f5db49814f2bafb61	9205327005	/data/HMASM/PGAs/stool_	a38be79520d0cbbc42920b	38120874
SRS014901	anterior_nares	/data/Illumina/anterior_	7d0b7d4e22778141	169551612	/data/HMASM/PGAs/anter	fbfa02dbbbf6f6a35bf31de8	2833137
SRS018671	anterior_nares	/data/Illumina/anterior_	7548b06b37038440	25173317	/data/HMASM/PGAs/anter	00950c3ece80c3e328acc1b	138689
SRS019067	anterior_nares	/data/Illumina/anterior_	cddfd913de6863862	43457527	/data/HMASM/PGAs/anter	88e6e872dc7cee2ced467f0	450374
SRS017521	stool	/data/Illumina/stool/SRS	83a6e0b8e5599409	7507666649	/data/HMASM/PGAs/stool_	05dca62e6d546cf6383b5c3	55927925
SRS049712	stool	/data/Illumina/stool/SRS	089a09affa334ebf58	8564751638	/data/HMASM/PGAs/stool_	d8fa12fa2987da2293d198c	31415071
SRS020261	left_retroauricular_crease	/data/Illumina/left_retro	e79a5e2b0d3e82dc	2669224497	/data/HMASM/PGAs/left_	f3a8495b36f30ca597c9d5b	5191844
SRS011105	anterior_nares	/data/Illumina/anterior_	c64496438a5cd71c2	273917947	/data/HMASM/PGAs/anter	207b675388d67eae8e6d49	1080065
SRS019029	subgingival_plaque	/data/Illumina/subgingiv	e598c79ed8a7f60c2	2010009104	/data/HMASM/PGAs/subgi	c2715134da6ac01ef5b6385	27818106
SRS013836	supragingival_plaque	/data/Illumina/supraging	c29419c674e8477c2	2773824100	/data/HMASM/PGAs/supra	57e1e9ccd067e87a2a92c5	27652794
SRS019015	left_retroauricular_crease	/data/Illumina/left_retro	a3c1348de933d8b9	388850339	/data/HMASM/PGAs/left_	8d27fbc54e272bb8b2c1b1f	3326038
SRS024596	left_retroauricular_crease	/data/Illumina/left_retro	8bac9dd877bf14806	2893430734	/data/HMASM/PGAs/left_	195353239845c27de3401a	3552124
SRS018661	buccal_mucosa	/data/Illumina/buccal_m	d1235d450f1beaac1	343365315	/data/HMASM/PGAs/bucca	06d6bcf80b82645c9e833d1	2974218
SRS014629	posterior_fornix	/data/Illumina/posterior_	5059023190e6561e	78035221	/data/HMASM/PGAs/poste	8b3b3760741d5e8a64414f	460173
SRS017497	posterior_fornix	/data/Illumina/posterior_	39892adc0c54c26bb	680476238	/data/HMASM/PGAs/poste	57b5125f5e8f12961b06753	3702961
SRS045254	buccal_mucosa	/data/Illumina/buccal_m	617e4cab50b9c2adf	424550621	/data/HMASM/PGAs/bucca	fe027c3478a536e1db20e9f	2767320
SRS015062	throat	/data/Illumina/throat/SR	96c455225ea351fcc	117648278	/data/HMASM/PGAs/throa	ec220d321311e44386aae1	1957540
SRS017713	tongue_dorsum	/data/Illumina/tongue_d	7c86687f543440627	4323175091	/data/HMASM/PGAs/tongu	06d13961d7b7cd64780528	16974916
SRS012291	anterior_nares	/data/Illumina/anterior_	12775f5df6e71961f	22919793	/data/HMASM/PGAs/anter	42e2b790019885ccdc6e00	9123
SRS015272	tongue_dorsum	/data/Illumina/tongue_d	38f18a55c2f51cac4b	5023537757	/data/HMASM/PGAs/tongu	750ce7976c60ddcc641414	20232989
SRS013947	palatine_tonsils	/data/Illumina/palatine_	fa69ae22d30716a84	2008200347	/data/HMASM/PGAs/palat	eda14e34ddb64bcc856090	13280119
SRS016434	anterior_nares	/data/Illumina/anterior_	86e0aaf3e6c83efda	43109906	/data/HMASM/PGAs/anter	98ca0b4eef8568c73f14b84	22071
SRS020868	anterior_nares	/data/Illumina/anterior_	97e0a29281ea9bba	64650187	/data/HMASM/PGAs/anter	5021c0fcc75699620ae0f28	70214
SRS023829	stool	/data/Illumina/stool/SRS	e05b5ae0a34e7488f	8288138311	/data/HMASM/PGAs/stool_	8c8fa70893192e2d126f8c0	8703778
SRS077730	stool	/data/Illumina/stool/SRS	d58b0c549ba68aae	7218428561	/data/HMASM/PGAs/stool_	c9cab97208e148d581d7b7	32387763
SRS042628	stool	/data/Illumina/stool/SRS	e241246ce3eada625	7031649766	/data/HMASM/PGAs/stool_	c307ec266a6e9a9d27e26a	37495761
SRS015063	supragingival_plaque	/data/Illumina/supraging	574e6ab1da1b5d9a	4208218446	/data/HMASM/PGAs/supra	ec88016b1b12425b8ae5c5	17665913
SRS016541	supragingival_plaque	/data/Illumina/supraging	a2808c4f9b25d0269	3539644561	/data/HMASM/PGAs/supra	b62bd52e70f6698c61d9f64	21591707

SRS015369	stool	/data/Illumina/stool/SRS	269fbd861dbcd2971	5138506345	/data/HMASM/PGAs/stool	02f069c8edab7a318c93e5b	15286266
SRS015436	buccal_mucosa	/data/Illumina/buccal_m	7157262c7981200ac	1548894002	/data/HMASM/PGAs/bucca	6c1c1d2a3225e19d49ec79	8246374
SRS017013	buccal_mucosa	/data/Illumina/buccal_m	03e2433bd09f9decb	1678651407	/data/HMASM/PGAs/bucca	4af1e8bc462913671d97b4	7514904
SRS024649	supragingival_plaque	/data/Illumina/supraging	cfb006f35dae51e7c	3993467031	/data/HMASM/PGAs/supra	2e4326154bc16a30f5778ab	30705977
SRS053630	supragingival_plaque	/data/Illumina/supraging	5613b235d4bf2d461	3630048576	/data/HMASM/PGAs/supra	65f1bb2609bef1a9ad84d9f	11498912
SRS015989	supragingival_plaque	/data/Illumina/supraging	085712e37dfa7be29	4404566578	/data/HMASM/PGAs/supra	5c340d2c819a2f6e7f186ac	19570229
SRS017076	tongue_dorsum	/data/Illumina/tongue_d	81e84ea9bc18c6c6e	4091201191	/data/HMASM/PGAs/tongu	ad880e42c49571e4c1eb00	16441991
SRS015395	tongue_dorsum	/data/Illumina/tongue_d	9cd5650085440f69e	4883257745	/data/HMASM/PGAs/tongu	22f79eb0ce23a97ed87bb9	15682050
SRS019022	tongue_dorsum	/data/Illumina/tongue_d	299192f17c8132696	7005156728	/data/HMASM/PGAs/tongu	3aefc8d82acfe2a52f966fa0	21283888
SRS016529	tongue_dorsum	/data/Illumina/tongue_d	ef57499a5e6d8e457	5766041890	/data/HMASM/PGAs/tongu	fc8a8280de74a8ffcfa34047	10647209
SRS014687	attached_keratinized_gingiv	/data/Illumina/attached_	e7db62f393c32197d	6856738380	/data/HMASM/PGAs/attac	2e783ebc3cdd3cd17e1b0d	3019305
SRS015060	attached_keratinized_gingiv	/data/Illumina/attached_	7633c105e0bf63fad	2456628633	/data/HMASM/PGAs/attac	d8c743e6fc1807fa18d940c	12445413
SRS014126	buccal_mucosa	/data/Illumina/buccal_m	78e36dd3b149af2bc	887285562	/data/HMASM/PGAs/bucca	0486616d57644fb5d9041e	2015448
SRS053437	anterior_nares	/data/Illumina/anterior_	6b8ace391b7797a97	127011157	/data/HMASM/PGAs/anter	9eea9d7cb37edad750ec2e	1131410
SRS014473	attached_keratinized_gingiv	/data/Illumina/attached_	47dcb8d6485d9a7b	3873220342	/data/HMASM/PGAs/attac	d6a2bbe13d2b7c04d441cd	11361631
SRS019976	buccal_mucosa	/data/Illumina/buccal_m	21a3d9c950dc233ff	555116795	/data/HMASM/PGAs/bucca	ee9de42beb28ade517e4a0	3247770
SRS056796	posterior_fornix	/data/Illumina/posterior_	0d36b23a7f641a90e	2442677934	/data/HMASM/PGAs/poste	6bcce8def1b24e64713741	1027693
SRS022079	buccal_mucosa	/data/Illumina/buccal_m	4e21f1a85745a317c	281148547	/data/HMASM/PGAs/bucca	f099fb13485ddf5cc77170c	2873832
SRS064376	posterior_fornix	/data/Illumina/posterior_	da3023775be6f6c27	237705847	/data/HMASM/PGAs/poste	49e6ca2ed3b302ea41ca0a	785131
SRS053335	stool	/data/Illumina/stool/SRS	a56ffc86f97768c45c	7821707918	/data/HMASM/PGAs/stool	eebf24414b0484427d4b9e	31383947
SRS015133	stool	/data/Illumina/stool/SRS	45f0f52fd5c7940d6a	7093323162	/data/HMASM/PGAs/stool	0ce08fc709d14b51216ece0	44262135
SRS063040	stool	/data/Illumina/stool/SRS	b4254fe39a37a5ddb	8131960445	/data/HMASM/PGAs/stool	91afb7442acc5e76cb77a58	49545695
SRS017849	left_retroauricular_crease	/data/Illumina/left_retro	50452b9b04db83d1	5041006676	/data/HMASM/PGAs/left_r	f484289681d065f861b28ec	7363015
SRS019063	left_retroauricular_crease	/data/Illumina/left_retro	de7fb60a1680c08ab	443518114	/data/HMASM/PGAs/left_r	84d314d21441ef05fc642ac	7312606
SRS044742	posterior_fornix	/data/Illumina/posterior_	3c92bdb9d8e93ce6f	23342757	/data/HMASM/PGAs/poste	0b07f90763266a0275704e	648335
SRS012273	stool	/data/Illumina/stool/SRS	e177e4d1f50dee558	7784856202	/data/HMASM/PGAs/stool	23a457c77231cae9996e34	62067921
SRS018133	stool	/data/Illumina/stool/SRS	35332f63c242a7b00	8372215007	/data/HMASM/PGAs/stool	a91f105487b8680a1b398c	19565398
SRS048164	stool	/data/Illumina/stool/SRS	f510a1cf78cde3e59	6111559776	/data/HMASM/PGAs/stool	1aef7e0fb6a15f0d219f854a	41121361
SRS015899	supragingival_plaque	/data/Illumina/supraging	9f313ceea9cd82ab7	4951561119	/data/HMASM/PGAs/supra	8f9e08986d0f8f7f52b5c07	22330019
SRS024138	tongue_dorsum	/data/Illumina/tongue_d	28cc7e309146d040a	6498343577	/data/HMASM/PGAs/tongu	ffca3d0fa6c29311fe250f79	27902102
SRS024144	supragingival_plaque	/data/Illumina/supraging	d572d5f93bf1eb16e	1975723870	/data/HMASM/PGAs/supra	9db1ea1edc969fb184df3d1	21239322
SRS019127	throat	/data/Illumina/throat/SR	d7fa58e98d39918aa	2898218223	/data/HMASM/PGAs/throa	87904c1e75067dc4fb3bd4	24783494
SRS020220	tongue_dorsum	/data/Illumina/tongue_d	39e010a4f1619e052	5886259826	/data/HMASM/PGAs/tongu	5458b0724a4a87c253b0b2	27440108
SRS047225	anterior_nares	/data/Illumina/anterior_	fe256fc2b85f1a1ee2	228536579	/data/HMASM/PGAs/anter	e616712b9f4ecb49e1d95a	3212943
SRS017821	stool	/data/Illumina/stool/SRS	5bd1b98557bcbd8d	7183549186	/data/HMASM/PGAs/stool	161432cedf03bd5da01214	42623668
SRS019030	stool	/data/Illumina/stool/SRS	4ddf43da50f22fe84	5885270500	/data/HMASM/PGAs/stool	70b49a9b947d32a5ad9324	32844167
SRS019685	stool	/data/Illumina/stool/SRS	97311788a3c26d11	7255856148	/data/HMASM/PGAs/stool	34566c1d9f7382293313e4	42743395
SRS054590	stool	/data/Illumina/stool/SRS	0b6f2cd0e53f00af8c	6018472168	/data/HMASM/PGAs/stool	72cffafbcfd86dccefd95399	12671442
SRS015650	supragingival_plaque	/data/Illumina/supraging	6a893f992e0cd00a6	2053418761	/data/HMASM/PGAs/supra	0eff352c458e54e698d7a46	12138687
SRS017088	supragingival_plaque	/data/Illumina/supraging	4e0373f06f7d93274	1138915998	/data/HMASM/PGAs/supra	3f2ac538501bafb44f96169	3851320
SRS017691	supragingival_plaque	/data/Illumina/supraging	5000b077eb45bd72	4946251361	/data/HMASM/PGAs/supra	274d74e4495129a076549d	48875974
SRS015752	anterior_nares	/data/Illumina/anterior_	7ac2968e0963e8c4b	77260801	/data/HMASM/PGAs/anter	5f095915fdadcf87c8289c0	1740870
SRS063351	palatine_tonsils	/data/Illumina/palatine_	0f51c36ffec8a38aaf	315629571	/data/HMASM/PGAs/palat	7f9ac09517013213bfd8ee8	3884939
SRS023850	posterior_fornix	/data/Illumina/posterior_	0ff21ddbd486fb883	51505268	/data/HMASM/PGAs/poste	acce99652228b690130a2a	474916
SRS013637	anterior_nares	/data/Illumina/anterior_	5acc0e71e5031a4a0	39696236	/data/HMASM/PGAs/anter	dfda270a537761b1c6dea8	29733
SRS018981	anterior_nares	/data/Illumina/anterior_	3f0d8f31f4d91df4c9	75526388	/data/HMASM/PGAs/anter	ab87eb7c0f59091521743	929837
SRS023557	tongue_dorsum	/data/Illumina/tongue_d	a1b50eb89d7af4c83	3548487434	/data/HMASM/PGAs/tongu	616d7d2afbd28d72fbf6e5f	15884282
SRS047844	anterior_nares	/data/Illumina/anterior_	4de90f78170bebdca	212825997	/data/HMASM/PGAs/anter	f1aa7418263837024306f71	5080
SRS024318	tongue_dorsum	/data/Illumina/tongue_d	14b2e472b23b54ea	4688006321	/data/HMASM/PGAs/tongu	ba4f4d30df3448555a9d35a	33905606
SRS024580	tongue_dorsum	/data/Illumina/tongue_d	0573301108f1732a6	6031328132	/data/HMASM/PGAs/tongu	607418cda681b78bb5c70a	22972549

SRS023987	buccal_mucosa	/data/Illumina/buccal_m	84a3cb631bec57923	1215927949	/data/HMASM/PGAs/bucca	d820a29f5602ea31766da51	5189168
SRS051882	stool	/data/Illumina/stool/SRS	c6d393dceaf2ef4af8	6833811422	/data/HMASM/PGAs/stool_	4536d142c0b77ca7604caeb	32492422
SRS078176	stool	/data/Illumina/stool/SRS	d7644cc2f61605102	5705152815	/data/HMASM/PGAs/stool_	230b60028bd3345094b442	37311954
SRS013170	supragingival_plaque	/data/Illumina/supraging	f820635eea95f13efb	4477479493	/data/HMASM/PGAs/supra	2ef7391f0ae2fe5953aef588	48958544
SRS016360	supragingival_plaque	/data/Illumina/supraging	94b9c0a4f86774560	4936532169	/data/HMASM/PGAs/supra	d7a55ea96b764e29d4a2d4	24571788
SRS017304	supragingival_plaque	/data/Illumina/supraging	63303046cb06d9a3c	3630980533	/data/HMASM/PGAs/supra	d6b77c26dea0b90aee1771	22360570
SRS075410	supragingival_plaque	/data/Illumina/supraging	ded5ce8d5b198ccdf	7673752491	/data/HMASM/PGAs/supra	ab2713077148345ac59a97a	22728807
SRS013948	throat	/data/Illumina/throat/SR	cf553ff0abedd8f24e	2091772740	/data/HMASM/PGAs/throa	d1755c1d8886804b60d051	17067806
SRS065335	throat	/data/Illumina/throat/SR	886d67b985db91c75	479710867	/data/HMASM/PGAs/throa	e5bf3bbe130ae7ea19de932	10260426
SRS053917	supragingival_plaque	/data/Illumina/supraging	19912fb5ccd92214c	8127165171	/data/HMASM/PGAs/supra	8d81d1fd56381d547f7d850	40792680
SRS014684	tongue_dorsum	/data/Illumina/tongue_d	b92c066acb50ad1a9	8144123962	/data/HMASM/PGAs/tongu	6f054b9fb7082c8d1c7cfee7	24217888
SRS065099	supragingival_plaque	/data/Illumina/supraging	d9271896a379a15e1	3159748117	/data/HMASM/PGAs/supra	51fd58b30b7938d75d9372	35412238
SRS014470	tongue_dorsum	/data/Illumina/tongue_d	16f221bd3f7dcbf40b	5614806525	/data/HMASM/PGAs/tongu	f27b0498aad91413cd0a780	33235121
SRS019607	tongue_dorsum	/data/Illumina/tongue_d	cf50cda052e608ab9	6322382226	/data/HMASM/PGAs/tongu	54bd27a98cc73bf441a844f	32365322
SRS017120	tongue_dorsum	/data/Illumina/tongue_d	50e4fd0613d256fe8	6459096536	/data/HMASM/PGAs/tongu	5205d5a4e913ed46f235a64	31467099
SRS053854	tongue_dorsum	/data/Illumina/tongue_d	8b370f130e3bced44	6228737237	/data/HMASM/PGAs/tongu	f870a0f6991de7fe33a5a32f	25457390
SRS018439	tongue_dorsum	/data/Illumina/tongue_d	9dc0c7c7382ff8797b	7623716756	/data/HMASM/PGAs/tongu	03ec2fcbb5338b2f0d94025	31428233
SRS054962	posterior_fornix	/data/Illumina/posterior_	432e5a7fa36c0d4bd	162822972	/data/HMASM/PGAs/poste	e31ca981a9f98570741c910	395512
SRS024388	stool	/data/Illumina/stool/SRS	9e82bf9d0a7c42e42	9763641284	/data/HMASM/PGAs/stool_	22cca7b453f5bbb84581f09	22928567
SRS016746	supragingival_plaque	/data/Illumina/supraging	c9cec6e2428fce7599	4670708822	/data/HMASM/PGAs/supra	7a8c116f1dfedc783577d1b	33250317
SRS017445	supragingival_plaque	/data/Illumina/supraging	98403881e2b509e66	7447938884	/data/HMASM/PGAs/supra	9200408cb5361032643f494	32025539
SRS018157	supragingival_plaque	/data/Illumina/supraging	aa6eb74ea5eeef9bf5	7093734418	/data/HMASM/PGAs/supra	6cc0f1dfed96a61d7833fe79	43745137
SRS023926	tongue_dorsum	/data/Illumina/tongue_d	65a8a59d46be155b0	6920772914	/data/HMASM/PGAs/tongu	14b625d7cdecec0b8613828	35509777
SRS024277	tongue_dorsum	/data/Illumina/tongue_d	b3d1eea58c826c075	7827546900	/data/HMASM/PGAs/tongu	c60746361ee8dd20f96dd8c	23978712
SRS051791	tongue_dorsum	/data/Illumina/tongue_d	ee2ad30c8307c32f3	6265846298	/data/HMASM/PGAs/tongu	4361df7d694700082d1b378	29957509
SRS047254	anterior_nares	/data/Illumina/anterior_r	02a86d8b1ac49d684	116848099	/data/HMASM/PGAs/anter	5a00ec6a699070b5fa90501	473899
SRS047708	anterior_nares	/data/Illumina/anterior_r	09ecce97d4864ad44	1801044	/data/HMASM/PGAs/anter	b69998f66ce8822b2d2f677	1370
SRS017156	anterior_nares	/data/Illumina/anterior_r	26da99039a85dd19c	36748149	/data/HMASM/PGAs/anter	248666cb50fea55d511789b	440447
SRS046686	buccal_mucosa	/data/Illumina/buccal_m	d05e0b4a4e580676e	2989019610	/data/HMASM/PGAs/bucca	ba2974ce6cc830387cd118e	5031601
SRS017820	anterior_nares	/data/Illumina/anterior_r	fed639f4984b65d36	96023193	/data/HMASM/PGAs/anter	660dc3685e1584165a8e2d	1465578
SRS016196	buccal_mucosa	/data/Illumina/buccal_m	3e9466c06b68459dc	433756489	/data/HMASM/PGAs/bucca	08bc3703cbb44a5ebe065c6	3426466
SRS015450	anterior_nares	/data/Illumina/anterior_r	eefc0dcf2d52ca5251	19861830	/data/HMASM/PGAs/anter	8e25c4a7c4ebc4b73a11ade	109684
SRS013158	stool	/data/Illumina/stool/SRS	fbccf546dfb3547c2d	8439526268	/data/HMASM/PGAs/stool_	d4454162796964a1cab288	17898779
SRS062878	hard_palate	/data/Illumina/hard_pala	b0733a4b194a8189c	3816121779	/data/HMASM/PGAs/hard_	67578211aa3424d978d09a	19212886
SRS016954	stool	/data/Illumina/stool/SRS	d4ecedcefb1300a25	8347179620	/data/HMASM/PGAs/stool_	6ce676a6a3909436c8cb541	34259512
SRS014466	mid_vagina	/data/Illumina/mid_vagir	5e4a7b783cd037422	49164669	/data/HMASM/PGAs/mid_	69e153fee3f96c16576427e	824672
SRS052330	posterior_fornix	/data/Illumina/posterior_	2c7cedd3a9f8ba298	53541583	/data/HMASM/PGAs/poste	5f72f109aa7856fd24dccc7a	499176
SRS023534	buccal_mucosa	/data/Illumina/buccal_m	e796cf9f1693e3a735	56767428	/data/HMASM/PGAs/bucca	06700308ac9ae9555975c70	748935
SRS065133	buccal_mucosa	/data/Illumina/buccal_m	36fd87eabd1db18df	31424108	/data/HMASM/PGAs/bucca	5bdb06261a6b9d70116baf	52540
SRS015225	posterior_fornix	/data/Illumina/posterior_	1bb0d1d9633c6d6a9	92034287	/data/HMASM/PGAs/poste	bac319cdd74448a4d37d59	850154
SRS056210	anterior_nares	/data/Illumina/anterior_r	9b2f74b8067e6f205	19021776	/data/HMASM/PGAs/anter	990df8f3adc42e288bbbcde	18757
SRS054776	buccal_mucosa	/data/Illumina/buccal_m	1a8ea04bf712c9268	776881191	/data/HMASM/PGAs/bucca	74ed91d5761a890f0e9b22b	7292833
SRS015425	posterior_fornix	/data/Illumina/posterior_	0fbedd414cafa5199	386258813	/data/HMASM/PGAs/poste	074a688b3586d87b442d37	757649
SRS021969	posterior_fornix	/data/Illumina/posterior_	7ecd1754649f0f9f17	54558798	/data/HMASM/PGAs/poste	a10475301347ea049fda753	651494
SRS013800	stool	/data/Illumina/stool/SRS	dfe83bc44e52ab176	7797737041	/data/HMASM/PGAs/stool_	8e8fab5da8a92a793c88a62	36322041
SRS014683	stool	/data/Illumina/stool/SRS	ea0f4a7dba761d0cf6	5633455893	/data/HMASM/PGAs/stool_	d35fbbfe9c43eb4fa67747da	29150816
SRS043411	stool	/data/Illumina/stool/SRS	5342570a831072df3	4377090854	/data/HMASM/PGAs/stool_	e2796450ed7fb23ffdb3ab0	31740118
SRS056519	stool	/data/Illumina/stool/SRS	f7e1d8b1bf57b5b52	8521882572	/data/HMASM/PGAs/stool_	afc2f6038340a14386380b2	28111055
SRS024561	supragingival_plaque	/data/Illumina/supraging	349d5577a44dd0eac	3129078140	/data/HMASM/PGAs/supra	e9c713345c166752835457a	34578963
SRS044366	supragingival_plaque	/data/Illumina/supraging	bdaca1d8cd467fc91b	788964455	/data/HMASM/PGAs/supra	8a4b8ac10c9d5e31134cc58	5607408

SRS058053	supragingival_plaque	/data/Illumina/supraging	b4c3a618bfa7ded56	4344463099	/data/HMASM/PGAs/supra	4900c76db66237dc08717c	35670102
SRS064493	supragingival_plaque	/data/Illumina/supraging	59be8b335520442988	790451334	/data/HMASM/PGAs/supra	61443e11ba057776d57179	6222458
SRS018443	buccal_mucosa	/data/Illumina/buccal_m	c29a640185686aa16	6243680202	/data/HMASM/PGAs/bucca	a4fc1401ee8a2bcb217164c	37013184
SRS022145	buccal_mucosa	/data/Illumina/buccal_m	5f21cc15c5ad01fc21	1004091323	/data/HMASM/PGAs/bucca	e9b98c21a3904ae781a698	2401085
SRS050029	buccal_mucosa	/data/Illumina/buccal_m	b4d62653a1e426c2c	1597013079	/data/HMASM/PGAs/bucca	87cf060b59c05112a2f0206	7779965
SRS018769	posterior_fornix	/data/Illumina/posterior_	d2736e01302f9a9b8	66894690	/data/HMASM/PGAs/poste	d5a4a60fb0a697e3a8aba06	666061
SRS019989	posterior_fornix	/data/Illumina/posterior_	1d7c116cd6046023	277723533	/data/HMASM/PGAs/poste	0f8a9e7512a67e4f2c5323fe	654909
SRS019081	right_retroauricular_crease	/data/Illumina/right_retr	96c25fa0b7f616f3f0	577652440	/data/HMASM/PGAs/right_	4713a5e9c4b315d4f33e338	3548704
SRS016585	stool	/data/Illumina/stool/SRS	ae5b7e39122f50dd2	8506928512	/data/HMASM/PGAs/stool_	37959ff77d096e5a486ebae	4951192
SRS049164	stool	/data/Illumina/stool/SRS	3fffee8c4dcb718535	4413272733	/data/HMASM/PGAs/stool_	8b3f365153e949659a8e70	42268059
SRS013164	tongue_dorsum	/data/Illumina/tongue_d	70a6cd73685c2b3da	6540431245	/data/HMASM/PGAs/tongu	06fe7e4028b46c0f8bc6047	25304695
SRS019225	supragingival_plaque	/data/Illumina/supraging	a252018e7ef5e4c67	2053161743	/data/HMASM/PGAs/supra	9562c5e8d9f6f08cf23b2cec	16189743
SRS023958	tongue_dorsum	/data/Illumina/tongue_d	a355661f681915df9	7203685247	/data/HMASM/PGAs/tongu	8c4bb6138392259a4b25ba	23084577
SRS053584	supragingival_plaque	/data/Illumina/supraging	94a4ef713a78c77d6	3076816898	/data/HMASM/PGAs/supra	23a85b17a8a3baac4d62e2	17959173
SRS064219	supragingival_plaque	/data/Illumina/supraging	d3987098cf470f23b	283786921	/data/HMASM/PGAs/supra	af90d289a7d37d3f5019c5f	631101
SRS015209	tongue_dorsum	/data/Illumina/tongue_d	2cbda471e667206db	7383503621	/data/HMASM/PGAs/tongu	b35f82782cc3e5c70b25502	29246283
SRS013881	buccal_mucosa	/data/Illumina/buccal_m	0be0baa5f4074ea18	666575143	/data/HMASM/PGAs/bucca	8275d6523da97f1d553d5a	5297970
SRS013687	stool	/data/Illumina/stool/SRS	8d1a5f01482258800	8263639495	/data/HMASM/PGAs/stool_	b6c00714f40ead298c7e1ee	46828405
SRS042428	posterior_fornix	/data/Illumina/posterior_	184732444f4b61f6d	121168606	/data/HMASM/PGAs/poste	e6166a5b9f1d81566a8bd0	900747
SRS021948	stool	/data/Illumina/stool/SRS	e4d15cb6bba07e5d0	6968379247	/data/HMASM/PGAs/stool_	7fdf2a5bf6b14889bb2c07fe	46981620
SRS024075	stool	/data/Illumina/stool/SRS	939929b8058d00ad	9874686091	/data/HMASM/PGAs/stool_	97e4ce713909a1acdda2d9	31220040
SRS021496	tongue_dorsum	/data/Illumina/tongue_d	e223036a4cab6164a	6958737506	/data/HMASM/PGAs/tongu	0dcb5c6799e20c41c498fc7	39889668
SRS021954	tongue_dorsum	/data/Illumina/tongue_d	30c161df6211b4f63	6436611560	/data/HMASM/PGAs/tongu	94134fb8b7547f59d00a802	23388638
SRS019980	supragingival_plaque	/data/Illumina/supraging	7459e4f9673b37245	5037523948	/data/HMASM/PGAs/supra	d0f541ba8c62b2e6545010	35706675
SRS013269	anterior_nares	/data/Illumina/anterior_	ceaee617a0af699a2	37318665	/data/HMASM/PGAs/anter	95464939cb4a167db649b4	28196
SRS014464	anterior_nares	/data/Illumina/anterior_	abbafcb579f489cfa4	45166211	/data/HMASM/PGAs/anter	5274d9d590d2cd6fa1a1b9	108264
SRS024637	tongue_dorsum	/data/Illumina/tongue_d	55866e00afadae5aa	3101127388	/data/HMASM/PGAs/tongu	d91c6a05d45250c1476969	19279183
SRS055426	tongue_dorsum	/data/Illumina/tongue_d	81b92bd1878ab53b	2519430196	/data/HMASM/PGAs/tongu	4810f9a00d5eae362de727	19644743
SRS064774	tongue_dorsum	/data/Illumina/tongue_d	a2d541e3e3a0706c	5880067289	/data/HMASM/PGAs/tongu	9e053a29588fad60bdab62	32516868
SRS056622	tongue_dorsum	/data/Illumina/tongue_d	446fe8153e811dc5a	6192761295	/data/HMASM/PGAs/tongu	37672c0a4e294f942e55971	27787762
SRS023914	stool	/data/Illumina/stool/SRS	1d1d8afaf49c84508	8775132332	/data/HMASM/PGAs/stool_	2b5fad8d6fe021e0c8b69b7	45922411
SRS077751	posterior_fornix	/data/Illumina/posterior_	bbec29dc15f17fe99	95502062	/data/HMASM/PGAs/poste	c38325db0d01d8510b9d85	613543
SRS011255	supragingival_plaque	/data/Illumina/supraging	af2d979d6aad3a9be	2800309914	/data/HMASM/PGAs/supra	045f55d84608ce137d5130	35326333
SRS012285	supragingival_plaque	/data/Illumina/supraging	6bd73cf266ec7ac6e	5243617214	/data/HMASM/PGAs/supra	92f68a8f669fb2af52955d4	37456262
SRS049283	buccal_mucosa	/data/Illumina/buccal_m	ac3c9a23c56c5186d	109443259	/data/HMASM/PGAs/bucca	c42d0680d118184d43c4dd	177094
SRS022536	supragingival_plaque	/data/Illumina/supraging	1c5a3a690de4a1e7c	6166352778	/data/HMASM/PGAs/supra	2dbe028f2539d13b30ef6d	46247637
SRS023964	supragingival_plaque	/data/Illumina/supraging	ffd38804cf5d443ccf	3070752061	/data/HMASM/PGAs/supra	a189bb6e7ab0c8dbfc8b13a	28713686
SRS047265	supragingival_plaque	/data/Illumina/supraging	70c93af4ba3abdff8c	985502690	/data/HMASM/PGAs/supra	fdcf3def0733a450929e62a	9955294
SRS011126	supragingival_plaque	/data/Illumina/supraging	bb43b9f75d6168a1	7482839519	/data/HMASM/PGAs/supra	1ed9e8a2141b6e446e8947	44199804
SRS016331	supragingival_plaque	/data/Illumina/supraging	01ca7dc7b12683375	4021632308	/data/HMASM/PGAs/supra	48b69955179134545d9d21	30336426
SRS064449	supragingival_plaque	/data/Illumina/supraging	f8b7f2e9d0c84ec4d	5258735958	/data/HMASM/PGAs/supra	ab2ce74687add7564a30cd	33103491
SRS023538	supragingival_plaque	/data/Illumina/supraging	02f1df2009ba31f7e	2691117437	/data/HMASM/PGAs/supra	da75de9732594867fc7032	17400405
SRS018591	tongue_dorsum	/data/Illumina/tongue_d	436bd144e8556d0c	5918785937	/data/HMASM/PGAs/tongu	e0e9fc9439d987ce9149e2c	27158317
SRS043663	tongue_dorsum	/data/Illumina/tongue_d	f52ee81718b8e0895	9031140685	/data/HMASM/PGAs/tongu	be46ceafb29a69fbf62b177	21742978
SRS064329	supragingival_plaque	/data/Illumina/supraging	7de6b2dbdf7dcbd1f	3946915869	/data/HMASM/PGAs/supra	258de90ecd6f6755ea52321	28169755
SRS044626	anterior_nares	/data/Illumina/anterior_	5b77077b6ab697cf5	28378901	/data/HMASM/PGAs/anter	5617c9a388d6e79cab9786	135125
SRS064809	buccal_mucosa	/data/Illumina/buccal_m	7904d3e2e39d9381	728838507	/data/HMASM/PGAs/bucca	26e812866eb2ce5e2a73d2	5718539
SRS014465	vaginal_introitus	/data/Illumina/vaginal_ir	116226db2b45252e	56384470	/data/HMASM/PGAs/vagin	f0532010e125ac61452ac37	779776
SRS051505	posterior_fornix	/data/Illumina/posterior_	10c6810ddadd1817f	85531389	/data/HMASM/PGAs/poste	814d1e09151aa8bbdbf14c	1628224
SRS056695	posterior_fornix	/data/Illumina/posterior_	adb04ee43bd519f19	194860332	/data/HMASM/PGAs/poste	09060715c1717795b4ee64	622691

SRS020386	anterior_nares	/data/Illumina/anterior_	82bb9ccde1ce0387	251423053	/data/HMASM/PGAs/anter	a0fa2d84d2f81d3b560246	2384263
SRS042284	stool	/data/Illumina/stool/SRS	278de2c819a7b05c	4567639394	/data/HMASM/PGAs/stool	67fab3e7c0db1b00f115f22	16598006
SRS020232	anterior_nares	/data/Illumina/anterior_	7cca5dd5cd1190a80	113838168	/data/HMASM/PGAs/anter	e55038228b68e5ccaaf294	1813679
SRS024620	left_retroauricular_crease	/data/Illumina/left_retro	a286cdc620002866	231203167	/data/HMASM/PGAs/left_	bd18a5f4077d0677e91790	1484500
SRS011111	posterior_fornix	/data/Illumina/posterior_	50bdc7a1bf4f03f82	5627497742	/data/HMASM/PGAs/post	6cb867f9851de93022f22ed	8492380
SRS053398	stool	/data/Illumina/stool/SRS	3d835547e83211e9	6998958787	/data/HMASM/PGAs/stool	cab457383ddec9e7d09d66	39810950
SRS075398	stool	/data/Illumina/stool/SRS	93fdc266e3e7f0693	7492829529	/data/HMASM/PGAs/stool	70d04438cc56f4e932954b	11948855
SRS014107	subgingival_plaque	/data/Illumina/subgingiv	4ded38a52bb60045	711858347	/data/HMASM/PGAs/subgi	19acea7d11b289f0e75273	10010015
SRS013261	right_retroauricular_crease	/data/Illumina/right_retr	c827a27dad7b7762	3803980092	/data/HMASM/PGAs/right_	278c9f45e2d94a93a00bfa3	4546485
SRS017851	right_retroauricular_crease	/data/Illumina/right_retr	b5146f7eb15c2a5c7	4018882567	/data/HMASM/PGAs/right_	b09696fd876b99837d34b6	6598076
SRS016267	stool	/data/Illumina/stool/SRS	f1363f7a77afb75c1	7064591714	/data/HMASM/PGAs/stool	8d1f179c0fb195d849fa57b	16999064
SRS024301	anterior_nares	/data/Illumina/anterior_	862af5894a70b2982	63294918	/data/HMASM/PGAs/anter	0bcbe7e1f3adc446cdde7e2	75297
SRS063287	buccal_mucosa	/data/Illumina/buccal_m	231f7c7d92adf31d0	1502784852	/data/HMASM/PGAs/bucc	24f1b61a745d149c26334a	5784881
SRS023346	stool	/data/Illumina/stool/SRS	8b10815ef21bbe294	5493110094	/data/HMASM/PGAs/stool	74e77210b4fd91ec8010ad	15507957
SRS015374	buccal_mucosa	/data/Illumina/buccal_m	0bce6a4a597c55ae8	394412321	/data/HMASM/PGAs/bucc	436c9e5876843d8441c37b	2710656
SRS014476	supragingival_plaque	/data/Illumina/supraging	6cc088082329f4f60	3276106972	/data/HMASM/PGAs/supr	66de58809f2eaf74846663	32635780
SRS051941	supragingival_plaque	/data/Illumina/supraging	9339557f8fa7f4e47	4714428570	/data/HMASM/PGAs/supr	ddf80848070f6048a8ace7c	31934568
SRS024381	supragingival_plaque	/data/Illumina/supraging	04e42c06b0ff7e96c	4063214614	/data/HMASM/PGAs/supr	b76ddf2378da2e112bbd85	26431851
SRS022734	posterior_fornix	/data/Illumina/posterior_	529e5640e1b2b395	47106850	/data/HMASM/PGAs/post	77d3ffdf7fd5a4aad608f589	566929
SRS047335	posterior_fornix	/data/Illumina/posterior_	16420b8713d694c2	508063252	/data/HMASM/PGAs/post	7d66fb95e62befb1103f42c	1382798
SRS016569	tongue_dorsum	/data/Illumina/tongue_d	4b1ef0c1b36eb6cdc	6938592217	/data/HMASM/PGAs/tong	59b075886669a80dc83c90	29425982
SRS017533	tongue_dorsum	/data/Illumina/tongue_d	21a715532317d6e3	4927743315	/data/HMASM/PGAs/tong	4fcb9b58c1498eb54ab6db	21140333
SRS058221	right_retroauricular_crease	/data/Illumina/right_retr	6cef1b2343eaf6d79	1504159899	/data/HMASM/PGAs/right_	efd602bc0e3881b67912ce	2704617
SRS017247	stool	/data/Illumina/stool/SRS	54a5eab82e72db2a	5996742787	/data/HMASM/PGAs/stool	cfd85e6eabfba8e8981f3cb	17018183
SRS013956	anterior_nares	/data/Illumina/anterior_	0bb9840948fc7515c	26099775	/data/HMASM/PGAs/anter	ee8db30db9aad7aaab76dd	294463
SRS013942	saliva	/data/Illumina/saliva/SRS	11cb282fd2bd32359	562432852	/data/HMASM/PGAs/saliv	c367e610eff5f5bbd143049	5543874
SRS046344	anterior_nares	/data/Illumina/anterior_	f8fee0fd797781a491	173219153	/data/HMASM/PGAs/anter	069cd7f48903b27dc37235	268111
SRS065179	anterior_nares	/data/Illumina/anterior_	27b2c9209bc56cbe2	13923530	/data/HMASM/PGAs/anter	c9a0dc9baadb8719d07455	55866
SRS023938	supragingival_plaque	/data/Illumina/supraging	13ea86f49534b7661	7458662603	/data/HMASM/PGAs/supr	56c5bc348d76a497e23e7c	33083741
SRS013949	supragingival_plaque	/data/Illumina/supraging	4ea6875fbfa899c5fb	5170278936	/data/HMASM/PGAs/supr	856dae93cfde3381872808	26653808
SRS024470	buccal_mucosa	/data/Illumina/buccal_m	51ba1e467d3039c9	658229477	/data/HMASM/PGAs/bucc	24aca5e3fc57e17317e6d8c	4202830
SRS020340	supragingival_plaque	/data/Illumina/supraging	2ebf01c3f090544af4	6347009986	/data/HMASM/PGAs/supr	c4d2b4f380065085a8da5a	22387496
SRS056157	buccal_mucosa	/data/Illumina/buccal_m	af25c4a86838c126a	308592799	/data/HMASM/PGAs/bucc	23b13f1e519856d14c732d	3927052
SRS056892	buccal_mucosa	/data/Illumina/buccal_m	54ed9c239337d008	1526631487	/data/HMASM/PGAs/bucc	791cafd967f3dcd6585e166	8523501
SRS024482	left_retroauricular_crease	/data/Illumina/left_retro	cd4ee71a7af859dba	1029044039	/data/HMASM/PGAs/left_	81f885fd6f3c04a6649cf480	6025453
SRS018145	tongue_dorsum	/data/Illumina/tongue_d	eb4d67933b9a35d8	7877517571	/data/HMASM/PGAs/tong	60993fa0f8b971b7cc1cf34	24164423
SRS057355	tongue_dorsum	/data/Illumina/tongue_d	e9917e02e22be2da	2657781014	/data/HMASM/PGAs/tong	6e8b5873cc085f121d6a21	17765208
SRS011397	anterior_nares	/data/Illumina/anterior_	e84aab4c2e80dccd0	164504217	/data/HMASM/PGAs/anter	8ac8219cdfe4a57b20f2068	1300603
SRS015051	anterior_nares	/data/Illumina/anterior_	b0f905a539a5b3336	36481771	/data/HMASM/PGAs/anter	6bdb6ba5d420e38b2c6db8	651945
SRS057692	tongue_dorsum	/data/Illumina/tongue_d	cdb2948371b190f59	7078038359	/data/HMASM/PGAs/tong	8eed671799a393a6306279	26164064
SRS062544	tongue_dorsum	/data/Illumina/tongue_d	b1de2c4e43a6a5d1	7461304161	/data/HMASM/PGAs/tong	ae862cd63277f442d51f0da	39172148
SRS011271	stool	/data/Illumina/stool/SRS	edd5a1f7f4e2d3529	9130425607	/data/HMASM/PGAs/stool	17ecb7d435431638115d7b	51152162
SRS022006	anterior_nares	/data/Illumina/anterior_	ceefbd37f300e4dc4	343943636	/data/HMASM/PGAs/anter	ba86e53b0cbbd2897af374	3455569
SRS019397	stool	/data/Illumina/stool/SRS	517815455f6fd1a1c	7632381867	/data/HMASM/PGAs/stool	0e83da32bbfba4084a719b	31298836
SRS043701	stool	/data/Illumina/stool/SRS	8f284e0601278a1e6	8386458700	/data/HMASM/PGAs/stool	d3e309eb7949bf1b73f00d	28333276
SRS045645	stool	/data/Illumina/stool/SRS	96be6fce6c216c76c	7608700061	/data/HMASM/PGAs/stool	61dd47a0481d69010891c6	36381077
SRS057478	stool	/data/Illumina/stool/SRS	9262efc5d054773d3	5273500571	/data/HMASM/PGAs/stool	826651d13d787f426da248	18144811
SRS064276	stool	/data/Illumina/stool/SRS	796b63b8614842c0	5841779220	/data/HMASM/PGAs/stool	633a991fdc81ce94ced9804	54557437
SRS022524	stool	/data/Illumina/stool/SRS	dcc7817684824506	1296094591	/data/HMASM/PGAs/stool	721a6f7ac1f953664e5c18c	28539400
SRS014691	subgingival_plaque	/data/Illumina/subgingiv	a619496d6099e0d1	1875764168	/data/HMASM/PGAs/subgi	9bcfbc8d6044f65bd4728ae	26326446

SRS018337	supragingival_plaque	/data/Illumina/supraging	27052d5e5dcc4b487	3101179889	/data/HMASM/PGAs/supra	fd02196be5711af45c9ee8b	13660289
SRS055298	posterior_fornix	/data/Illumina/posterior_	8ce924511b33bb08	142496941	/data/HMASM/PGAs/poste	a0a5df6e9479133361b91e	884407
SRS014235	stool	/data/Illumina/stool/SRS	097b85cd8e16cab9	6682789189	/data/HMASM/PGAs/stool_	e43f1162765f690b921347	64238279
SRS019974	tongue_dorsum	/data/Illumina/tongue_d	8ad68278ca06e9aff	6693219261	/data/HMASM/PGAs/tongu	f1f77a5323547a9ebe6f669	27744627
SRS018817	stool	/data/Illumina/stool/SRS	0ee123fb34329d1d9	7221657008	/data/HMASM/PGAs/stool_	29933f1fa9f8bbd983f6241	32891936
SRS063478	buccal_mucosa	/data/Illumina/buccal_m	9d1d5437b066bc6d	204563138	/data/HMASM/PGAs/bucca	997a90eaa5f9647b26a330	1561875
SRS058336	tongue_dorsum	/data/Illumina/tongue_d	233e4e5d7676699f7	5310085423	/data/HMASM/PGAs/tongu	5086bda04164ff160733711	29681463
SRS015054	posterior_fornix	/data/Illumina/posterior_	42bc0f76c8f9926f8b	422439156	/data/HMASM/PGAs/poste	10657e7673c91765276751	1112796
SRS015168	posterior_fornix	/data/Illumina/posterior_	b015147e2d905ddb	192962347	/data/HMASM/PGAs/poste	d31f26f709f8e4c962b6b9d	714236
SRS014287	stool	/data/Illumina/stool/SRS	caae15eb59387b5be	5206574371	/data/HMASM/PGAs/stool_	9e9107b0474b32522b7b4f	32882141
SRS015794	stool	/data/Illumina/stool/SRS	8364ab71b2d58f6b9	8013438624	/data/HMASM/PGAs/stool_	7519909fc3589656f6640ce	26507858
SRS015278	supragingival_plaque	/data/Illumina/supraging	0584fa81dc5d45c43	2973656473	/data/HMASM/PGAs/supra	89d9a11ac39cd710fe94c9c	23303704
SRS017025	supragingival_plaque	/data/Illumina/supraging	0ff7d4090401492da	2496090651	/data/HMASM/PGAs/supra	a693e951bf460271f8c1aa4	18390932
SRS051930	supragingival_plaque	/data/Illumina/supraging	2e9b5568e2c35c2f1	3801617236	/data/HMASM/PGAs/supra	c03fd66b020b3df8747007e	33147356
SRS018573	supragingival_plaque	/data/Illumina/supraging	ccbfb29197ee4db75	3887461670	/data/HMASM/PGAs/supra	cb07f609d8d86e5536e5d2	24162019
SRS018665	supragingival_plaque	/data/Illumina/supraging	8b3c2aa90bc225838	5372218840	/data/HMASM/PGAs/supra	17f6ddd60c419b12704935	38560668
SRS019387	supragingival_plaque	/data/Illumina/supraging	62019814c63d3860	2359674945	/data/HMASM/PGAs/supra	ef9b15d1e642a6995b08f1a	16953762
SRS015434	tongue_dorsum	/data/Illumina/tongue_d	9b1a3f1b6625af568	7097982670	/data/HMASM/PGAs/tongu	5155dd4c323247eeeffe836	37609631
SRS042643	tongue_dorsum	/data/Illumina/tongue_d	04bf327f2791d146a	7039048097	/data/HMASM/PGAs/tongu	16d59fc148abf028437ad0d	37068835
SRS016319	tongue_dorsum	/data/Illumina/tongue_d	80691ef11220cf321	6557249119	/data/HMASM/PGAs/tongu	84f1088b2de8538e10dc85	29329834
SRS017439	tongue_dorsum	/data/Illumina/tongue_d	75d4d79538d78081	6171025500	/data/HMASM/PGAs/tongu	7b6c47f9b4f33c5d3ff5149a	23786277
SRS017808	tongue_dorsum	/data/Illumina/tongue_d	1369f11bc057a41d0	6233615161	/data/HMASM/PGAs/tongu	457a6c51c6f92bc758310ce	30420086
SRS018357	tongue_dorsum	/data/Illumina/tongue_d	5ca0aacb002c4acd6	5567237535	/data/HMASM/PGAs/tongu	0df9e70ddd2a045094cf5ac	27251883
SRS019025	attached_keratinized_gingiv	/data/Illumina/attached_	78c202438e872919	540733508	/data/HMASM/PGAs/attac	46382fd04105e64eacc9b2	3589812
SRS018971	buccal_mucosa	/data/Illumina/buccal_m	d7dd5bb354ac8d6c	786290980	/data/HMASM/PGAs/bucca	3552ac33f4376db4b19853	6823593
SRS016553	anterior_nares	/data/Illumina/anterior_	61cb5f833df4c923d	47497480	/data/HMASM/PGAs/anter	c1dd105388bd4c5b080f21	172408
SRS018312	anterior_nares	/data/Illumina/anterior_	2454e80d7e5216ad	19820758	/data/HMASM/PGAs/anter	e72a65789d796a09c40257	26017
SRS013945	buccal_mucosa	/data/Illumina/buccal_m	72df171a481e84dc4	2006042200	/data/HMASM/PGAs/bucca	82a3b90f6be3ace7b806e5	10389434
SRS018149	buccal_mucosa	/data/Illumina/buccal_m	f4efe6e0d7215b5ccf	5106745044	/data/HMASM/PGAs/bucca	4a7edf1cc0b194c505301e0	12206144
SRS022625	buccal_mucosa	/data/Illumina/buccal_m	761eac81f6d7db090	1714046653	/data/HMASM/PGAs/bucca	18642303e2045a9769f226	8364054
SRS013155	anterior_nares	/data/Illumina/anterior_	1b667436bfdb2d80	37885237	/data/HMASM/PGAs/anter	c9c7e2793633452ad98097	439757
SRS024377	buccal_mucosa	/data/Illumina/buccal_m	6a37bdaa00b1806a	695972307	/data/HMASM/PGAs/bucca	23e9cb34320bbda17032aa	2060867
SRS051600	anterior_nares	/data/Illumina/anterior_	391775b95926a221	23235970	/data/HMASM/PGAs/anter	7689cf3de28badbd361a69	14187
SRS016191	posterior_fornix	/data/Illumina/posterior_	d25de6c5a2f6a6fe1	503082504	/data/HMASM/PGAs/poste	c9e2715e9f6fc0e0d2b61a0	620787
SRS011144	buccal_mucosa	/data/Illumina/buccal_m	ae781cf1419a6b9b8	233426692	/data/HMASM/PGAs/bucca	6a2a6aa2e3acb77d4b4c08	1620870
SRS013825	buccal_mucosa	/data/Illumina/buccal_m	b56b3f5e6041b6e5c	563436760	/data/HMASM/PGAs/bucca	044c25315da1a10aa4137a	2840603
SRS022545	posterior_fornix	/data/Illumina/posterior_	68893584b0457bb5	12549802	/data/HMASM/PGAs/poste	5fab0771abf2ddca3b18f79	337734
SRS042457	buccal_mucosa	/data/Illumina/buccal_m	790d61cebb442398	361308881	/data/HMASM/PGAs/bucca	40ad045bd0aaee72f4cde31	2663495
SRS043646	buccal_mucosa	/data/Illumina/buccal_m	18dfb30d30cba2a15	294821450	/data/HMASM/PGAs/bucca	489536d81a3a1fab780ddd	1275347
SRS050025	anterior_nares	/data/Illumina/anterior_	ed37f14c4e38b3b40	96553646	/data/HMASM/PGAs/anter	8ca0631b1366208b27f2b3	1474116
SRS013521	stool	/data/Illumina/stool/SRS	2bbfb2bdf0e3b9fa1	7858782296	/data/HMASM/PGAs/stool_	a183eb77e420bf58058045	26424036
SRS011090	buccal_mucosa	/data/Illumina/buccal_m	7f9eebcb529e830f7	275812894	/data/HMASM/PGAs/bucca	f3fe3cf7884acb2fdd08e89c	1574183
SRS016752	anterior_nares	/data/Illumina/anterior_	50434f01bc60ee1b9	85943169	/data/HMASM/PGAs/anter	8b4b59583f7d7a00a3d8cb	1516484
SRS019339	anterior_nares	/data/Illumina/anterior_	76a621d6503d11d1	24213625	/data/HMASM/PGAs/anter	406a45a1508b07b0279c40	58712
SRS023212	anterior_nares	/data/Illumina/anterior_	985021002e87a439	114193279	/data/HMASM/PGAs/anter	20cab25d593e3cfcf539cbe	113008
SRS024064	anterior_nares	/data/Illumina/anterior_	ec385958cc49b687d	57024050	/data/HMASM/PGAs/anter	74b4a3f4e5169ce24be80a3	282731
SRS019073	buccal_mucosa	/data/Illumina/buccal_m	4aa59b8f9bc6a79f6	2405397030	/data/HMASM/PGAs/bucca	9a14c89d398e90a20d871d	13956179
SRS019221	buccal_mucosa	/data/Illumina/buccal_m	be262cbe24f68ab95	218664828	/data/HMASM/PGAs/bucca	6b4d1e149d9be23633f68c	2036455
SRS058105	buccal_mucosa	/data/Illumina/buccal_m	32f5b9e3786e70c62	1597105423	/data/HMASM/PGAs/bucca	b1e568537ce52ebb5004bb	3656907
SRS011061	stool	/data/Illumina/stool/SRS	4a03cd1a16a502ab	6051841119	/data/HMASM/PGAs/stool_	fa5942b5669aa360c22c6cf	26014986

Table S2. Dataset with the topmost OC-affected bacteria (increased and decreased) showing their distribution in human organs compared with the same classification database (minikraken) of 690 samples that passed quality control from the Human Microbiome Project. Three body site samples (attached keratinized gingiva, vaginal introitus)discarded due to low sample numbers, and mid vagina.

SRS ID: A spatial reference system ID in the Human Microbiome Project. Each number is correlated with a site (organ) in the human body that contains multiple types of bacteria. Here we determined the abundance of the most affected bacteria (Up and Down) at each of the organ sites compared to the other bacteria in the same site.

	Increase										Decrease									
Kingdom	Bacteria	Bacteria	Bacteria	Bacteria	Bacteria	Bacteria	Bacteria	Bacteria	Bacteria	Bacteria	Bacteria	Bacteria	Bacteria	Bacteria	Bacteria	Bacteria	Bacteria	Bacteria	Bacteria	Bacteria
Phylum	Firmicutes	Actinobact	Actinobact	Tenericute	Proteobact	Proteobacteria	Caldiserica	Proteobacteri	Firmicutes	Firmicutes	Bacteroidetes	Bacteroidetes	Bacteroidetes	Bacteroidetes	Bacteroidetes	Bacteroidetes	Bacteroidetes	Bacteroidetes	Bacteroidetes	Bacteroidetes
Class	Bacilli	Actinobact	Actinobact	Mollicutes	Gammapro	Deltaproteobacteria	Caldisericia	Gammaprote	Clostridia	Clostridia	Bacteroidia	Bacteroidia	Bacteroidia	Bacteroidia	Bacteroidia	Bacteroidia	Bacteroidia	Bacteroidia	Bacteroidia	Cytophagia
Order	Bacillales	Corynebac	Corynebac	Mycoplasm	Pasteurella	NA	Caldisericales	Oceanospirilla	Clostridiales	Clostridiales	Bacteroidales	Bacteroidales	Bacteroidales	Bacteroidales	Bacteroidales	Bacteroidales	Bacteroidales	Bacteroidales	Bacteroidales	Cytophagales
Family	NA	Corynebac	Mycobacte	Mycoplasm	Pasteurella	Candidatus Desulfofervidaceae	Caldisericaceae	Halomonadac	Clostridiaceae	Clostridiaceae	Bacteroidacea	Tannerellacea	Tannerellacea	Prevotellacea	Prevotellacea	Prevotellacea	Barnesiellacea	Porphyromon	Dysgonamona	Hymenobacteraceae
Genus	Exiguobact	Corynebac	Mycobacte	Mycoplasm	Pasteurella	Candidatus Desulfofervidus	Caldisericum	Halomonas	Candidatus Ar	Candidatus Ar	Bacteroides	Parabacteroid	Tannerella	Prevotella	Prevotella	Prevotella	Barnesiella	Porphyromon	Proteiniphilun	Rufibacter
Species	oxidotolera	ulcerans	immunoge	arginini	multocida	auxilii	exile	sp. N3-2A	sp. SFB-mouse	sp. SFB-mouse	heparinolyticu	sp. CT06	sp. oral taxon	denticola	ruminicola	dentalis	viscericola	gingivalis	saccharoferm	sp. DG15C
SRS011061	4	14	4	24	27	56	20	0	0	1	3808	234392	2769	1370	1074	1023	11753	1507	1367	87
SRS011090	0	1	1	0	159	1	0	0	0	0	4	0	24	33	2	2	5	53	1	1
SRS011098	16	1713	90	4	690	8	6	15	0	0	178	2	400	1143	103	204	67	942	32	27
SRS011126	84	3303	214	22	3407	47	43	72	3	3	593	129	175077	2452	614	1500	875	10673	330	109
SRS011132	0	13	1	0	19	1	1	0	0	0	0	0	1	1	1	0	0	2	0	0
SRS011134	75	89	26	428	224	46	280	4	3	4	50214	598769	3462	8350	19835	10579	18936	6921	2356	270
SRS011140	100	53	61	29	9256	31	68	1	60	8	623	64	6127	10343	675	999	584	5383	202	121
SRS011144	0	2	3	0	401	1	1	0	1	0	35	17	451	184	12	27	13	138	6	1
SRS011152	30	1265	66	18	5634	12	15	11	1	1	469	61	37115	2560	522	1017	559	3801	185	19
SRS011239	33	29	70	66	143	14	87	17	7	5	96269	1853839	4300	4957	5367	6200	14324	4715	2273	91
SRS011243	70	66	2	1	22097	11	14	2	76	0	699	139	2257	7547	322	556	383	3420	108	41
SRS011247	3	40	1	3	2516	22	3	1	8	0	176	229	913	3513	127	198	183	1222	38	5
SRS011255	10	602	65	72	395	4	18	25	0	0	1084	124	11043	155341	940	3454	421	11490	137	46
SRS011263	0	10	2	0	16	0	1	0	0	0	0	27	0	3	0	0	1	0	0	0
SRS011269	0	0	0	0	9	1	0	0	0	0	36	11	0	112	22	156	4	41	1	0
SRS011271	42	41	28	113	882	28	53	24	1	10	24558	170620	3805	51440	138748	52242	19213	7269	5145	426
SRS011302	28	81	18	0	273	22	32	9	6	0	29320	34365	1420	1355	1756	1625	46843	837	1331	195
SRS011306	54	67	79	30	12712	54	104	7	10	12	2778	112	10904	56696	2462	5274	1916	14157	535	24
SRS011310	3	2	0	2	2479	6	5	0	0	0	51	10	4446	1461	122	168	111	1365	44	2
SRS011343	11	907	55	18	8015	18	6	2	1	3	759	39	29324	2128	416	890	525	4329	154	31
SRS011355	1	0	0	0	6	0	1	0	0	2	1	0	0	4	1	0	0	3	1	0
SRS011397	1	46	3	0	30	0	0	1	0	0	10	16	13	23	15	12	11	102	3	1
SRS011405	4	18	29	6	160	14	26	0	0	2	132731	58975	2256	2841	3770	4258	16986	3140	3048	171
SRS011452	1	3	2	0	18	2	5	2	0	0	21054	59604	867	919	888	1582	2903	1133	705	39
SRS011529	64	486	4	19	337	51	28	2	7	0	42559	82404	5542	44076	103898	47141	18898	8840	3328	548
SRS011584	0	0	0	0	3	0	0	0	0	0	3	7	1	0	0	0	2	0	0	0
SRS011586	16	54	9	255	117	11	168	19	1	25	50317	308213	1034	1996	2038	4359	25878	2427	1941	91
SRS012273	35	177	10	94	324	138	80	7	25	3	21588	186874	2520	2502	1055	2053	23208	1331	952	66
SRS012279	247	99	186	9	5368	31	45	8	56	5	5644	380	272966	44086	2694	7056	1598	20126	472	76
SRS012281	0	0	2	0	55	0	0	0	0	0	5	0	651	38	4	17	12	49	1	0
SRS012285	54	2104	260	10	4169	13	6	12	0	2	1239	76	16409	6061	735	1846	790	8392	190	57
SRS012291	0	10	2	0	5	0	0	0	0	0	0	1	0	1	0	0	1	0	1	0
SRS012294	0	0	0	0	9	0	0	0	0	0	0	0	0	0	0	0	0	0	0	0
SRS012663	23	77	19	0	29	0	0	0	0	0	0	1	0	1	0	1	0	0	0	0
SRS012902	11	1	0	0	141	9	0	0	0	0	3173	164813	342	37	73	240	41085	1932	1104	41
SRS013155	1	20	2	0	6	0	0	0	0	0	8	66	13	57	0	7	11	12	0	0
SRS013158	55	39	7	1	45	0	5	0	0	1	36983	202463	4633	2082	1087	1863	7410	9978	848	63
SRS013164	55	97	57	4	11702	12	93	3	20	0	3229	166	20315	14093	2833	4224	3757	42095	731	204
SRS013170	35	2612	147	31	8859	50	13	24	0	6	1078	187	173367	14500	1815	4295	1249	16766	533	94
SRS013215	4	6	0	0	62	0	0	38	0	0	3077	111273	178	628	548	134	3627	319	952	5
SRS013234	211	50	7	2	11472	6	78	1	77	1	1157	11	5688	26219	1181	2441	114	2184	228	39
SRS013239	6	16	0	4	1581	5	8	1	0	0	263	9	2875	1730	358	236	178	285	39	49
SRS013252	65	6290	200	63	2884	32	33	19	1	0	1042	111	1048501	9596	670	2235	561	5674	271	77
SRS013258	1	13	3	3	32	6	1	3	0	0	2	15	34	5	2	4	3	2	0	30
SRS013269	0	12	2	0	7	0	0	0	0	0	0	0	1	0	0	0	0	1	0	0
SRS013476	26	31	10	18	249	2	51	14	0	0	20699	26527	1804	1521	1338	1630	17673	1428	728	24
SRS013502	31	43	62	29	29275	3	15	1	24	0	147	43	701	9522	196	263	167	1799	60	26
SRS013506	3	49	0	15	4267	4	0	59	0	52	565	6	392	1688	1143	750	1068	3703	41	84
SRS013521	11	17	3	2	251	14	3	3	0	1	18129	38381	7459	41053	103902	30145	46967	4652	5565	755
SRS013533	36	2321	229	102	670	28	36	23	2	7	1082	64	61494	37407	935	5786	551	8577	198	64
SRS013542	15	0	1	5	10	1	0	0	0	0	419	8	9	1739	173	647	121	320	5	1
SRS013637	0	8	1	0	8	0	0	0	0	0	1	3	2	2	2	0	0	1	0	0
SRS013687	104	387	8	14	552	11	128	9	7	1	38000	43509	4791	35469	78331	31695	19379	6962	5806	257
SRS013705	74	178	74	73	3614	66	75	2	33	8	4883	73	110421	55786	2639	6001	1676	15001	533	49
SRS013711	4	3	1	7	360	5	3	0	2	0	318	39	17237	6240	271	568	152	1782	55	4
SRS013723	99	4109	281	13	3948	32	31	450	5	1	1930	191	416518	46310	1795	4512	1970	18922	576	118
SRS013800	70	8	1	3	184	0	16	54	2	7	66291	917616	5473	6099	8256	9187	49976	4651	4590	285
SRS013818	115	24	61	2	5875	11	43	6	26	4	493	757	3028	31777	497	1189	204	11899	66	36
SRS013825	3	15	5	4	793	3	8	1	0	0	115	801	826	4500	31	510	56	9166	7	2
SRS013836	63	541	33	31	628	14	47	8	0	16	2677	164	117486	527040	1465	8635	656	20151	202	36

SRS013876	0	628	35	0	5	0	0	0	0	0	11	24	1	8	10	4	8	5	0	0
SRS013879	139	45	42	2	8466	37	32	11	41	2	23106	108	24960	17922	3197	6475	4695	27511	632	269
SRS013881	5	1	5	1	1396	7	0	3	0	18	424	5	2050	723	104	190	208	1267	40	8
SRS013942	14	10	2	15	1883	2	19	2	3	1	508	59	1503	2424	236	2174	252	2361	47	15
SRS013945	4	17	23	9	11031	18	91	14	2	3	326	7	1115	376	97	318	151	569	42	17
SRS013946	1	211	18	75	4820	10	73	6	0	5	7063	770	5614	17414	8507	5474	4706	31501	786	1277
SRS013947	13	5	24	12	6249	28	22	1	11	0	2127	96	6718	3757	866	1629	1482	6454	284	41
SRS013948	40	19	20	15	1868	10	5	2	12	0	4412	59	8216	5635	1003	2085	1495	5617	242	78
SRS013949	13	1971	124	22	14731	4	45	8	0	0	533	19	3702	1215	272	455	250	2342	141	28
SRS013950	2	218	18	62	1060	3	31	4	0	0	1239	94	12095	116624	1604	8509	588	10360	136	62
SRS013951	26	257	26	537	128	182	61	6	3	7	21729	41908	966	4926	2110	5321	4345	1458	451	30
SRS013956	0	6	0	0	2	0	0	0	0	0	0	0	1	1	0	0	0	0	0	0
SRS014107	4	121	3	6	149	4	0	5	0	0	447	265	30489	6355	352	1694	282	5377	69	20
SRS014124	155	104	132	2	16609	10	40	19	111	9	250	47	2182	15665	430	546	440	5028	191	13
SRS011063	223958	65058	83262	2094	747	1621989	693075	2014541	49118	1508644	28113	2025876	712710	28129	839	52227	397865	837	1642647	1379909
SRS014126	1	2	1	0	219	11	0	0	0	0	9	125	41	56	4	5	12	37	3	0
SRS014235	103	69	16	104	284	17	65	11	1	2	63587	186861	3995	2182	2605	2216	25302	2367	2466	57
SRS014271	104	125	45	7	1431	31	69	15	23	12	3490	47	257478	44592	3314	9159	1532	10334	318	98
SRS014287	26	12	12	4	39	15	58	5	5	1	6931	154087	1382	383	439	272	5888	420	443	43
SRS014313	2	20	3	1	43	3	6	4	0	1	19432	2224868	700	931	834	859	9885	429	1141	6
SRS014459	53	72	7	3	81	6	14	96	0	7	36549	612517	2668	4098	1725	1881	12538	1595	1573	127
SRS014464	0	50	0	0	3	0	0	2	0	0	1	0	77	13	0	2	0	1	0	0
SRS014465	0	0	1	0	9	0	0	0	0	0	0	0	0	1	0	2	0	0	0	0
SRS014466	0	0	12	0	3	0	0	0	0	0	2	3	3	12	1	12	0	2	0	0
SRS014470	90	161	132	13	1475	106	45	4	95	10	2409	23	17254	58150	2928	6109	685	4538	419	53
SRS014472	1	8	1	1	126	4	0	0	0	0	18	1	871	644	10	46	5	124	0	0
SRS014473	4	137	7	15	6497	1	3	5	1	1	6449	223	2643	2353	1866	764	1060	10353	519	264
SRS014474	2	2	1	1	203	4	0	0	10	0	147	2	1077	2561	125	425	45	640	18	3
SRS014475	0	1	1	0	48	1	0	0	2	0	16	5	209	372	19	40	8	33	2	1
SRS014476	23	2123	130	54	928	9	15	15	1	3	486	24	14398	13657	571	1630	458	8205	236	52
SRS014477	12	87	9	20	123	7	4	3	0	1	227	32	1618	4021	159	790	135	3948	59	15
SRS014494	0	0	2	0	41	2	0	0	0	0	147	382	24	12	24	15	73	7	0	0
SRS014573	161	179	28	35	6083	70	70	17	38	11	735	107	224057	10684	1544	2460	1123	8038	360	47
SRS014575	0	9	0	0	737	0	0	5	0	0	22	2	1752	97	19	25	28	179	11	1
SRS014578	36	1151	148	143	1180	8	30	67	0	1	130	44	25265	1671	518	786	585	7975	255	18
SRS014613	43	52	14	2	210	0	14	3	0	1	95551	83016	6341	2035	2833	2849	38329	2395	1884	321
SRS014629	0	0	3	0	6	0	0	0	0	0	3	1	0	3	1	2	0	1	0	0
SRS014682	1	1901	31	1	12	1	2	1	0	0	0	0	2	5	2	4	16	9	54	0
SRS014683	15	211	8	0	63	3	4	114	2	0	83242	180357	5063	2731	3160	2085	26103	3719	2413	262
SRS014684	184	218	86	6	1221	42	61	9	254	6	3753	6	3652	54448	1491	5044	1285	6808	94	117
SRS014686	6	10	5	5	228	1	4	0	3	0	104	5	1478	2627	31	676	44	394	7	7
SRS014687	12	3	0	118	2390	30	39	2	1	0	6	0	12	54	2	8	3	94	2	2
SRS014689	23	49	33	2	160	4	25	1	19	2	1023	9	1570	15433	647	2010	470	2240	49	30
SRS014690	3	302	6	10	2688	5	15	3	1	0	176	8	17563	7548	259	639	290	2089	67	50
SRS014691	13	67	4	285	78	1	19	29	2	0	1288	73	5654	42917	1198	10023	576	20036	228	108
SRS014888	87	107	59	7	15455	63	40	3	39	1	2906	75	18938	3861	1222	1848	1898	12249	362	113
SRS014890	0	19	3	0	163	0	0	0	0	0	16	1	2142	52	13	26	24	99	3	1
SRS014894	6	1221	114	2	2455	0	0	31	0	0	39	2	1775	265	38	52	128	847	27	12
SRS014901	1	130	13	0	16	0	0	2	0	0	0	0	10	16	3	0	1	14	8	0
SRS014923	36	271	21	21	159	11	82	10	41	0	29676	48458	1694	1396	1176	2751	8842	967	835	241
SRS014979	40	92	5	14	76	10	29	30	26	1	38283	57929	3096	1291	1058	1348	12084	1631	452	112
SRS015038	74	98	67	15	699	10	23	4	23	58	1952	11	1422	24440	914	2053	182	2514	127	27
SRS015040	2	1	0	2	750	14	0	0	2	6	19	4	857	453	16	52	14	131	9	2
SRS015044	12	2020	117	37	3737	12	11	5	1	3	358	68	116091	5711	610	1278	557	6540	179	36
SRS015051	3	0	0	0	14	0	0	0	0	0	1	1	7	1	0	0	0	0	0	0
SRS015054	0	2	90	1	4	0	0	0	0	0	0	0	0	0	0	0	0	0	0	0
SRS015055	18	28	14	8	332	6	0	4	3	1	252	12	3079	4192	263	1590	63	1004	32	11
SRS015057	170	237	166	37	725	80	98	4	60	31	3082	16	6620	82860	4115	7811	323	4174	769	29
SRS015059	1	9	2	0	24	0	0	0	0	0	12	0	2885	177	8	29	4	55	0	0
SRS015060	1	155	34	3	3834	1	4	8	1	1	3272	151	1338	1139	886	383	534	4404	260	143
SRS015061	6	3	2	3	65	0	3	5	1	0	465	22	140	8891	163	1430	37	3257	20	4
SRS015062	1	1	6	0	35	1	0	0	0	0	104	11	103	1997	55	331	12	727	2	0
SRS015063	58	2354	223	0	382	0	0	12	0	0	27	4	828	298	33	86	74	708	20	14
SRS015065	43	63	9	25	143	3	12	37	3	6	34006	323692	2490	3939	1572	1614	11657	1497	1524	81
SRS015071	0	2	8	0	15	0	0	0	0	0	0	6	3	4	1	2	1	1	1	0
SRS015072	1	0	11	0	11	1	0	0	0	0	0	2	1	1	0	3	1	0	1	0
SRS015073	0	0	0	0	22	0	0	0	0	0	2	3	0	0	0	0	0	0	0	0
SRS015133	50	100	8	6	190	30	94	1	146	1	16354	58141	2230	2018	4733	3660	10594	3652	1698	315
SRS015154	3	34	1	0	1396	7	3	0	1	6	35	8	5694	510	37	75	48	311	20	0
SRS015158	6	2496	170	60	2709	0	6	3	3	1	78	11	1260	198	101	53	56	1085	74	27
SRS015168	0	0	1	0	13	0	0	0	0	0	8	2	1	12	3	12	0	4	0	0
SRS015190	15	31	8	6	32	15	30	3	0	0	58052	682446	2926	2155	1120	1115	10032	6878	442	12
SRS015209	16	701	169	3	701	49	40	5	21	76	2939	133	70437	69621	3147	7491	1607	10426	451	97
SRS015215	11	1300	92	44	1441	7	13	17	0	8	1323	62	71876	25411	2592	8056	984	14488	241	70
SRS015217	67	414	26	425	60	18	7	34	0	0	21702	95697	1294	1292	659	1135	3594	1097	614	140
SRS015225	0	0	12	0	13	1	0	0	0	0	4	4	2	6	1	0	0	3	0	0
SRS015264	22	10	8	4	62	8	4	3	4	1	35067	171638	977	1805	1253	1379	29850	1395	1194	39

SRS015269	4	13	1	0	13	1	0	0	0	0	2	28	1	1	1	0	5	1	0	1
SRS015272	162	112	19	2	423	137	70	5	24	86	1209	37	1949	30867	2081	2607	261	3062	247	60
SRS015274	3	4	2	0	189	0	0	0	0	1	8	5	710	202	7	21	2	39	1	0
SRS015278	7	2713	140	12	185	6	1	3	0	3	89	52	8923	1138	97	228	89	633	42	15
SRS015369	44	5	0	1	13	0	0	5	0	0	48093	259901	2141	1356	1539	998	28010	863	1712	16
SRS015374	2	8	1	7	1469	4	0	0	1	0	3	1	157	50	1	9	3	27	2	0
SRS015378	3	1376	56	3	1485	2	1	1	0	0	7	3	116	80	2	3	5	34	4	8
SRS015381	0	16	0	0	7	0	0	0	0	0	0	1	36	4	0	141	27	14	0	0
SRS015395	138	68	33	0	875	4	21	8	96	0	234	108	1591	8546	320	893	96	2498	90	19
SRS015425	0	0	1	0	13	0	0	0	0	0	0	0	0	0	0	0	0	0	0	0
SRS015430	0	27	7	0	23	1	1	0	0	0	0	0	2	1	0	0	0	0	0	0
SRS015434	107	250	85	69	6364	128	95	6	66	12	2072	49	389414	16841	1740	3326	1448	11014	501	63
SRS015436	5	8	3	10	7232	17	5	1	1	15	137	18	4269	1269	132	141	112	1460	43	4
SRS015440	8	1563	197	3	2547	3	1	35	0	1	170	37	4491	976	218	296	314	2085	95	20
SRS015450	0	9	0	1	4	0	0	0	0	0	1	11	2	0	0	0	0	0	0	0
SRS015470	5	1920	234	1	4031	10	2	29	0	0	224	137	5147	1442	270	390	358	2491	128	27
SRS015537	69	97	1	4	326	3	8	20	27	0	210	4	4328	12413	223	471	18	3517	23	19
SRS015574	32	2258	184	12	1509	59	5	32	0	15	757	80	175977	66044	536	1715	478	4743	113	30
SRS015578	55	27	31	30	535	9	43	18	1	1	53266	70944	302	775	1707	6022	4618	507	522	54
SRS015640	0	11	1	0	3	0	0	0	0	0	0	0	0	4	0	0	1	2	1	0
SRS015644	100	69	52	2	8196	12	58	7	19	3	502	9	2792	11545	229	837	149	1939	97	119
SRS015646	9	1	0	2	1508	1	0	2	0	0	22	0	276	157	14	29	6	463	5	4
SRS015650	8	2046	35	12	578	1	0	3	0	0	96	10	1090	215	57	293	110	83930	101	1
SRS015663	175	117	2	303	264	44	67	9	5	1	44070	296560	3592	8724	14906	12309	115451	6050	4705	957
SRS015745	4	40	14	4	1517	3	2	3	2	1	245	5	22516	802	127	241	124	902	23	12
SRS015752	10	142	13	0	8	0	0	0	0	0	4	3	3	4	0	3	1	6	0	0
SRS015755	1	896	109	5	1738	3	2	2	0	1	267	9	3687	1125	186	177	176	1803	67	23
SRS015762	71	63	49	24	6216	96	128	4	24	12	7453	84	149028	39303	4441	10500	3556	23334	832	166
SRS015782	48	51	8	129	71	13	102	5	1	0	36137	105430	4690	12473	15606	12632	12679	5508	2660	215
SRS015793	0	79	2	0	6	0	0	0	0	0	2	0	0	2	0	1	1	2	0	0
SRS015794	9	59	2	8	190	3	2	1	0	0	36083	205193	4867	19610	40524	24690	145964	9777	4097	548
SRS015797	145	42	37	15	6208	41	34	10	182	0	10577	68	24953	27574	2558	4912	3822	29946	918	74
SRS015799	1	1	0	3	2322	4	0	3	0	5	132	9	319	91	31	40	45	304	10	10
SRS015803	7	443	21	11	4134	6	4	8	0	0	930	20	3836	2089	420	661	302	5806	203	75
SRS015854	10	77	21	23	256	23	64	14	0	2	34754	59571	1046	1238	947	1222	7864	969	796	49
SRS015893	45	274	36	1	654	19	22	6	8	1	1082	40	183577	36037	1993	3842	314	9816	303	75
SRS015895	4	10	2	0	1150	0	3	4	1	9	4	0	249	253	11	14	5	61	1	2
SRS015899	10	632	105	6	1430	0	10	30	0	0	125	41	1949	604	251	227	149	1934	43	30
SRS015921	3	9	7	0	991	1	12	0	2	2	7	11	365	272	15	7	7	67	4	2
SRS015937	19	396	16	0	108	0	1	0	0	0	0	0	3	3	0	5	0	10	5	0
SRS015941	204	191	78	5	4304	43	46	9	66	4	669	40	3705	23257	1162	2430	397	6400	188	78
SRS015947	13	133	49	4	144	0	1	0	2	0	11	26	489	138	21	49	16	141	3	9
SRS015960	8	68	3	7	93	12	18	24	0	1	64669	53136	4840	1900	1954	2625	76675	3434	2108	141
SRS015989	12	2207	14	7	3142	1	3	8	0	0	148	15	644	664	135	215	140	6705	84	19
SRS015996	6	695	57	0	13	0	0	0	0	0	0	0	11	3	0	0	1	6	0	9
SRS016002	71	187	85	48	24998	53	79	4	25	6	2379	45	8193	27677	1502	3360	1948	12439	421	119
SRS016018	13	70	4	10	84	1	10	0	0	0	96896	124620	6752	5377	4581	5252	89408	4250	3300	279
SRS016033	0	1	0	0	7	0	0	0	0	0	1	3	2	1	1	0	1	3	0	0
SRS016037	57	112	16	26	14374	165	70	12	39	0	3273	190	20894	15689	2527	4628	3030	23511	764	247
SRS016039	0	21	13	1	1883	2	1	1	0	0	36	5	3461	170	11	33	18	132	5	1
SRS016043	16	1010	87	3	2294	12	10	10	0	1	494	10	25415	3379	491	836	358	5053	143	36
SRS016056	82	374	2	1	65	0	6	17	0	0	223980	232904	5690	5669	6983	6318	35670	4774	5341	493
SRS016086	49	107	10	1	9245	8	9	3	45	8	277	46	782	15404	480	1440	209	2803	134	5
SRS016088	0	2	0	0	116	0	0	0	0	0	5	0	35	62	1	6	5	26	0	0
SRS016092	3	207	79	0	3727	2	0	39	0	0	66	6	340	59	14	33	34	344	19	36
SRS016095	369	88	5	2	192	18	70	118	3	0	17862	664975	3997	3631	2659	2924	83848	4772	3642	466
SRS016111	0	0	0	0	12	0	0	0	0	0	1	4	0	0	0	0	3	0	0	0
SRS016188	0	5	0	1	13	0	0	0	0	0	1	2	1	8	1	4	1	1	2	0
SRS016191	0	2	13	0	12	1	0	0	0	0	5	4	6	11	2	16	0	4	0	0
SRS016196	0	4	2	4	339	2	2	3	0	0	8	1	42	31	8	2	8	84	0	0
SRS016200	17	3008	287	5	1369	10	9	15	0	2	46	9	606	620	42	107	172	1432	26	32
SRS016203	9	14	11	10	54	3	19	0	1	0	17034	16355	1073	1410	759	2090	5799	846	338	31
SRS016225	101	75	47	21	15093	14	51	17	82	1	4521	74	24297	29521	3019	4329	3592	32791	729	150
SRS016267	8	9	4	2	28	10	10	4	0	0	29072	1348135	2083	2019	1282	1083	21905	2112	1237	226
SRS016292	0	313	9	0	8	0	0	0	0	0	0	0	1	3	1	1	0	1	0	0
SRS016297	1	21	4	3	302	0	2	0	0	0	20	1	10346	258	19	44	13	206	7	2
SRS016319	155	273	90	92	1388	80	56	12	38	4	812	418	9987	19782	1260	3721	314	3465	156	28
SRS016331	14	2005	89	30	364	14	22	10	0	1	1133	519	32879	2835	339	1617	472	6216	195	26
SRS016335	851	64	44	452	211	26	144	12	5	5	45501	95899	2876	1820	2964	2456	29368	2199	1787	110
SRS016342	121	53	34	3	11161	42	26	19	42	2	2045	35	4307	43056	1453	3407	250	4392	300	23
SRS016349	3	4	2	2	315	4	0	0	0	1	1658	1358	622	1926	91	224	691	246	99	0
SRS016360	21	1263	47	73	4720	5	2	9	1	4	185	97	47118	3110	419	848	242	2432	197	225
SRS016434	0	14	4	0	7	0	0	0	0	0	5	8	0	7	0	4	7	1	1	1
SRS016495	11	88	20	5	44	3	149	3	1	0	15836	43761	280	225	342	376	4205	311	178	13
SRS016501	88	11	4	1	14895	0	24	21	124	0	64	8	633	845	41	186	42	604	85	165
SRS016503	14	2	3	2	513	1	0	1	1	0	3	2	60	29	2	12	1	26	3	1
SRS016513	0	0	0	0	9	0	0	1	0	0	0	0	0	1	0	0	0	0	0	0

SRS016516	0	0	0	0	4	0	0	0	0	0	0	0	0	0	0	0	0	0	0	0
SRS016529	71	12	4	1	16334	2	20	66	116	0	58	13	685	845	49	211	46	568	90	123
SRS016533	20	7	7	1	662	1	0	4	2	0	5	3	79	55	5	23	1	42	0	2
SRS016553	0	1	0	0	23	0	0	1	0	0	3	9	0	25	0	3	4	2	0	0
SRS016559	0	0	0	0	12	0	0	0	0	0	0	1	0	0	0	0	0	0	0	0
SRS016569	176	156	72	24	7643	51	66	0	35	2	850	154	18602	26542	1422	3209	1377	10466	444	55
SRS016575	29	1502	147	96	6612	8	15	14	1	4	644	187	216588	87463	1357	5465	1048	10498	274	74
SRS016581	0	3	0	0	8	1	1	0	0	0	2	2	0	0	0	0	0	0	0	0
SRS016585	2	6	0	3	8	1	3	1	3	0	3232	11337	80	153	225	296	1723	110	142	30
SRS016600	2	2	1	2	987	23	3	3	1	0	132	386	1661	776	37	54	113	531	22	1
SRS016746	21	3805	144	46	4477	7	9	9	1	0	1022	69	28033	7848	693	1366	445	9173	123	75
SRS016752	0	81	3	0	9	0	0	0	0	0	4	1	1	4	0	7	1	7	1	0
SRS016753	8	25	0	1	132	2	10	53	1	0	34295	136624	4915	3580	2207	1208	8324	6019	2709	50
SRS016944	0	35	2	0	23	1	4	1	0	0	26	17	48	17	6	15	9	14	2	0
SRS016954	38	53	5	29	91	102	37	1	0	0	153159	133646	5926	3872	4905	4032	59221	4614	9225	202
SRS016989	28	13	8	0	87	4	2	17	29	0	65771	157496	1461	2715	2636	2898	63801	1962	2183	50
SRS017013	25	10	1	1	3509	16	2	1	1	94	73	28	1486	978	97	189	114	1030	53	20
SRS017025	7	836	65	3	4102	13	2	13	0	1	65	10	633	836	38	98	56	591	25	34
SRS017044	5	57	2	0	32	0	1	0	0	0	3	7	11	7	2	1	5	1	0	4
SRS017080	7	35	3	1	563	2	4	1	3	0	79	27	1896	2289	95	219	29	258	22	1
SRS017103	19	301	5	5	104	5	47	91	14	2	130350	376477	5960	6002	5138	4671	69238	5358	29061	421
SRS017120	244	457	97	29	2320	11	68	28	116	14	1386	17	43732	45597	2432	5124	1057	4821	351	43
SRS017127	2	24	1	0	721	5	0	39	0	0	397	23	466	1144	426	158	391	11483	207	13
SRS017139	41	6083	216	107	3788	6	5	23	5	0	591	153	22713	27987	1159	1396	1734	13209	594	45
SRS017156	2	15	1	0	13	0	0	0	0	0	2	13	4	7	0	1	2	2	0	1
SRS017191	17	9	2	6	57	1	81	2	3	0	3353	167138	1438	1229	632	765	19948	972	914	28
SRS017209	38	43	16	38	10193	48	16	4	116	3	1961	50	51389	8854	1411	2757	2238	17880	422	122
SRS017215	14	8	58	30	4124	27	3	16	5	2	139	19	3441	1263	198	233	203	1997	45	12
SRS017227	38	1958	166	57	6231	22	27	94	6	9	2532	400	100310	39514	2283	9628	1445	26124	587	171
SRS017244	0	2	0	0	4	0	0	0	0	0	0	0	1	1	0	1	1	1	1	0
SRS017247	2	59	1	0	84	158	4	3	1	0	22995	74704	1716	1284	1091	932	22044	1364	2271	64
SRS017304	5	1734	107	7	1908	9	5	14	0	0	638	9	248407	1370	102	391	64	1416	18	12
SRS017307	74	44	19	97	234	6	15	29	1	0	21769	50762	2736	59450	171425	73004	82318	9926	5948	886
SRS017433	19	109	1	19	127	2	4	0	1	0	129620	120421	6133	7159	9617	10283	55320	3543	4397	722
SRS017439	123	137	45	7	16286	130	19	14	45	0	2033	123	13896	16355	2207	3264	2610	21157	827	91
SRS017441	2	12	1	2	612	1	0	0	0	0	63	5	1097	586	104	119	93	625	14	8
SRS017445	16	2204	253	4	25861	15	18	44	0	0	168	42	9432	767	190	294	222	3055	128	147
SRS017451	2	142	7	0	42	0	0	0	0	0	11	1	5	8	3	5	3	22	3	1
SRS017497	61	2	0	0	17	1	0	0	0	0	1885	34	46	5047	310	1081	306	2193	5	3
SRS017511	34	2922	162	13	2048	37	29	18	0	10	841	101	205559	6589	914	2243	800	12497	263	43
SRS017520	0	0	0	0	6	0	0	0	0	0	1	0	1	1	0	0	0	1	0	0
SRS017521	83	131	25	6	137	19	10	5	66	1	57722	223862	4218	3263	2506	2408	38536	3023	2863	205
SRS017533	122	194	132	14	9358	55	54	12	117	1	2451	43	5547	19052	1060	2363	987	10725	241	118
SRS017537	1	5	1	2	177	0	0	2	0	0	17	3	217	457	10	62	15	175	4	1
SRS017687	0	35	2	1	678	8	1	1	0	0	110	65	2128	7193	87	852	75	950	21	3
SRS017691	28	1607	175	96	3384	9	22	11	2	17	1973	169	112832	173015	2134	9626	1391	17873	418	44
SRS017697	0	55	3	0	4	0	0	0	0	0	11	17	18	54	0	4	4	3	0	0
SRS017700	0	0	0	0	6	2	0	0	0	0	4	6	6	10	0	4	1	3	0	0
SRS017701	15	14	10	3	168	7	15	2	1	0	119680	260541	3835	4706	5491	14374	30488	3961	3269	159
SRS017713	57	347	24	6	6780	55	13	8	7	6	339	87	2803	18196	446	1141	106	2451	95	24
SRS017808	104	170	63	8	14318	26	37	1	13	1	2981	58	10530	53960	1787	4335	777	7904	224	37
SRS017810	2	13	5	5	1386	10	3	2	0	6	403	29	4734	12358	342	840	243	2692	69	15
SRS017814	8	1216	41	2	3096	6	1	8	0	0	87	11	1288	312	66	67	135	1880	52	9
SRS017820	13	83	13	0	13	0	2	1	0	0	1	0	3	2	4	1	1	8	5	0
SRS017821	68	31	18	8	205	6	15	5	4	0	98900	40929	3117	3600	4494	4432	45284	4635	3940	254
SRS017849	5	130	28	3	52	1	1	55	0	0	517	686	64	688	641	322	279	159	44	227
SRS017851	2	103	21	0	36	1	2	24	0	0	172	84	21	27	10	12	87	36	12	183
SRS018133	144	34	2	0	113	2	1	3	71	0	42388	141955	11060	3880	7100	2736	38121	2831	4242	138
SRS018145	55	98	130	6	22251	107	100	5	355	2	8823	180	27655	15935	2995	4739	4586	51985	616	107
SRS018149	13	85	5	22	10875	250	37	82	11	0	602	9	3076	1646	327	320	378	4476	104	10
SRS018157	38	2097	173	42	8364	74	72	64	21	3	1000	124	65675	5648	730	2603	910	15430	442	121
SRS018300	114	100	36	43	5008	69	15	1	11	4	1937	57	321092	50197	2158	5357	617	10601	459	56
SRS018312	0	2	0	0	11	0	0	0	0	0	0	0	0	0	0	0	2	0	2	0
SRS018329	11	9	0	5	1338	5	1	0	1	0	131	61	773	8491	33	145	60	256	5	1
SRS018337	13	327	167	2	2979	2	2	1	1	2	319	6	29434	10580	210	1231	252	1120	20	21
SRS018351	21	40	37	42	140	30	67	2	85	0	9783	104010	2163	1862	807	1927	34278	1093	780	184
SRS018357	76	62	69	3	8121	21	72	6	34	2	6885	70	21626	42587	2846	6318	2970	14890	447	171
SRS018359	3	60	7	3	2478	4	3	0	2	0	99	3	2152	1243	61	161	87	657	14	4
SRS018369	0	8	0	0	8	0	0	0	0	0	0	0	6	5	0	0	1	2	1	0
SRS018394	51	2122	118	13	4793	29	23	30	0	16	1028	146	136936	4098	459	1024	689	6780	182	57
SRS018427	66	60	94	0	146	18	31	2	1	6	117979	86226	2937	4138	3891	8622	16651	2605	1703	54
SRS018439	89	145	211	24	10728	31	62	8	31	1	9086	42	171538	34943	3023	6186	3170	50417	673	69
SRS018463	0	9	1	0	18	0	0	0	0	0	11	0	227	47	2	12	4	63	2	0
SRS018573	6	1125	110	5	4335	2	2	3	1	0	117	13	770	4100	232	699	152	2104	135	113
SRS018575	7	12	5	2	21	6	4	2	2	0	35513	294357	3786	1895	1543	1723	13080	1873	1221	34
SRS018585	5	26	0	0	3	0	0	0	0	0	0	0	0	1	1	0	0	1	0	1
SRS018591	54	552	16	11	335	67	20	3	44	80	1721	40	1938	46392	2473	4620	312	12586	294	107

SRS018656	4	17	3	54	22	7	28	0	0	2	2449	383440	5614	1299	167	2099	9933	797	735	191
SRS018661	2	10	2	0	799	1	1	0	0	0	139	19	3569	1334	69	248	110	626	22	5
SRS018665	44	1547	102	45	4973	8	10	20	12	6	781	267	327516	6602	1126	2400	1764	15826	586	159
SRS018671	0	30	5	0	6	0	0	0	0	0	1	0	3	9	1	2	0	9	0	0
SRS018739	60	129	57	61	1210	50	42	1	9	18	4576	155	514630	32473	2830	7862	1878	15301	527	108
SRS018769	0	0	0	0	17	0	0	0	0	0	6	18	1	4	1	1	2	1	0	0
SRS018778	0	332	43	0	106	0	0	1	0	0	17	3	50	710	14	89	15	140	4	9
SRS018784	0	63	0	0	6	0	0	0	0	0	0	0	2	4	2	0	0	0	0	1
SRS018791	62	266	38	8	2840	17	22	11	35	16	2332	52	4227	93075	3910	10963	598	11381	616	47
SRS018817	144	125	46	9	104	7	13	1	2	0	129604	98998	8000	2776	2704	2580	17827	4215	2609	138
SRS018969	50	157	118	6	2479	4	7	17	14	2	123	31	7351	11579	368	646	190	1924	107	26
SRS018971	19	15	4	0	2634	2	6	2	5	0	29	7	967	465	26	48	41	358	7	5
SRS018975	10	854	234	3	1050	1	7	20	1	1	84	77	13331	452	160	234	178	2609	58	23
SRS018978	0	53	0	5	68	3	14	3	0	0	97	409	60	28	8	33	77	21	0	2
SRS018981	0	22	3	0	6	0	0	0	0	0	0	8	6	2	0	4	1	0	0	0
SRS019015	58	75	1	0	85	45	1	68	0	0	1	19	1	10	1	2	6	6	0	0
SRS019016	47	159	14	0	76	57	0	11	0	0	2	7	3	14	1	11	15	5	0	4
SRS019019	3	205	5	0	4	0	0	0	0	0	0	7	3	2	0	0	1	8	6	0
SRS019022	181	734	520	8	3748	8	28	56	196	2	3204	49	14298	21211	881	3172	1264	6799	228	150
SRS019024	0	13	6	12	149	0	2	0	1	0	86	0	2652	1647	54	732	48	465	5	4
SRS019025	6	26	0	6	234	0	5	0	2	0	84	4	6143	1658	68	483	88	655	23	2
SRS019026	69	97	2	30	427	7	17	4	34	6	2716	34	5872	20485	1262	4393	469	7564	139	40
SRS019027	47	123	4	3	1091	7	22	3	61	3	1788	12	7278	14464	662	2305	409	4328	92	32
SRS019028	30	556	39	48	7960	21	17	53	15	12	1641	114	392974	23124	1299	2482	1239	13200	336	62
SRS019029	16	348	30	155	2263	6	21	17	0	1	1455	81	168002	37948	1064	17957	818	16536	272	68
SRS019030	45	204	5	1	59	4	24	183	1	0	99817	190972	3772	3033	3896	2609	24821	3569	3161	271
SRS019033	23	4	0	0	41	30	1	6	0	0	1	15	3	6	0	1	5	1	0	0
SRS019039	4	130	6	0	9	0	2	2	0	0	2	23	16	13	0	0	11	8	0	1
SRS019045	144	475	254	6	2959	5	17	36	175	3	2439	163	8844	15432	594	1977	787	4721	140	114
SRS019063	0	210	2	1	29	0	37	5	0	0	2	13	17	5	3	7	0	123	0	7
SRS019064	0	328	10	2	76	2	69	3	0	0	2	1	9	11	11	35	1	9	12	16
SRS019067	0	61	4	0	0	0	0	0	0	0	1	1	2	0	0	1	0	0	0	0
SRS019071	124	171	63	36	5827	143	47	11	21	8	4312	79	280895	32405	3005	6439	3834	26956	1032	126
SRS019073	5	15	5	8	8237	20	5	20	1	22	596	69	5558	1450	533	356	754	8168	254	61
SRS019077	12	301	73	10	7713	5	2	21	0	0	602	67	4757	2227	590	525	791	6502	245	54
SRS019081	0	147	3	4	37	0	27	1	0	0	9	20	7	9	0	18	1	12	8	3
SRS019087	0	48	2	0	3	0	1	0	0	0	4	23	4	2	2	1	2	2	0	0
SRS019116	0	8	1	4	26	0	31	1	0	0	1	2	4	2	2	1	0	4	0	8
SRS019119	0	22	5	0	14	0	2	0	0	0	4	13	8	10	2	4	2	16	0	0
SRS019120	3	16	1	13	983	0	5	1	1	2	962	20	2766	4330	375	2077	325	3740	65	22
SRS019122	34	97	49	67	3950	71	40	43	53	17	4294	77	8644	59786	2379	4300	1487	27316	366	77
SRS019124	5	23	1	28	3078	9	27	0	1	27	578	31	634	1922	634	468	498	5762	77	79
SRS019125	1	109	0	217	2973	42	125	1	0	1	2747	183	4366	9415	3359	2345	2069	30446	410	416
SRS019126	22	53	10	36	4290	41	21	30	38	13	3546	64	6757	39436	1267	2619	1140	19011	326	55
SRS019127	23	53	27	20	1226	31	20	13	39	8	1469	75	5766	11682	858	1675	748	6387	150	33
SRS019128	22	1046	105	26	5716	14	43	13	0	4	639	65	80245	4270	958	1662	757	6804	279	42
SRS019129	14	339	39	17	1242	2	8	8	0	0	252	24	8952	5213	415	1028	254	2548	92	17
SRS019161	35	89	4	191	85	48	33	16	23	3	91210	85685	2294	3285	3038	3218	21482	4822	1481	57
SRS019215	0	1	0	0	7	0	0	0	0	0	3	7	7	4	0	1	0	2	1	0
SRS019219	103	535	218	23	971	14	62	6	26	20	2549	30	5076	52351	2550	5598	790	10880	303	50
SRS019221	6	0	0	0	237	16	0	0	2	0	7	15	109	174	5	17	9	58	6	1
SRS019225	4	1333	69	0	133	3	0	6	0	4	55	12	989	1934	54	149	92	1798	18	18
SRS019267	4	16	2	27	162	7	29	1	0	0	77315	324466	1551	2675	2272	1576	15312	1565	1427	11
SRS019327	80	130	78	92	7115	149	34	8	11	5	1994	44	24388	17736	2707	3781	3039	29092	718	55
SRS019329	0	9	2	2	7189	0	2	1	0	0	161	16	1803	539	228	119	176	1612	29	17
SRS019333	3	259	76	1	22171	2	18	12	0	0	51	7	1026	118	29	35	37	271	6	6
SRS019339	1	11	1	0	5	0	1	0	0	0	25	0	2	41	3	45	2	13	0	0
SRS019379	0	0	0	0	2	0	0	0	0	0	3	1	1	0	0	0	0	0	0	0
SRS019386	1	57	7	0	3	0	0	0	0	0	3	0	3	48	0	11	1	8	2	0
SRS019387	1	637	52	4	873	1	0	3	0	1	38	30	546	1843	51	61	52	737	38	8
SRS019389	46	9	13	2	1741	6	21	1	4	4	232	14	1312	15077	434	958	378	1627	129	24
SRS019391	0	0	0	0	417	0	0	0	0	0	2	0	23	27	1	4	2	19	1	0
SRS019397	14	326	15	1	313	30	11	68	24	0	107390	514735	3037	3991	2946	2202	99821	2815	3409	439
SRS019582	19	89	20	81	175	21	29	10	5	0	58990	66920	3486	5947	13829	7451	17236	2587	1542	420
SRS019587	10	30	11	0	8473	0	1	0	6	0	28	4	468	545	39	83	55	234	11	4
SRS019591	10	307	44	4	8193	2	0	4	0	0	107	22	628	1125	56	162	77	1227	18	35
SRS019597	4	34	2	0	30	1	0	2	0	0	1	14	1	2	0	0	7	7	10	0
SRS019600	0	0	0	0	5	0	0	0	0	0	0	0	2	1	1	1	0	1	0	0
SRS019601	65	112	9	51	119	18	35	3	0	0	26405	728454	8701	1353	1081	2339	14497	3685	1233	20
SRS019607	80	226	47	10	3508	34	27	1	23	45	3389	31	3861	46220	2473	5480	1649	10433	279	123
SRS019685	63	26	7	9	170	18	20	6	1	2	114291	195689	4523	3010	3953	3744	41300	2276	2970	53
SRS019787	4	24	2	6	14	2	5	0	0	0	28637	986129	3459	2082	1215	1613	26341	2543	1397	160
SRS019867	0	95	9	0	6	0	0	0	0	0	0	1	8	20	3	10	0	7	4	0
SRS019872	1	3	2	0	325	4	1	0	0	4	30	1	284	1158	68	92	10	183	10	7
SRS019894	94	217	175	1	8078	25	22	15	31	2	1252	95	4540	59774	3037	3700	292	5708	303	103
SRS019906	10	1498	95	23	891	6	12	5	2	0	179	48	1513	1661	315	467	320	3355	195	83
SRS019910	24	10	5	3	56	2	6	2	1	3	21680	728486	1784	2600	5539	3579	32832	1923	1044	32

SRS019968	0	118	5	68	86	3	51	2	33	0	55965	208759	6720	2688	2622	2426	79468	3260	2668	148
SRS019974	87	201	81	36	13922	45	63	2	44	2	1489	137	4839	55995	1347	2830	421	12110	165	25
SRS019976	3	4	0	1	1574	15	1	1	2	0	38	113	329	2328	30	64	46	303	10	1
SRS019980	50	1628	119	15	7234	15	26	35	0	3	1012	184	252252	68531	822	3203	648	9629	157	54
SRS019986	3	278	8	0	7	0	1	2	0	0	2	8	0	1	0	1	0	0	0	0
SRS019989	0	0	0	0	12	0	0	0	0	0	4	17	8	8	3	3	6	1	0	0
SRS020220	93	117	17	2	3063	27	28	6	46	25	2247	79	5668	58618	2330	3668	460	6853	392	37
SRS020226	60	1884	108	67	7442	12	7	33	4	2	897	128	187661	20967	1310	2381	1333	18621	535	59
SRS020232	2	154	21	0	11	0	10	1	0	0	20	0	1	44	7	23	3	11	3	0
SRS020233	110	115	85	23	164	21	67	32	5	1	92718	131162	5849	4429	4852	5247	93023	4447	3824	332
SRS020261	23	62	9	0	82	22	1	7	0	0	59	80	172	56	9	14	76	17	4	0
SRS020263	30	450	91	8	131	29	1	23	0	0	37	40	421	166	24	12	43	75	3	8
SRS020328	48	94	33	185	83	13	92	7	0	1	24948	129570	3618	1564	1815	1538	77233	2034	2026	223
SRS020334	53	417	43	17	3499	24	13	10	8	8	566	16	2155	22467	1134	1878	128	3017	114	14
SRS020336	1	3	1	0	147	0	3	0	1	0	14	3	119	501	17	65	1	119	1	0
SRS020340	27	7317	254	8	1778	8	1	6	0	0	158	21	1188	1978	141	245	164	1374	53	38
SRS020349	0	0	0	0	4	0	1	0	0	2	0	0	0	2	0	1	1	0	0	0
SRS020386	0	139	14	0	97	1	0	0	0	0	7	15	29	80	2	11	10	15	0	2
SRS020856	63	94	30	16	12446	62	60	8	13	34	3367	13	4638	82043	1692	3669	416	11438	179	239
SRS020858	2	6	0	21	795	17	3	3	1	0	56	11	198	1589	39	99	18	465	8	5
SRS020862	1	1029	18	1	1160	0	1	2	0	0	21	12	801	151	17	30	92	204	8	15
SRS020868	0	18	2	0	19	0	0	0	0	0	13	24	8	3	2	3	6	3	1	1
SRS020869	40	228	65	384	204	23	36	3	4	3	53177	81029	5033	2666	2255	2672	20211	2878	2540	387
SRS021473	4	2	3	4	529	10	2	0	0	0	101	44	2569	515	62	135	77	553	20	8
SRS021477	59	2128	127	13	4084	33	19	20	1	6	345	65	90525	1618	498	1000	628	6783	196	53
SRS021483	0	31	0	0	4	0	0	0	0	0	0	13	5	2	1	2	1	10	0	0
SRS021484	22	299	6	16	162	1	31	1	1	0	206658	90388	7243	7211	7163	6451	55807	4205	3408	536
SRS021496	80	75	62	32	9552	45	52	22	32	13	2193	39	178506	45803	2620	6812	3156	26377	767	164
SRS021948	79	116	11	28	253	12	47	18	3	1	74417	148105	7207	7211	6278	7581	52929	7425	4403	216
SRS021954	132	345	70	1	5285	17	34	23	58	9	2056	52	2960	39977	2013	2807	958	10430	240	80
SRS021960	5	1105	71	5	1412	3	1	2	1	0	179	14	4379	1534	177	510	140	2207	40	22
SRS021969	0	0	0	0	3	0	0	0	0	0	0	1	0	0	0	0	0	0	0	0
SRS022006	0	464	51	0	16	1	0	0	0	1	6	105	6	9	8	13	16	6	1	1
SRS022071	23	240	67	57	205	30	77	9	9	5	52156	472145	5883	4024	2569	3335	35236	2390	1696	267
SRS022077	111	87	218	9	9893	34	32	4	93	8	2981	241	65157	17630	1605	3129	1783	15845	465	124
SRS022079	1	3	5	0	898	5	1	0	0	0	35	137	398	57	10	22	25	103	2	0
SRS022083	8	1903	150	9	5835	3	12	15	0	2	524	151	12989	3148	398	1612	650	5518	140	28
SRS022092	0	0	0	0	5	0	0	0	0	0	0	0	0	0	0	0	0	0	0	0
SRS022129	0	40	1	0	21	0	0	0	0	0	5	19	2	4	1	3	4	0	1	0
SRS022137	13	156	11	23	162	45	14	15	9	5	164933	78817	4520	16708	4277	5853	46133	2754	2810	507
SRS022143	125	256	194	115	2550	283	129	2	11	65	5559	151	586754	55735	4984	12467	1561	13618	547	112
SRS022145	1	9	3	3	1920	4	1	0	0	0	47	7	600	545	27	189	25	341	4	4
SRS022149	8	687	64	35	2813	24	13	1	0	0	365	42	4348	21383	652	2129	378	5026	81	19
SRS022158	0	0	0	0	9	0	0	0	0	0	2	0	0	1	1	0	0	0	1	0
SRS022530	48	68	61	16	7127	18	21	9	18	1	947	119	11348	4927	1119	1476	1454	16110	309	67
SRS022532	6	7	2	11	246	9	0	1	0	0	28	19	754	1441	13	28	32	277	6	1
SRS022536	100	5959	268	13	6205	24	10	28	0	14	829	150	213203	24384	1227	2619	1797	15159	609	89
SRS022609	108	73	11	421	164	18	139	23	0	0	54483	400611	6413	26661	67206	30619	25671	18476	5826	721
SRS022621	69	68	107	11	9258	38	69	1	54	8	3474	93	10986	76435	2735	4949	1139	17321	489	145
SRS022625	10	97	6	2	3149	3	1	9	0	59	197	52	2333	3784	203	370	202	1454	51	4
SRS022645	0	4	1	0	5	0	1	0	0	0	1	11	3	3	2	1	1	2	1	0
SRS022719	69	156	57	32	11893	18	42	6	29	12	768	28	18253	25778	1248	1803	1391	15036	535	57
SRS022721	4	13	3	3	316	1	1	0	3	0	48	7	4445	9932	60	121	64	639	17	2
SRS022725	75	2382	478	86	11145	47	71	21	27	15	3698	224	1161747	73864	4753	9194	2894	29605	663	87
SRS022734	0	0	0	0	4	0	1	0	0	0	1	0	0	0	0	0	0	0	1	0
SRS023346	7	3	0	4	86	0	7	1	0	1	14451	145995	630	597	316	843	3070	798	706	3
SRS023352	150	398	106	26	3531	46	109	62	51	28	2467	13	11346	97702	2954	7499	548	10287	314	177
SRS023354	0	3	0	5	48	0	0	0	0	0	47	12	557	1233	18	292	12	165	6	2
SRS023358	9	724	67	13	1888	6	4	8	1	0	892	442	10285	5335	385	2386	276	3427	43	13
SRS023526	30	48	8	109	606	4	73	20	0	2	26372	334116	4128	37811	99728	41974	23026	3705	4100	336
SRS023534	0	0	0	2	738	0	0	0	0	0	17	180	13	58	58	40	12	40	0	0
SRS023538	3	856	34	4	1972	3	4	2	0	0	52	21	144	461	61	109	67	1023	25	122
SRS023557	47	87	5	1	7275	4	25	5	36	0	95	92	1087	10163	224	510	134	1900	70	108
SRS023583	1	34	0	0	58	0	1	0	0	0	93500	99892	3578	4243	6543	5452	17079	1745	1876	752
SRS023591	1	13	4	11	148	2	0	0	2	0	59	5	3880	1371	69	1032	34	485	6	3
SRS023595	41	2752	158	284	2856	66	28	9	6	2	3500	161	286254	29536	2979	13727	2316	24314	658	140
SRS023617	46	377	86	175	6242	19	82	8	76	1	1176	26	5783	56372	1294	5273	399	11528	157	74
SRS023835	108	151	76	15	6740	64	43	13	104	0	1257	460	8317	48521	1688	3973	972	12269	272	83
SRS023837	2	1	0	0	144	5	0	0	0	0	39	102	795	2036	37	111	45	252	8	2
SRS023847	0	164	15	0	21	0	0	0	0	0	12	162	9	58	8	40	18	11	0	0
SRS023850	0	1	0	0	23	0	0	0	0	0	23	18	12	28463	0	30	4	97	1	0
SRS023914	122	209	11	1	830	10	394	2	1	3	15469	27979	6397	18183	39599	18900	22208	6604	5086	275
SRS023926	92	387	29	19	9545	23	22	8	49	3	3723	63	87748	34474	1628	3975	1029	9826	294	69
SRS023938	41	3307	424	16	19053	49	79	448	1	0	2420	202	96790	9103	1962	4472	2808	23142	689	145
SRS023958	85	73	97	4	13076	18	36	3	24	0	10946	290	27791	18557	3925	7102	5242	40691	1103	319
SRS023964	6	689	53	8	2424	10	5	8	1	1	1080	173	12753	11594	1423	3631	821	12216	343	57
SRS023970	2	29	1	0	5	0	0	0	0	0	8	172	2	3	1	1	8	3	1	0

SRS023971	45	33	0	1	57	5	19	1	0	0	61945	425072	6940	2806	2050	2223	12100	7971	1149	61
SRS023987	0	8	2	3	4579	2	0	2	0	0	221	311	755	469	94	151	141	1329	44	5
SRS024009	6	15	39	3	128	10	6	1	0	1	204872	49143	3274	5225	5062	5387	14938	3957	4647	36
SRS024015	81	175	60	0	91598	6	32	7	117	43	552	23	4134	22208	932	2533	237	4656	129	54
SRS024017	0	9	2	3	21140	2	0	1	2	1	42	7	1274	2665	27	85	33	405	10	3
SRS024021	18	1135	125	3	165336	3	1	9	1	2	209	12	1475	2289	175	264	109	896	27	61
SRS024064	1	12	3	0	24	0	0	0	0	0	17	1	5	9	0	6	2	1	0	0
SRS024068	0	0	0	0	8	0	0	0	0	0	60	130	1	2	3	1	18	6	4	0
SRS024075	7	74	2	65	103	155	9	3	1	0	91147	48928	2504	2450	2476	2528	50882	2219	2518	119
SRS024081	147	108	33	175	9569	60	270	12	100	6	3135	96	66822	52430	2626	5294	2183	21109	586	105
SRS024087	54	1879	239	67	6031	17	16	19	6	0	1112	120	1069934	32736	2375	11722	1607	20847	435	98
SRS024132	75	75	10	70	224	8	117	7	1	0	19501	156116	2670	2863	5621	2232	37394	2295	950	123
SRS024138	63	61	89	47	22922	19	24	6	52	0	960	334	2654	38505	854	1898	667	7973	169	98
SRS024140	7	5	1	28	1049	17	1	1	0	0	559	4152	205	264	160	89	1070	771	35	5
SRS024144	4	777	44	3	3399	4	0	1	0	0	1115	32	73284	2245	282	842	130	2708	29	25
SRS024265	1	3	0	0	3	0	1	0	0	0	2090	3839	51	93	166	136	4060	58	126	15
SRS024277	83	126	77	3	34080	103	42	0	55	7	2315	158	7257	39841	1936	4472	2151	14926	396	161
SRS024289	27	1046	116	12	4446	1	5	8	0	10	575	184	379654	3473	685	1822	433	4403	114	78
SRS024301	0	17	1	0	3	1	0	0	0	0	34	144	6	4	2	1	17	4	0	0
SRS024318	102	232	56	50	2257	16	70	8	23	4	3837	98	6404	61284	3780	9436	1936	9829	561	81
SRS024331	7	205	6	12	148	3	53	86	25	0	102201	114653	3724	4478	3493	3090	61777	3534	23388	268
SRS024347	4	14	1	4	550	3	5	2	1	0	152	64	772	2649	144	470	134	1198	34	2
SRS024355	35	9962	346	58	3970	15	10	56	0	22	1122	141	283084	37411	1615	3201	1846	14343	651	52
SRS024375	303	69	61	5	17662	60	136	1	29	0	1118	19	15065	6676	1743	1815	3102	18878	535	51
SRS024377	0	0	0	2	404	1	1	2	1	0	12	6	86	36	5	11	7	74	4	6
SRS024381	45	2050	127	0	1422	14	10	20	0	3	146	30	44010	2101	176	456	243	3990	92	23
SRS024388	10	464	8	2	102	7	3	1	0	0	127077	92541	4212	5284	5609	4243	15382	4477	5305	528
SRS024424	1	54	6	0	40	0	38	0	0	0	6	7	10	4	2	3	0	38	0	0
SRS024428	0	1	0	0	7	0	0	1	0	0	0	0	0	0	0	0	0	0	0	0
SRS024435	167	89	233	36	190	16	111	22	3	8	21612	452578	6757	5239	7132	8970	27847	3907	3007	300
SRS024441	109	79	148	22	10171	63	78	6	83	14	1777	55	38674	29905	1958	4487	1984	21241	480	83
SRS024447	35	615	90	17	6867	11	59	30	4	4	930	64	29195	6315	857	1665	1059	6910	275	64
SRS024470	1	6	0	11	1117	6	11	6	1	0	108	19	1373	3790	66	589	88	12801	16	7
SRS024482	17	7	3	0	108	17	0	4	0	0	16	15	767	160	10	55	4	194	2	0
SRS024549	5	16	3	0	260	8	0	174	2	1	2062	72105	231	399	404	188	4322	387	562	5
SRS024557	2	2	3	5	2304	2	0	0	0	0	1	0	450	268	4	7	1	19	0	2
SRS024561	15	1988	91	30	2515	17	17	11	0	3	450	85	61060	56640	460	2267	487	5142	184	89
SRS024567	0	3	1	0	15	0	0	0	0	0	1	0	0	0	0	0	0	0	0	0
SRS024580	88	123	23	116	19645	18	257	3	54	3	1169	2	7706	15868	456	740	204	2467	48	58
SRS024596	7	14	2	2	40	5	0	6	0	0	1	6	5	3	2	117	3	3	0	80
SRS024598	6	11	3	1	36	3	1	59	0	0	3	3	79	11	0	25	0	2	0	213
SRS024620	0	32	4	0	1	0	4	0	0	0	4	3	3	8	0	0	0	0	0	0
SRS024625	55	14	3	0	87	1017	3	1	5	0	115311	167135	4727	3542	4097	3339	48085	3285	10815	141
SRS024637	40	148	36	0	822	22	5	12	112	0	1634	153	5239	50479	2278	3199	200	5376	420	4
SRS024649	44	1917	95	31	875	9	10	6	3	0	699	89	47487	33806	733	1946	433	3198	173	38
SRS024655	0	44	8	0	13	0	0	0	0	0	24	38	8	20	4	5	11	3	4	0
SRS042131	129	205	119	1	7244	33	51	7	5	4	824	18	7627	9995	564	2007	563	6132	185	115
SRS042284	17	10	1	0	85	7	1	6	1	0	95792	18080	674	2583	2614	2379	3860	1140	1557	8
SRS042428	0	0	0	1	7	0	0	0	0	0	0	0	1	1	0	0	0	1	0	0
SRS042457	3	0	2	3	578	1	1	1	1	0	35	12	913	1813	24	89	30	1565	8	3
SRS042628	18	55	20	13	24	10	26	10	0	0	22873	30623	1110	1600	999	1247	21398	1048	619	83
SRS042643	282	94	81	101	1134	112	269	5	9	67	1639	124	395059	59459	2962	9161	675	10155	391	92
SRS042858	0	0	0	0	3	0	0	0	0	0	0	3	0	0	0	0	2	0	0	0
SRS042910	120	128	13	4	11178	9	57	14	43	9	1904	81	27352	41828	3221	3631	5600	39263	976	14
SRS042984	72	5733	389	26	6293	19	24	20	1	30	786	146	82977	10160	1111	1945	1013	10148	419	179
SRS043001	3	128	9	0	67	2	2	4	0	1	129168	144385	2372	2883	2791	2619	12122	1965	1723	154
SRS043018	11	2682	142	30	1176	11	6	3	1	0	303	47	27657	2056	222	480	268	3994	184	28
SRS043411	24	51	9	12	20	13	21	7	0	2	76823	583383	2891	2359	1977	1925	6688	5416	732	15
SRS043646	2	17	4	3	213	1	1	0	0	0	43	16	1997	3006	28	241	33	367	9	1
SRS043663	141	41	42	24	28843	13	73	17	163	1	9931	224	4933	49116	1617	6125	1583	25957	206	190
SRS043676	1	14	11	4	3497	23	1	4	1	0	49	16	2612	442	42	73	133	869	15	2
SRS043701	12	22	14	3	210	4	33	2	0	2	3513	321222	2382	1762	978	1144	32124	940	960	21
SRS043755	21	984	69	3	1189	3	10	4	1	0	73	18	3971	513	92	157	119	1401	93	54
SRS043772	5	1964	74	14	1601	8	0	8	0	0	103	36	74885	635	156	188	222	1784	138	22
SRS044373	108	207	64	28	7843	43	44	22	50	0	1647	41	5697	35860	2330	4574	1552	10149	343	54
SRS044474	7	267	26	0	29	0	1	3	0	0	3	0	6	0	0	4	2	6	0	1
SRS044486	131	96	16	4	7088	30	7	11	104	6	622	2	1088	16414	1004	1451	233	2062	115	17
SRS044626	3	37	3	0	8	0	0	0	0	0	10	9	2	5	3	2	9	2	4	0
SRS044662	30	177	34	16	8593	22	11	4	6	6	1073	24	16619	13259	1226	2006	1737	17921	360	33
SRS044742	0	0	0	0	1	0	0	0	0	0	0	0	0	5	1	4	0	0	0	0
SRS045004	140	162	22	17	282	21	39	276	1	1	150913	164738	7116	6846	7390	15802	65008	5004	5520	242
SRS045049	4	82	5	0	1813	6	0	13	0	0	170	10	9624	694	210	149	290	2256	81	24
SRS045127	61	47	17	9	3276	37	24	1	34	0	2241	115	10171	17176	697	2038	609	6956	126	48
SRS045197	31	1588	110	57	4314	62	10	10	1	2	501	104	9765	13037	1006	4092	489	16695	300	137
SRS045254	3	21	6	1	403	4	1	1	0	0	50	36	5812	4205	38	153	33	326	8	5
SRS045262	3	12	8	1	5964	17	2	25	0	0	27	1	914	230	28	48	87	1037	12	4
SRS045313	2	81	5	76	2154	6	1	2	1	0	573	35	854	18026	293	1147	81	25876	31	16

SRS045606	0	55	10	0	25	1	0	0	0	0	2	0	61	15	32	0	8	8	0	0
SRS045645	62	125	19	52	298	21	29	106	0	4	86904	194046	4942	4618	4536	10043	43544	3599	3425	219
SRS045713	11	6	12	4	72	13	2	43	0	0	23094	215691	5979	11025	20400	12813	22232	6171	3723	243
SRS045715	107	197	75	16	2335	40	98	10	21	25	2149	14	48981	122242	3338	8689	626	13044	306	353
SRS045978	4	23	3	5	1056	6	3	3	0	13	58	2	1869	334	51	46	65	345	23	1
SRS046344	0	83	0	0	2	0	0	0	0	0	0	0	0	0	7	2	0	4	0	11
SRS046623	3	2	2	7	546	5	1	4	1	0	116	41	2027	3517	72	462	74	2076	16	6
SRS046686	15	28	1	0	11491	8	0	87	0	28	102	4	196	176	141	77	172	770	16	21
SRS046688	0	3	0	1	71	0	1	1	0	0	23	7	17	12	13	8	4	1211	5	1
SRS047014	20	131	7	191	70	26	43	2	7	3	64560	174002	1966	2352	2272	1887	52229	1510	1688	137
SRS047044	8	14	3	24	54	7	28	0	1	0	24815	43507	3978	59575	147884	44339	15312	9151	7019	703
SRS047100	2	3738	214	3	693	1	0	4	0	0	261	54	1124	1355	166	239	181	1692	31	27
SRS047113	58	1349	97	38	4941	43	22	55	5	8	1277	141	107143	8965	1022	2777	1052	21012	404	98
SRS047210	118	81	52	7	3200	19	57	3	63	0	976	50	38195	37398	1561	2967	801	7389	268	64
SRS047219	207	165	118	4	2131	26	43	3	34	13	3561	49	24299	46062	3343	7295	682	6156	353	44
SRS047225	16	273	10	0	51	0	0	0	0	0	4	78	22	9	1	5	9	11	10	0
SRS047265	2	197	14	9	549	4	0	1	0	0	27	13	1528	75	48	33	29	11113	22	1
SRS047335	0	0	0	0	14	1	0	0	0	0	3	4	0	5	0	5	3	8	0	0
SRS047634	75	1146	97	32	11039	45	36	35	9	26	2233	197	263339	19007	2253	7307	1498	30105	534	166
SRS047824	22	638	58	11	947	16	40	0	21	16	2757	27	362482	46456	4112	8357	1072	8041	374	74
SRS047844	0	6	0	0	5	0	0	0	0	0	0	0	0	0	0	0	1	1	0	0
SRS048164	10	137	6	123	386	9	12	39	0	2	94268	91226	4111	9122	6457	9931	22085	4414	5922	238
SRS048411	177	162	38	8	1735	47	97	8	9	11	2663	26	9463	45375	3028	7573	681	13795	356	81
SRS048719	3	12	7	1	277	0	0	0	2	0	10	20	243	234	47	46	9	52	6	4
SRS048791	29	225	118	46	2415	49	25	8	18	35	1672	13	12858	47971	1449	5218	389	6769	235	70
SRS048870	19	41	51	110	263	13	25	18	0	0	161293	226482	5691	4140	3661	3842	27582	2863	3587	86
SRS049147	160	230	39	31	4841	10	65	8	116	1	3008	47	7833	34804	1893	4474	2027	15761	320	51
SRS049237	0	0	0	0	4	0	0	25	0	0	9	3	0	0	0	0	0	0	0	0
SRS049268	39	1891	157	12	4495	13	20	6	0	6	1776	123	111679	84142	2327	6867	1980	21454	467	48
SRS049283	2	8	0	0	34	0	0	1	0	0	27	7	3691	375	38	96	29	125	4	0
SRS049318	82	479	33	133	4378	90	13	18	10	20	6841	139	592585	42974	3549	10207	2270	35514	719	150
SRS049389	98	79	69	104	17264	102	33	9	13	1	4636	172	69563	13773	2673	5504	3297	22356	841	327
SRS049712	12	27	16	21	98	14	7	3	0	3	23551	83507	2886	51665	149629	94479	22398	12471	4875	647
SRS049744	2	2	0	0	13	0	0	0	0	0	3	0	19	14	2	4	2	37	0	0
SRS049900	231	104	18	34	576	30	8	11	1	1	84138	618807	9637	8387	5987	3960	25762	3215	7803	87
SRS049959	102	112	158	80	377	14	23	4	2	0	9124	61737	2862	16471	33906	15741	13965	3340	2551	364
SRS049995	88	55	28	96	181	71	30	10	33	5	39581	71811	7979	13300	28167	12025	62929	4866	4526	332
SRS050007	0	1	0	0	142	0	0	0	0	0	6	11	166	202	5	15	7	67	2	2
SRS050025	0	76	11	0	13	1	0	0	0	0	1	0	28	36	4	9	5	9	0	0
SRS050029	8	19	3	0	15199	0	1	2	9	0	337	41	2893	1236	287	498	691	3873	79	15
SRS050184	0	3	0	0	23	0	0	0	0	0	5	7	130	1	1	0	0	8	0	0
SRS050244	191	363	107	50	4452	92	86	32	40	6	2710	59	540559	32196	2438	6765	1480	17621	620	100
SRS050299	3	25	1	4	20	19	26	1	8	1	41158	263138	1825	1527	1584	1933	32729	1738	1531	41
SRS050422	38	87	31	2	140	15	21	32	3	15	33266	156228	1667	5825	2567	6938	10374	959	1035	26
SRS050484	2	15	4	0	17	0	0	0	0	0	14	138	5	13	19	15	11	4	2	0
SRS050628	7	42	8	8	1281	11	3	3	2	1	474	14	113567	1022	232	561	205	2181	47	14
SRS050669	55	122	127	11	15332	48	32	0	155	4	1822	170	48097	9243	2549	3156	4423	33097	1079	116
SRS050752	45	44	12	50	102	4	100	5	7	5	24866	223877	6721	12367	31423	10925	52590	2791	2358	409
SRS050925	22	126	10	37	111	3	21	32	0	0	84459	189158	6135	3479	3172	8644	19911	2692	2532	234
SRS051031	126	132	3	885	256	18	86	10	0	1	98362	155282	2413	6657	12944	9806	93283	5762	5419	515
SRS051116	1	3	0	3	482	0	0	2	0	0	23	0	1201	712	17	327	21	308	9	2
SRS051244	11	2035	104	14	1544	2	10	13	0	1	450	52	58614	4567	455	1462	647	6293	189	12
SRS051378	4	161	25	8	2179	4	1	3	2	0	152	85	12526	1630	358	632	288	2044	111	8
SRS051505	1	1	0	0	10	0	0	0	0	0	0	0	1	0	0	1	0	1	0	0
SRS051600	0	5	0	0	10	0	0	0	0	0	0	0	1	1	0	0	0	1	0	0
SRS051613	1	65	7	0	5	0	0	1	0	0	1	7	24	7	0	0	0	2	0	0
SRS051791	56	458	28	10	1151	68	39	5	43	2	1678	20	20214	48719	2943	5675	503	17983	288	29
SRS051882	69	110	9	4	62	7	27	60	0	0	52550	520048	5556	2019	2093	2184	24818	2687	2743	30
SRS051930	19	2031	178	20	2990	17	15	28	0	2	514	49	71630	7904	716	2224	546	5647	116	26
SRS051941	18	3162	174	31	10704	19	4	6	0	4	239	48	74881	2688	676	1089	507	10707	177	41
SRS052027	37	27	19	30	128	18	43	46	78	0	26235	391960	1052	1845	1557	1274	28151	1476	2050	85
SRS052227	31	110	75	5	4174	10	23	12	21	0	2210	119	11991	30477	2306	3342	2629	16362	437	126
SRS052330	0	0	0	0	22	0	0	0	0	0	0	0	0	0	0	0	0	0	0	0
SRS052590	0	112	8	0	5	0	0	0	0	0	0	0	0	0	0	0	0	1	0	0
SRS052604	35	928	58	6	1504	8	1	7	2	2	290	26	229384	5193	597	1282	488	3815	193	28
SRS052620	0	0	0	0	6	0	0	0	0	0	0	0	0	1	0	1	0	1	0	0
SRS052668	3	28	5	24	1546	6	0	3	120	0	224	148	11621	6131	149	257	278	2287	57	4
SRS052697	96	119	34	27	108	10	67	33	5	0	108696	82447	2650	3263	3983	6575	30900	3352	2732	102
SRS052756	0	0	0	0	6	0	0	0	0	0	1	5	0	1	2	1	5	3	0	0
SRS052874	1	7	1	16	1095	9	0	21	0	12	106	6	1024	1225	85	141	91	694	18	8
SRS052876	38	1981	87	60	878	8	19	9	0	0	644	158	192959	3066	797	1174	1037	11950	603	72
SRS052988	0	42	5	0	2	0	5	0	0	0	0	1	44	23	0	3	0	14	0	7
SRS053335	9	35	19	5	79	4	18	6	1	1	79894	53222	3114	3053	3304	1952	9642	1653	4333	70
SRS053398	139	57	11	3	144	1	4	3	3	4	26462	238408	4447	20898	54035	33239	61790	5445	4400	390
SRS053437	12	73	8	0	31	1	2	1	0	0	5	6	9	15	1	3	6	4	0	0
SRS053584	4	721	109	6	1364	4	5	34	0	1	258	59	1461	685	128	258	162	1602	55	11
SRS053603	57	154	144	54	738	23	10	2	23	15	1741	23	105430	28636	1876	5444	1037	8479	142	56

SRS053854	146	151	83	102	1446	34	23	15	82	0	3496	23	1441	50108	1736	3060	477	13027	142	48
SRS054061	1	210	12	0	1	0	0	0	0	0	1	0	13	14	3	3	0	0	0	0
SRS054430	10	360	19	8	4488	5	8	24	1	0	301	11	1328	2960	290	1010	212	2127	73	27
SRS054569	18	38	12	4	3216	37	29	18	6	0	213	87	2560	660	186	217	283	2863	50	4
SRS054590	3	93	0	0	140	11	2	74	0	0	4685	714310	1450	1849	414	797	21363	272	442	2
SRS054653	16	1520	224	1	668	1	3	9	0	0	73	15	2027	1545	129	202	185	852	41	5
SRS054687	82	189	48	19	4569	11	27	10	59	0	1117	19	9018	11135	705	1365	592	4634	152	68
SRS054776	11	106	17	2	975	6	1	1	0	9	118	13	2968	1137	88	158	91	660	27	8
SRS054956	10	53	65	4	269	3	15	6	19	0	76467	73325	4467	1256	1903	1754	3397	932	1541	88
SRS054962	2	0	2	0	13	0	0	1	0	0	1	16	11	14	0	2	1	5	0	0
SRS055118	0	13	7	0	48	1	0	0	0	0	28	2	5660	87	36	33	42	369	2	0
SRS055298	0	0	0	0	7	1	0	0	0	0	4	2	37	10	1	0	2	4	0	0
SRS055378	51	1468	64	57	2453	23	36	8	0	0	2338	150	894372	19085	3541	8842	3132	20096	661	39
SRS055401	26	395	85	19	1949	2	4	20	0	1	722	42	4890	1697	354	795	282	5062	118	29
SRS055426	82	57	14	13	2759	12	25	8	28	3	601	39	14660	15714	632	2169	326	5388	113	26
SRS055450	20	793	78	22	590	24	13	7	1	2	190	238	180351	1972	312	639	261	2394	100	10
SRS055982	22	279	10	7	181	45	3	14	3	0	146623	102271	5484	25995	4224	6032	54296	3187	3756	730
SRS056210	0	0	0	0	7	0	0	0	0	0	1	1	0	0	0	0	1	1	0	0
SRS056259	21	158	11	7	74	14	63	8	0	28	86213	71956	3846	2237	1844	3431	46193	3393	2794	119
SRS056323	73	273	105	2	796	18	18	2	110	2	868	12	1856	34216	1623	2256	158	4162	297	56
SRS056519	5	89	15	11	99	4	13	19	1	0	66299	405412	5701	3362	2439	3199	18296	2791	2235	57
SRS056622	23	126	84	61	3420	36	114	1	59	19	1228	20	274955	27435	1853	4204	1018	5354	398	55
SRS056695	0	0	0	0	4	0	0	0	0	0	2	7	0	4	0	1	0	0	0	0
SRS056892	5	33	17	1	5734	6	0	14	1	0	139	8	5423	5646	144	322	190	850	28	7
SRS057022	7	24	6	18	1194	37	7	4	3	0	610	99	7597	31460	273	1484	153	2344	32	16
SRS057083	0	16	0	0	3	0	0	0	0	0	2	6	10	9	7	0	0	9	0	1
SRS057205	40	42	87	43	4008	27	37	2	31	10	6392	42	69203	21991	3194	7821	2769	14517	561	169
SRS057355	33	48	6	7	4072	18	8	2	8	0	1330	54	24914	8658	1103	1688	1395	12275	289	61
SRS057539	75	341	25	12	13098	19	107	5	58	19	854	20	5180	36217	1060	2928	718	5187	123	62
SRS057692	109	62	94	31	21882	113	808	12	277	1	1732	112	28064	5554	2382	2206	3175	44864	483	51
SRS057717	37	6	3	3	103	3	4	7	1	0	8828	189689	645	571	268	499	3461	792	786	2
SRS057791	85	310	156	7	4107	74	38	12	50	9	1850	26	3359	32444	2224	5588	1015	8726	277	85
SRS057807	0	0	0	0	13	0	0	0	0	0	2	2	1	7	0	2	0	4	0	0
SRS058053	34	1981	167	80	3257	12	12	13	1	5	1198	103	68385	49998	1335	12776	527	14228	140	51
SRS058105	7	1	0	3	5309	0	0	2	0	0	45	2	663	111	27	37	28	932	10	0
SRS058182	37	34	14	1	157	3	0	17	0	0	18	24	26	35	67	8	14	186	4	2
SRS058213	0	345	4	0	17	0	0	1	0	0	2	6	12	16	3	3	6	6	1	0
SRS058221	0	46	11	0	7	2	2	0	0	0	1	0	37	177	2	10	2	11	0	4
SRS058336	52	73	35	18	15888	46	31	4	23	4	5242	82	13023	12498	1512	3519	2248	8994	459	216
SRS058723	5	11	1	5	164	5	14	2	0	0	21529	1704007	484	662	1148	777	7034	466	1781	6
SRS058770	72	163	1	2	136	5	15	0	45	0	148967	260207	11628	6669	6513	4934	40508	4046	4814	279
SRS058808	38	768	71	114	2297	29	30	22	1	1	2456	79	170315	9783	1194	3496	1211	18135	542	34
SRS062427	2	6	2	1	16	3	13	0	0	1	13356	60832	2541	667	1096	392	6349	948	1459	11
SRS062520	0	0	0	0	9	0	0	0	0	0	0	0	0	2	0	0	1	0	1	0
SRS062540	53	53	7	6	730	5	2	3	27	0	462	232	2018	10342	530	1328	151	2514	91	12
SRS062544	188	122	5	2	1925	19	66	13	30	5	3593	47	186849	70962	4329	10605	1017	12850	463	69
SRS062713	0	62	9	0	8	2	0	1	0	0	3	18	5	3	0	0	9	10	22	1
SRS062752	1	0	0	0	15	0	0	0	0	0	20	37	1	2	2	4	17	11	1	0
SRS062761	215	54	65	32	17422	55	70	12	369	1	5376	665	7795	68211	2563	8012	3412	33288	239	144
SRS063035	1	100	10	0	43	0	0	0	0	0	4	2	58	20	0	2	7	34	2	0
SRS063040	34	99	53	35	177	74	47	10	50	6	99324	195849	3246	2365	3111	3163	23078	1942	1475	96
SRS063178	1	4	1	0	5	0	1	0	0	0	1	1	0	4	2	8	1	0	0	0
SRS063193	133	69	21	5	11820	15	62	17	29	3	666	71	67119	11841	1114	1896	1003	7467	291	46
SRS063215	27	1391	79	12	305	9	5	8	0	1	654	96	55510	6117	646	3897	542	9216	69	6
SRS063272	0	16	1	2	1287	4	0	6	7	0	128	17	304	478	218	108	171	1661	44	16
SRS063287	7	51	53	1	4887	10	0	1	9	0	70	16	2562	1247	40	166	27	431	16	0
SRS063288	63	24	21	15	8480	49	28	11	18	0	1013	70	17129	6494	679	1048	889	10798	244	71
SRS063351	6	1	3	23	76	0	1	0	1	0	431	93	855	6744	225	1176	129	2981	27	4
SRS063417	0	0	0	0	5	0	0	0	0	0	1	0	0	2	0	1	0	0	0	0
SRS063478	16	5	3	0	346	3	1	0	1	0	65	49	5363	675	73	127	69	483	27	3
SRS063603	41	830	89	168	4927	25	47	12	0	4	8403	85	322284	17114	1879	4662	1663	31263	444	182
SRS063932	121	1475	164	143	13703	60	27	46	7	6	1125	138	326199	40210	1563	4875	1592	24386	631	139
SRS063985	11	69	8	83	212	126	34	6	223	2	20044	72368	2930	2324	5410	3615	14700	4167	2294	390
SRS063999	54	3617	139	84	4132	19	35	8	0	11	1397	157	653625	10325	1547	4174	1313	14988	459	105
SRS064276	67	101	7	20	92	33	68	10	1	4	26188	37796	5087	1431	1483	1673	23357	1688	1270	325
SRS064376	0	0	0	0	9	0	0	0	0	0	28	40	2	2	4	2	28	14	2	0
SRS064423	151	135	148	37	2468	47	56	14	9	31	2131	21	37414	37604	1533	3446	477	4432	216	56
SRS064449	25	2104	264	19	4573	11	23	21	14	3	473	49	43611	4544	439	3176	381	8397	177	85
SRS064493	1	717	75	0	120	0	0	0	0	0	3	2	230	53	0	4	6	16	2	1
SRS064557	48	16	10	1	169	6	10	24	0	1	221934	795043	6750	4703	5943	5072	65713	2666	2404	75
SRS064645	23	16	0	0	77	1	0	1	0	1	15766	129701	1306	1147	869	786	4167	562	1106	19
SRS064704	0	0	0	0	11	1	0	0	0	0	4	7	1	1	0	1	1	137	0	0
SRS064774	190	77	10	13	3613	71	99	1	54	25	1351	20	3207	39840	1820	4916	1023	7000	193	86
SRS064809	1	10	2	1	484	9	1	4	0	9	41	42	1011	385	34	50	30	382	10	4
SRS065099	39	3378	161	21	2514	10	9	16	0	3	229	34	56556	10244	231	695	199	2101	125	51
SRS065133	0	0	1	2	74	2	0	0	0	0	6	17	74	133	3	6	3	145	0	0
SRS065142	1	7	1	0	6	0	0	0	0	0	22	69	5	3	1	1	13	1	0	0

SRS065179	0	3	0	0	3	0	0	0	0	0	3	17	1	6	0	14	2	7	0	0
SRS065278	131	147	182	27	2503	32	45	17	9	16	1916	20	40227	33009	1479	3208	455	3895	234	45
SRS065310	8	551	36	10	1597	4	3	0	1	3	72	52	34735	872	51	53	91	1145	47	8
SRS065335	4	2	4	2	338	7	16	0	0	5	340	16	1721	4228	380	776	253	2105	82	12
SRS065347	0	1	0	0	11	0	0	0	0	0	0	2	2	0	0	0	0	1	0	0
SRS065431	0	0	2	2	32	0	0	0	0	0	1	1	535	47	1	3	0	12	0	0
SRS065504	26	44	7	17	114	4	55	11	0	3	104676	273893	8181	2248	2252	1831	55985	4917	869	98
SRS075406	9	13	5	1	304	9	3	2	0	0	33	97	393	443	11	33	37	281	7	2
SRS075410	30	119	276	0	6578	6	7	43	10	0	69	53	1418	435	39	57	125	26619	39	47
SRS077730	17	56	3	23	89	8	7	2	0	0	61908	469195	6137	1733	2557	2071	70354	4550	1639	119
SRS077736	129	39	3	3	11398	2	42	11	140	2	191	47	3554	2958	243	749	575	3132	208	109
SRS077738	4	2	2	0	53	0	1	0	0	0	1	4	68	270	3	10	16	39	1	0
SRS077751	0	0	0	0	7	0	0	0	0	0	0	0	0	0	0	0	0	1	0	0
SRS078176	16	20	8	53	208	47	28	6	36	5	21872	49751	4454	50860	123458	37251	27349	4227	5369	691
